# Supplementary material for: Monocytes can efficiently replace all brain macrophages and fetal liver monocytes can generate bona fide SALL1+ microglia
Source: Immunity. 2025 May 13;58(5):1269–1288.e12. doi: 10.1016/j.immuni.2025.04.006 (PMC12094688; doi:10.1016/j.immuni.2025.04.006)
Supplement: Document S1. Figures S1–S7 and Tables S1, S2, and S4 [file mmc1.pdf]

Immunity, Volume *58*

Supplemental information

Monocytes can efficiently replace all brain macrophages and fetal liver monocytes can generate bona fide SALL1^{+} microglia

Jonathan Bastos, Carleigh O'Brien, Mónica Vara-Pérez, Myrthe Mampay, Lynn van Olst, Liam Barry-Carroll, Daliya Kancheva, Sophia Leduc, Ayla Line Lievens, Leen Ali, Vladislav Vlasov, Laura Meysman, Hadis Shakeri, Ria Roelandt, Hannah Van Hove, Karen De Vlaminck, Isabelle Scheyltjens, Fazeela Yaqoob, Sonia I. Lombroso, Maria Breugelmans, Gilles Faron, Diego Gomez-Nicola, David Gate, F. Chris Bennett, and Kiavash Movahedi

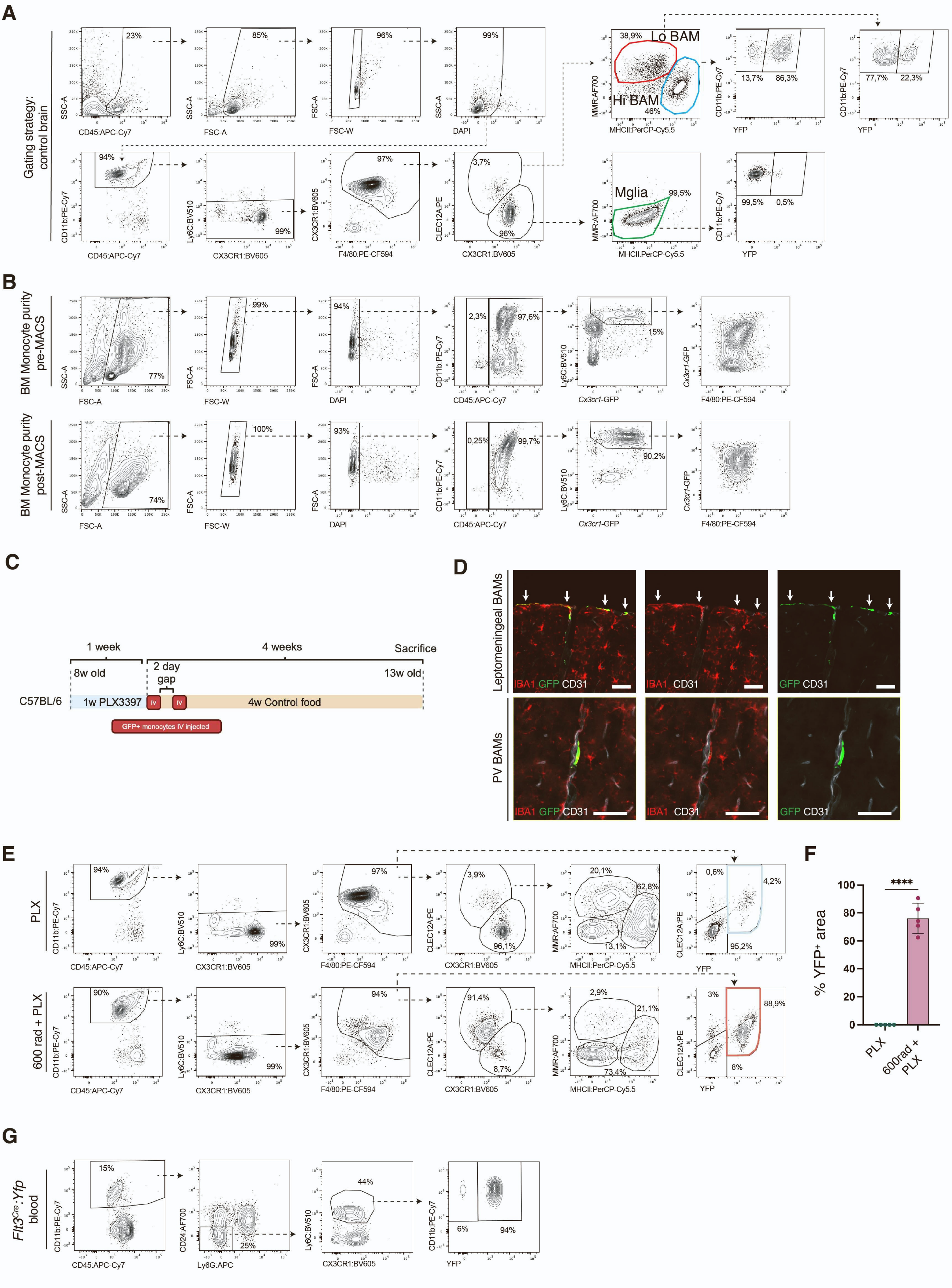
A
Gating strategy: control brain
23%
85%
96%
99%
CD45:APC-Cy7
FSC-A
FSC-W
DAPI
SSC-A
Lo BAM
Hi BAM
38,9%
46%
MMR:AF700
MHCII:PerCP-Cy5.5
13,7%
86,3%
77,7%
22,3%
CD11b:PE-Cy7
YFP
94%
99%
97%
3,7%
96%
Mglia
99,5%
0,5%
Ly6C:BV510
CX3CR1:BV605
F4/80:PE-CF594
CLEC12A:PE
B
BM Monocyte purity pre-MACS
BM Monocyte purity post-MACS
77%
99%
94%
2,3%
97,6%
15%
74%
100%
93%
0,25%
99,7%
90,2%
Cx3cr1-GFP
CD45:APC-Cy7
F4/80:PE-CF594
C
1 week
4 weeks
Sacrifice
8w old
2 day gap
13w old
C57BL/6
1w PLX3397
IV
4w Control food
GFP+ monocytes IV injected
D
Leptomeningeal BAMs
PV BAMs
IBA1 GFP CD31
IBA1 CD31
GFP CD31
E
PLX
600 rad + PLX
94%
99%
97%
3,9%
96,1%
20,1%
62,8%
13,1%
0,6%
4,2%
95,2%
90%
94%
91,4%
8,7%
2,9%
21,1%
73,4%
3%
88,9%
8%
MMR:AF700
MHCII:PerCP-Cy5.5
CLEC12A:PE
YFP
F
% YFP⁺ area
****
100
80
60
40
20
0
PLX
600rad + PLX
G
Flt3Cre:Yfp blood
15%
25%
44%
6%
94%
CD24:AF700
Ly6G:APC
Ly6C:BV510
CX3CR1:BV605
CD11b:PE-Cy7
YFP

Figure S1

Figure S1: Detection of YFP-labelled cells in *Flt3*^{Cre}:*Yfp* mouse brains and blood , related to Figure 1.

A) Flow cytometric gating strategy for the identification of YFP$^+$ Mglia, Lo BAMs, and Hi BAMs in *Flt3*^{Cre}*:Yfp* control and depleted-repopulated brains and leptomeninges. Experimental paradigm described in Figure 1A. Data representative of control: n = 3 and PLX3397-treated: n = 3. Mice were 14-week-old males, from 2 independent experiments. **B)** Flow cytometry plots revealing the purity of *Cx3cr1*$^{GFP/+}$ monocytes from adult bone marrow before and after MACS purification, n=1 from 1 experiment. **C)** C57BL/6 mice were treated for 1 week with PLX3397 chow. Afterwards, normal chow was given for 4 weeks and on the same day mice were intravenously injected with 2 million GFP$^+$ monocytes. Two days later, mice were re-injected with 2 million GFP$^+$ monocytes. n = 4 C57BL/6 mice 13-week-old males, from 1 experiment. **D)** Representative images of mice in C. Slides stained with anti-IBA1 (red), anti-GFP (green), and anti-CD31 (white). Scale bar, 50 μm. **E)** Flow cytometric gating strategy to identify YFP$^+$CLEC12A$^+$ Mo-Mglia engraftment in *Flt3*^{Cre}*:Yfp* male mice. Experimental paradigm described in Figure 1D. Data representative of 0 rad: n = 5, 200 rad: n = 6, 400 rad: n = 7, 600 rad: n = 6, 800 rad: n = 6. Mice were 10- to 16-week-old males, from 2 independent experiments. Cells were pre-gated as CD45$^+$ live single cells as shown in A. **F)** Surface area analysis of brain cryosections derived from Figure 1F. Brain surface area of YFP$^+$ Mo-Mglia was quantified. n = 5 PLX and n = 5 600 rad + PLX, 10-week-old males, from 2 independent experiments. Mean ± SD. Significance was determined using an unpaired two-tailed *t*-test. ns, *p < 0.05, **p < 0.01, ***p < 0.001, ****p < 0.0001 **G)** Gating strategy to identify YFP$^+$ monocytes in *Flt3*^{Cre}*:Yfp* mouse blood. Cells were pre-gated as CD45$^+$, single, live cells. n=4, from 1 experiment.

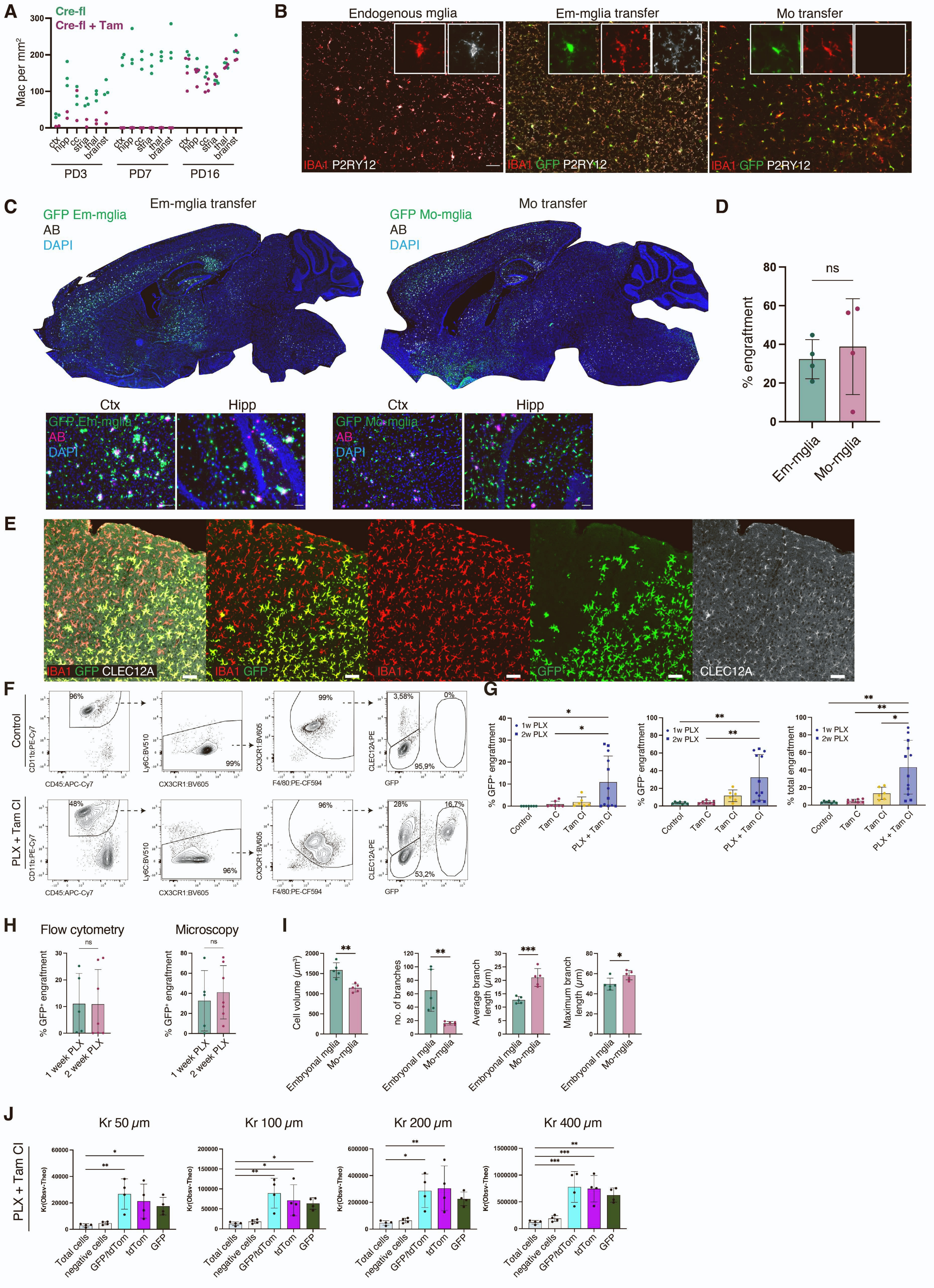
A
Cre-fl
Cre-fl + Tam
Mac per mm²
PD3
PD7
PD16
B
Endogenous mglia
Em-mglia transfer
Mo transfer
IBA1 P2RY12
IBA1 GFP P2RY12
C
Em-mglia transfer
Mo transfer
GFP Em-mglia
GFP Mo-mglia
AB
DAPI
Ctx
Hipp
D
% engraftment
ns
Em-mglia
Mo-mglia
E
IBA1 GFP CLEC12A
IBA1 GFP
IBA1
GFP
CLEC12A
F
Control
PLX + Tam Cl
CD11b:PE-Cy7
CD45:APC-Cy7
Ly6C:BV510
CX3CR1:BV605
F4/80:PE-CF594
CLEC12A:PE
GFP
G
1w PLX
2w PLX
% GFP⁺ engraftment
% GFP⁻ engraftment
% total engraftment
Control
Tam C
Tam Cl
PLX + Tam Cl
H
Flow cytometry
Microscopy
% GFP⁺ engraftment
1 week PLX
2 week PLX
I
Cell volume (µm³)
no. of branches
Average branch length (µm)
Maximum branch length (µm)
Embryonal mglia
Mo-mglia
J
PLX + Tam Cl
Kr 50 µm
Kr 100 µm
Kr 200 µm
Kr 400 µm
Kr(Obsv-Theo)
Total cells
negative cells
GFP/tdTom
tdTom
GFP

Figure S2

Figure S2: Temporary microglia absence allows monocytes to efficiently engraft the brain parenchyma, related to Figure 2 and Figure 3.

A) Same experimental setup as in Figure 2A. *Cx3cr1*^{CreER}:*Csf1r*$^{fl/fl}$ neonates were either untreated or were injected with Tam. Depletion and repopulation was followed up at PD3, PD7, and PD16 by analyzing macrophage density per mm^2.PD3: n= 3 untreated, n = 2 Tam. PD7: n= 3 untreated, n = 3 Tam. PD16: n= 4 untreated, n = 3 Tam. PD3: 2, PD7: 2, and PD16: 3 independent experiments. **B)** Representative images of *Cx3cr1*^{CreER}:*Csf1r*$^{fl/fl}$ brains retrieved at PD16. Same experimental setup as Figure 2A). Mice were either untreated (endogenous Mglia) or received a GFP$^+$ Em-Mglia or GFP$^+$ Mo transfer. Slides stained with anti-IBA1 (red) and anti-P2RY12 (white). Scale bar, 50 μm. n = 2 untreated 3 mo, n = 3 Em-Mglia transfer 3 mo, and n = 3 Mo transfer 3 mo, from 2 independent experiment. **C)** *Cx3cr1*^{CreER}:*Csf1r*$^{fl/fl}$:5xFAD mice were treated with Tam at PD1-2 and transplanted with GFP$^+$ monocytes or Em-Mglia at PD3. Representative images of persistent engraftment of Em-Mglia (left panels) and Mo-Mglia (right panels) at 25 weeks post-transplantation. Top images: GFP (green), B-amyloid (white), and DAPI (blue). Bottom images: representative images of donor cells present in the cortex and hippocampus. GFP (green), B-amyloid (magenta), and DAPI (blue). n = 4 Em-Mglia transfer and n = 4 Mo transfer, from 2 independent experiments. Scale bar, 50 μm. **D)** Percentage of engraftment at 25 weeks post-transplantation of either GFP$^+$ Em-Mglia or Mo-Mglia in C. Significance was determined using an unpaired two-tailed *t*-test. Mean ± SD **E)** Coronal brain cryosections of 4-week-old *Cx3cr1*^{CreER}:*Csf1r*$^{fl/fl}$ that received a Mo-transfer as described in Figure 2A. Slides were stained with anti-IBA1 (red), anti-GFP (green), and anti-CLEC12A (white). Scale bar, 50 μm. Images representative of n = 2 mice, from 1 experiment. **F)** Flow cytometric gating strategy to identify Mo-Mglia engraftment in treated *Cx3cr1*^{CreER}:*Csf1r*$^{fl/fl}$ mice as shown in Figure 3A. *Cx3cr1*^{CreER}:*Csf1r*$^{fl/fl}$ mice were treated with various combinations of tamoxifen chow (C), Tamoxifen injection (I) and PLX3397 chow. All mice received an adoptive transfer of *Cx3cr1*$^{GFP/+}$ monocytes. Mice were sacrificed 4 weeks after adoptive transfers. Cells were pre-gated as CD45$^+$ live single cells. Cells were then gated as CD11b$^+$Ly6C$^-$CX3CR1$^+$F4/80$^+$. Em-Mglia were gated as GFP$^-$CLEC12A$^-$, adoptively transferred Mo-Mglia were gated as GFP$^+$CLEC12A$^+$, and endogenous Mo-Mglia were gated as GFP$^-$CLEC12A$^+$. Data representative of n = 7 (control), n = 7 (Tam C), n = 6 (Tam + CI), n = 5+7 (1 and 2w PLX + Tam CI, respectively), 10- to 11- weeks old male and female mice. Control and TamC: 2, TamCI: 3, and 1/2w PLX + TamCI: 4 independent experiments. **G)** Percentage of Mo-Mglia engraftment within experiment described in F. Left bar chart depicts adoptively transferred Mo-Mglia engraftment (GFP$^+$ CLEC12A$^+$), center bar chart depicts the endogenous Mo-Mglia engraftment (GFP$^-$ CLEC12A$^+$), and the right bar chart depicts the combined engraftment. Mean ± SD. Significances were determined using Tukey's multiple comparisons test. **H)** Comparison of the percentage of GFP$^+$ engraftment between 1W and 2W PLX + Tam CI groups described in Figure 3C and Figure S2G. Mean ± SD. Significances were determined using unpaired two-tailed *t*-tests. **I)** Morphology analysis of embryonic Mglia versus Mo-mglia. n = 5 10-11-week-old C57BL/6 male and female mice (embryonal Mglia) and n = 5 10-11-week-old *Cx3cr1*^{CreER}:*Csf1r*$^{fl/fl}$ male and female mice (Mo-Mglia) that received PLX + Tam CI treatment as described in Figure 3A. Data from 2 independent experiments. Mean ± SD. Significances were determined using unpaired two-tailed *t*-tests. **J)** Quantification of the normalized K(r) (observed value – theoretical value) for all cell detections across 50, 100, 200, and 400 μm distances for the PLX + Tam CI experiment (Figure 3F). Mean ± SD. Significances were determined using Dunnett's multiple comparisons test.

ns, *$p < 0.05$, **$p < 0.01$, ***$p < 0.001$, ****$p < 0.0001$

ctx = cortex, hipp = hippocampus, cc= corpus callosum, stria = striatum, thal = thalamus, brainst = brainstem.

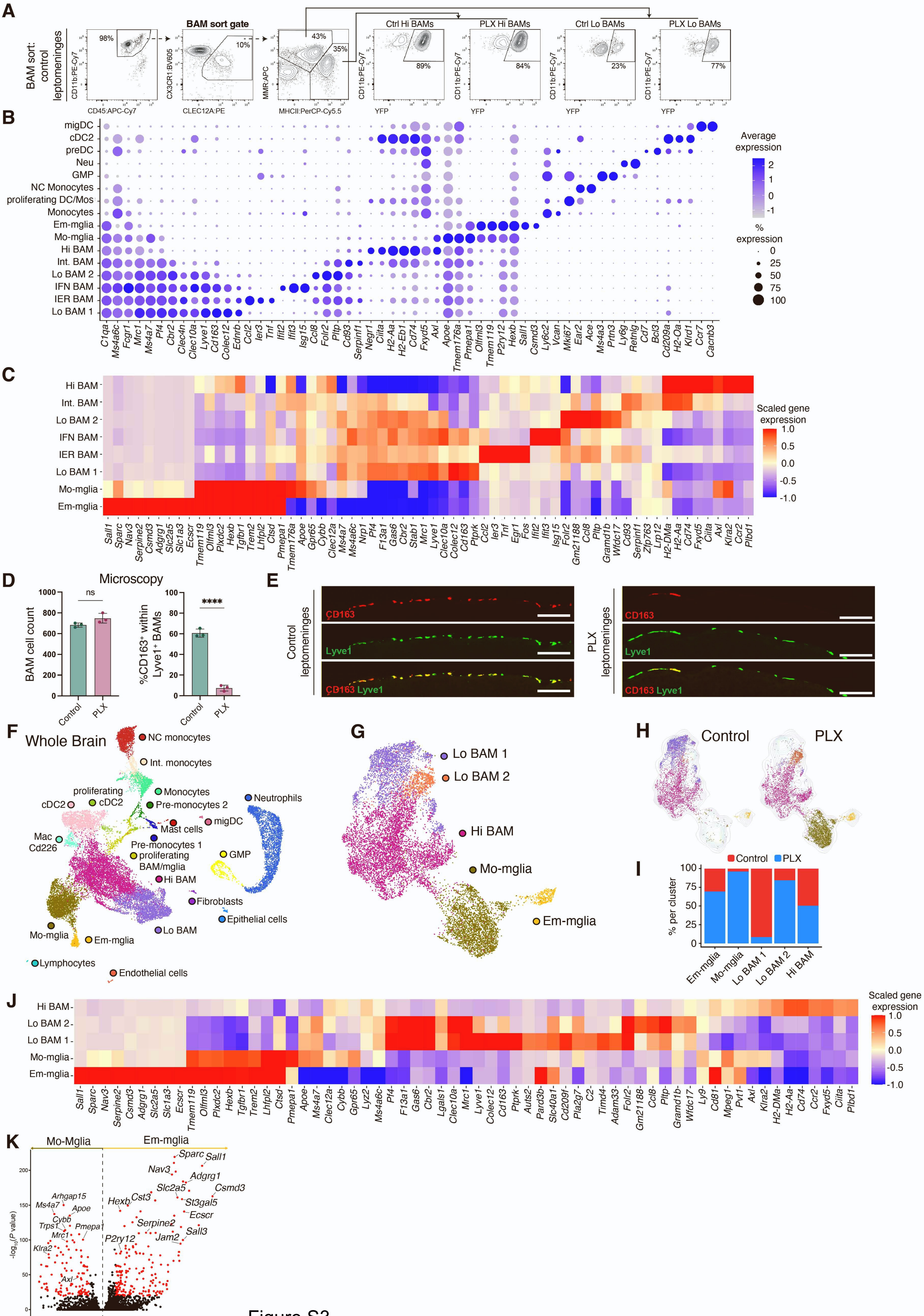
A
BAM sort: control leptomeninges
98%
CD11b:PE-Cy7
CD45:APC-Cy7
BAM sort gate
10%
CX3CR1:BV605
CLEC12A:PE
43%
35%
MMR:APC
MHCII:PerCP-Cy5.5
Ctrl Hi BAMs
89%
PLX Hi BAMs
84%
Ctrl Lo BAMs
23%
PLX Lo BAMs
77%
YFP
B
migDC
cDC2
preDC
Neu
GMP
NC Monocytes
proliferating DC/Mos
Monocytes
Em-mglia
Mo-mglia
Hi BAM
Int. BAM
Lo BAM 2
IFN BAM
IER BAM
Lo BAM 1
Average expression
% expression
C1qa Ms4a6c Fcgr1 Mrc1 Ms4a7 Pf4 Cbr2 Clec4n Clec10a Lyve1 Cd163 Colec12 Ednrb Ccl2 Ier3 Tnf Ifit2 Ifit3 Isg15 Ccl8 Folr2 Pltp Cd93 Serpinf1 Negr1 Ciita H2-Aa H2-Eb1 Cd74 Fxyd5 Axl Apoe Tmem176a Pmepa1 Olfml3 Tmem119 P2ry12 Hexb Sall1 Csmd3 Ly6c2 Vcan Mki67 Ear2 Ace Ms4a3 Prtn3 Ly6g Retnlg Cd7 Bcl3 Cd209a H2-Oa Klrd1 Ccr7 Cacnb3
C
Hi BAM
Int. BAM
Lo BAM 2
IFN BAM
IER BAM
Lo BAM 1
Mo-mglia
Em-mglia
Scaled gene expression
Sall1 Sparc Nav3 Serpine2 Csmd3 Adgrg1 Slc2a5 Slc1a3 Ecscr Tmem119 Olfml3 Plxdc2 Hexb Tgfbr1 Trem2 Lhfpl2 Ctsd Pmepa1 Tmem176a Apoe Gpr65 Cybb Clec12a Ms4a7 Ms4a6c Nrp1 Pf4 F13a1 Gas6 Cbr2 Stab1 Mrc1 Lyve1 Clec10a Colec12 Cd163 Ptprk Ccl2 Ier3 Tnf Egr1 Fos Ifit2 Ifit3 Isg15 Folr2 Gm21188 Ccl8 Pltp Gramd1b Wfdc17 Cd93 Serpinf1 Zfp763 Lrp12 H2-DMa H2-Aa Cd74 Fxyd5 Ciita Axl Klra2 Ccr2 Plbd1
D
Microscopy
ns
BAM cell count
****
%CD163+ within Lyve1+ BAMs
Control
PLX
E
Control leptomeninges
PLX leptomeninges
CD163
Lyve1
CD163 Lyve1
F
Whole Brain
NC monocytes
Int. monocytes
Monocytes
Pre-monocytes 2
proliferating cDC2
cDC2
Mac Cd226
Mast cells
migDC
Neutrophils
Pre-monocytes 1
proliferating BAM/mglia
GMP
Hi BAM
Fibroblasts
Epithelial cells
Lo BAM
Mo-mglia
Em-mglia
Lymphocytes
Endothelial cells
G
Lo BAM 1
Lo BAM 2
Hi BAM
Mo-mglia
Em-mglia
H
Control
PLX
I
Control
PLX
% per cluster
Em-mglia
Mo-mglia
Lo BAM 1
Lo BAM 2
Hi BAM
J
Hi BAM
Lo BAM 2
Lo BAM 1
Mo-mglia
Em-mglia
Scaled gene expression
Sall1 Sparc Nav3 Serpine2 Csmd3 Adgrg1 Slc2a5 Slc1a3 Ecscr Tmem119 Olfml3 Plxdc2 Hexb Tgfbr1 Trem2 Lhfpl2 Ctsd Pmepa1 Apoe Ms4a7 Clec12a Cybb Gpr65 Lyz2 Ms4a6c Pf4 F13a1 Gas6 Cbr2 Lgals1 Clec10a Mrc1 Lyve1 Colec12 Cd163 Ptprk Auts2 Pard3b Slc40a1 Cd209f Pla2g7 C2 Timd4 Adam33 Folr2 Gm21188 Ccl8 Pltp Gramd1b Wfdc17 Ly9 Cd81 Mpeg1 Pvt1 Axl Klra2 H2-DMa H2-Aa Cd74 Ccr2 Fxyd5 Ciita Plbd1
K
Mo-Mglia
Em-mglia
Sparc
Sall1
Nav3
Adgrg1
Csmd3
Slc2a5
St3gal5
Arhgap15
Hexb
Cst3
Ms4a7
Apoe
Ecscr
Cybb
Serpine2
Trps1
Pmepa1
Sall3
Mrc1
P2ry12
Jam2
Klra2
Axl
-log10(P value)
Log2(FC)

Figure S3

Figure S3: Transcriptional signature of leptomeningeal and whole brain Lo BAMs and Microglia in untreated and PLX3397-treated *Flt3*^{Cre}:*Yfp* mice, related to Figure 4.

A) Representative FACS gating strategy of sorting performed for the scRNA-seq in Figure 4A. Mice received control chow (Control) or 2 weeks PLX3397 chow followed by 7 weeks of repopulation on control chow (PLX & Repop). CLEC12A$^+$ cells were sorted from brain and enriched leptomeninges of n = 8 (Control) and n = 7 (PLX & Repop) *Flt3*^{Cre}*:Yfp* mice, from 1 experiment, using FACS sorting. Cells were pre-gated as CD45$^+$ live single cells. Sorted cells were gated as CD45$^+$CD11b$^+$CX3CR1$^+$CLEC12A$^+$ live single cells. Plots also show %YFP expression in Lo BAMs and Hi BAMs in control and depleted-repopulated brains. **B)** Dotplot depicting gene expression of clusters annotated in Figure 4B. **C)** Heatmap of the normalized and scaled expression of marker genes for the different macrophage subsets found in Figure 4B. **D)** Leptomeningeal BAM analysis of Lyve1 and CD163 expression in control and PLX groups. Coronal section regions analyzed correlate to leptomeninges under the skull cap. Mice were fed normal chow or PLX3397 chow for 1 week. Subsequently, they were given normal chow for 4 weeks after which they were sacrificed. n = 3 (control) and n = 3 (PLX), from 1 experiment. **E)** Representative images of mice in D. Slides stained with anti-CD163 (red), anti-Lyve1 (green), and anti F4/80 (not shown). Scale bar, 100 μm. **F)** UMAP plot of immune cells from both control and 'PLX & Repop' conditions of the whole brain. Experimental paradigm described in Figure 4A and in A. **G)** UMAP plot of reclustered macrophages retrieved from the whole brain dataset shown in F. **H)** UMAP plots depicting cells originating from the 'control' and 'PLX & Repop' conditions in B. **I)** Percentage of cells derived from 'control' or 'PLX & Repop' conditions per cluster in G. **J)** Heatmap of the normalized and scaled expression of marker genes for the different macrophage subsets found in G. **K)** Volcano plot comparing DE genes between Em-Mglia and Mo-Mglia found in G. (Log_2(FC)>0.1, -Log_{10}(adjusted *P* value)>20). *P* values were adjusted with the Bonferroni correction.

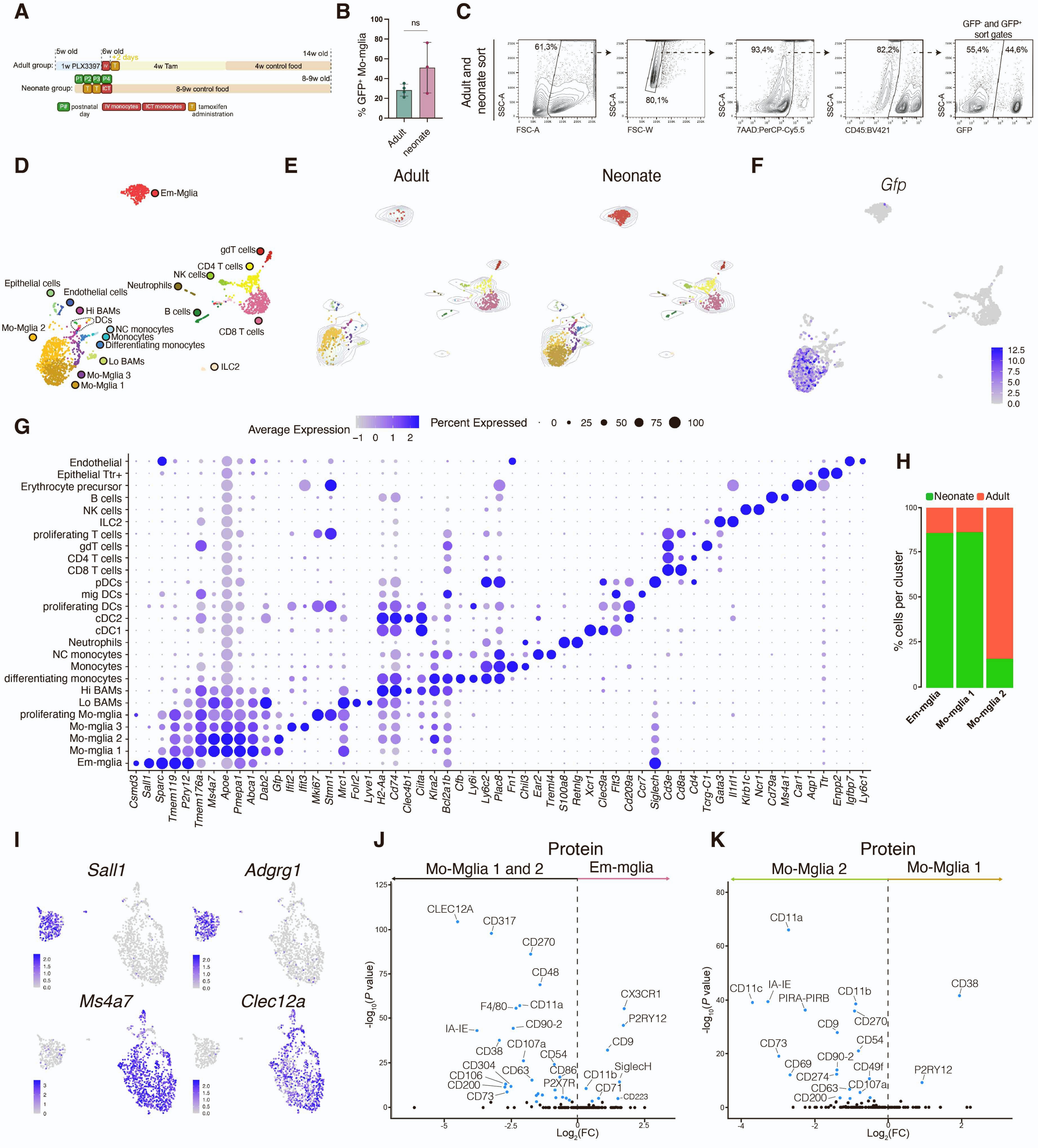
A
5w old
6w old
+2 days
14w old
Adult group:
1w PLX3397
4w Tam
4w control food
8-9w old
Neonate group:
8-9w control food
postnatal day
IV monocytes
ICT monocytes
tamoxifen administration
B
% GFP⁺ Mo-mglia
ns
Adult
neonate
C
Adult and neonate sort
GFP- and GFP+ sort gates
61,3%
80,1%
93,4%
82,2%
55,4%
44,6%
FSC-A
FSC-W
7AAD:PerCP-Cy5.5
CD45:BV421
GFP
SSC-A
D
Em-Mglia
gdT cells
CD4 T cells
NK cells
Epithelial cells
Endothelial cells
Neutrophils
Hi BAMs
B cells
DCs
Mo-Mglia 2
NC monocytes
Monocytes
Differentiating monocytes
CD8 T cells
Lo BAMs
ILC2
Mo-Mglia 3
Mo-Mglia 1
E
Adult
Neonate
F
Gfp
G
Average Expression
Percent Expressed
Endothelial
Epithelial Ttr+
Erythrocyte precursor
B cells
NK cells
ILC2
proliferating T cells
gdT cells
CD4 T cells
CD8 T cells
pDCs
mig DCs
proliferating DCs
cDC2
cDC1
Neutrophils
NC monocytes
Monocytes
differentiating monocytes
Hi BAMs
Lo BAMs
proliferating Mo-mglia
Mo-mglia 3
Mo-mglia 2
Mo-mglia 1
Em-mglia
Csmd3 Sall1 Sparc Tmem119 P2ry12 Tmem176a Ms4a7 Apoe Pmepa1 Abca1 Dab2 Gfp Ifit2 Ifit3 Mki67 Stmn1 Mrc1 Folr2 Lyve1 H2-Aa Cd74 Clec4b1 Ciita Klra2 Bcl2a1b Cfb Ly6i Ly6c2 Plac8 Fn1 Chil3 Ear2 Treml4 S100a8 Retnlg Xcr1 Clec9a Flt3 Cd209a Ccr7 Siglech Cd3e Cd8a Cd4 Tcrg-C1 Gata3 Il1rl1 Klrb1c Ncr1 Cd79a Ms4a1 Car1 Aqp1 Ttr Enpp2 Igfbp7 Ly6c1
H
Neonate
Adult
% cells per cluster
Em-mglia
Mo-Mglia 1
Mo-Mglia 2
I
Sall1
Adgrg1
Ms4a7
Clec12a
J
Protein
Mo-Mglia 1 and 2
Em-mglia
CLEC12A
CD317
CD270
CD48
F4/80
CD11a
CD90-2
IA-IE
CD107a
CD38
CD54
CD304
CD106
CD200
CD73
CD63
CD86
P2X7R
CX3CR1
P2RY12
CD9
SiglecH
CD11b
CD71
CD223
-log10(P value)
Log2(FC)
K
Protein
Mo-Mglia 2
Mo-Mglia 1
CD11a
CD11c
IA-IE
PIRA-PIRB
CD11b
CD270
CD9
CD38
CD73
CD54
CD90-2
CD69
CD274
CD49f
P2RY12
CD63
CD107a
CD200
-log10(P value)
Log2(FC)

Figure S4a

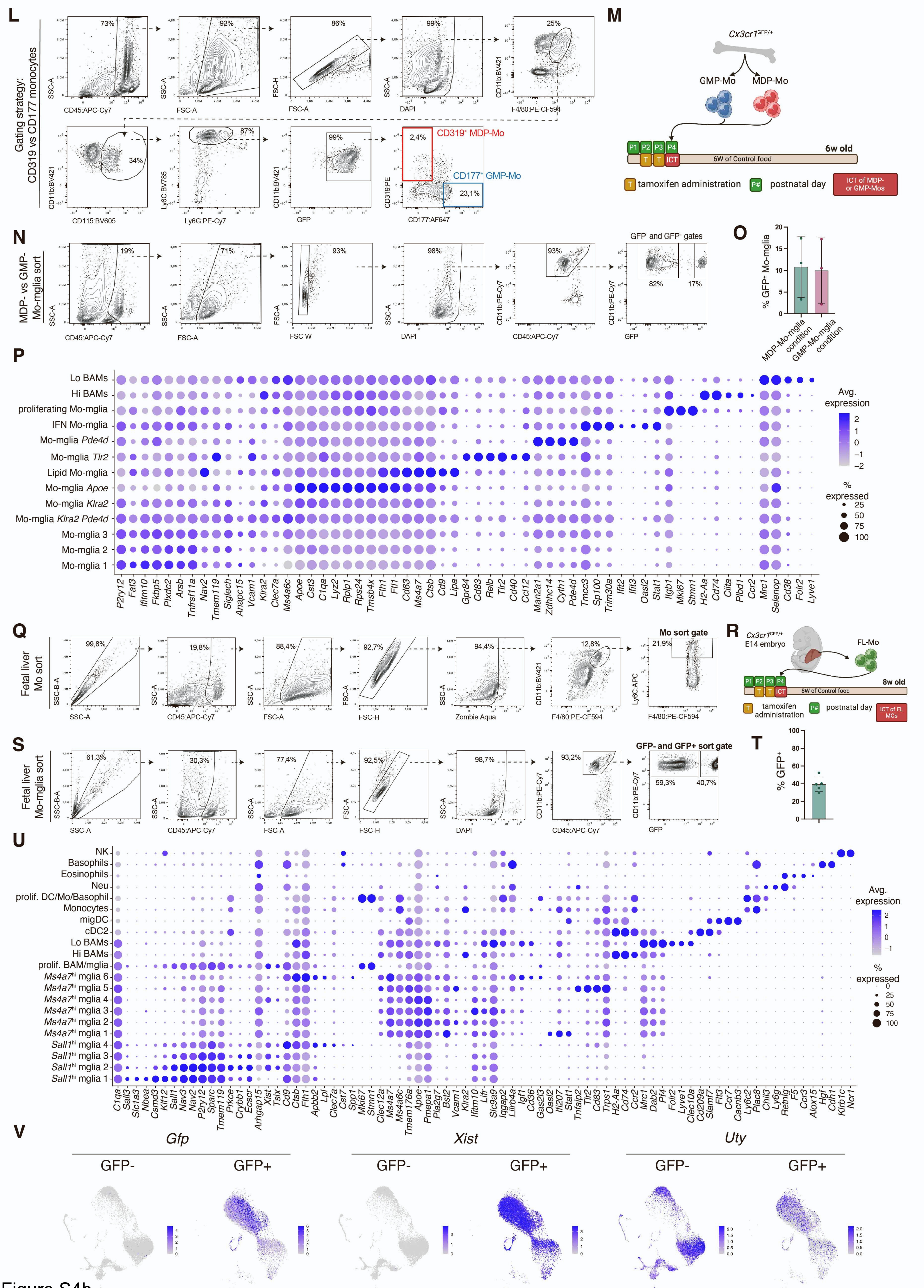

Gating strategy: CD319 vs CD177 monocytes
CD319⁺ MDP-Mo
CD177⁺ GMP-Mo
Cx3cr1GFP/+
GMP-Mo
MDP-Mo
6w old
6W of Control food
tamoxifen administration
postnatal day
ICT of MDP- or GMP-Mos
MDP- vs GMP- Mo-mglia sort
GFP- and GFP+ gates
% GFP+ Mo-mglia
MDP-Mo-mglia condition
GMP-Mo-mglia condition
Avg. expression
% expressed
Fetal liver Mo sort
Mo sort gate
Cx3cr1GFP/+ E14 embryo
FL-Mo
8w old
8W of Control food
ICT of FL MOs
Fetal liver Mo-mglia sort
GFP- and GFP+ sort gate
% GFP+
Gfp
Xist
Uty
GFP-
GFP+

Figure S4b

Figure S4: Bone marrow monocytes give rise to microglia which remain transcriptionally distinct from embryonal microglia while fetal liver monocytes generate bona fide microglia, related to Figure 4 and Figure 5.

A) Experimental layout for the CITE-seq in Figure 4I. Adult mice received the 1w PLX + Tam CI treatment and an adoptive transfer of *Cx3cr1*$^{GFP/+}$ monocytes. After 4 weeks on Tam chow, mice were switched to control chow for an additional 4 weeks before they were sacrificed. Neonates received intraperitoneal tamoxifen over 2 days at PD2 and PD3, and at PD 4 they were intracerebrally injected with *Cx3cr1*$^{GFP/+}$ monocytes. 8 weeks later, this group was sacrificed. Both conditions were processed for scRNA-seq on the same day. Brains were harvested from n = 4 (adult) 14-week-old males and n = 3 (neonate) 8-week-old females, from 1 experiment. CD45^{+}GFP^{+} and CD45^{+}GFP^{-} cells were FACS sorted from whole brains of both groups. GFP^{+} and GFP^{-} cells were combined in a 1:1 ratio after sorting. **B)** Percentage of CD45^{+}GFP^{+} cells in the brain per condition. Mean ± SD n = 4 (adult) n = 3 (neonate)**.** Significance was determined using an unpaired two-tailed *t*-tests. **C)** Representative FACS gating strategy for the sorted cells used for CITE-seq. **D)** UMAP plot of immune cells from both the neonate and adult conditions. **E)** UMAP plots depicting cells originating from either the adult or neonate conditions. **F)** UMAP plot showing the expression of *Gfp* in the dataset from D. **G)** Dotplot depicting gene expression of clusters annotated in D. **H)** Percentage of cells derived from adult or neonate conditions for the Em-Mglia, Mo-Mglia 1, and Mo-Mglia 2 clusters from Figure 4J. **I)** UMAP plots showing expression of the indicated genes in the dataset from Figure 4J. **J)** Volcano plot comparing DE proteins between Em-mglia and the combined 'Mo-Mglia 1 and Mo-Mglia 2' clusters found in Figure 4J. (Log_2(FC)>0.25, -Log_{10}(adjusted *P* value)>2). *P* values were adjusted with the Bonferroni correction. **K)** Volcano plot comparing DE proteins between Mo-Mglia 1 and Mo-Mglia 2 clusters found in Figure 4J. (Log_2(FC)>0.25, -Log_{10}(adjusted *P* value)>2). *P* values were adjusted with the Bonferroni correction. **L)** FACS gating strategy for sorting of GMP- and MDP-monocytes. **M)** Schematic of experiment layout. CD319^{+} (MDP-Mo) and CD177^{+} (GMP-Mo) bone marrow monocytes were FACS sorted from *Cx3cr1*$^{GFP/+}$ mice and intracerebrally injected in PD4 *Cx3cr1*^{CreER}:*Csf1r*$^{fl/fl}$ neonates that received tam-treatment at PD2 and PD3 (experimental paradigm similar to Figure 4I). Six weeks later, CD45^{+} CD11b^{+} GFP^{+} cells were FACS sorted from whole brains. Sorting strategy shown in N. Brains were harvested from n = 3 (MDP-Mo) and n = 3 (GMP-Mo) 6-week-old males and females, from 1 experiment. **N)** FACS gating strategy of GMP-Mo-Mglia and MDP-Mo-Mglia cells sorted for scRNA-seq analysis in M. **O)** Percentage of GFP^{+} engraftment within CD45^{+}CD11b^{+} cells in the mice described in M. n = 3 MDP-Mo-Mglia and n = 3 GMP-Mo-Mglia, Mean ± SD. **P)** Dotplot depicting gene expression for the clusters shown in Figure 5D. **Q)** FACS gating strategy for sorting of fetal liver monocytes used in Figure 5H. **R)** *Cx3cr1*$^{GFP/+}$ E14 fetal liver monocytes were FACS sorted and intracerebrally injected in PD4 *Cx3cr1*^{CreER}:*Csf1r*$^{fl/fl}$ neonates that received tam-treatment at PD2 and PD3 (experiment similar to Figure 4I). 8 weeks later CD45^{+}CD11b^{+}GFP^{+} and GFP^{-} cells were FACS sorted from whole brains. GFP^{+} and GFP^{-} cells were individually processed after FACS sorting and later combined *in-silico*. Brain myeloid cell sorting strategy available in S. Brains harvested from n = 5 males. **S)** FACS gating strategy of Fetal liver Mo-Mglia cells sorted for scRNA-seq analysis in R. **T)** Percentage of CD45^{+}CD11b^{+}GFP^{+} engraftment in the mice described in R,S and Figure 5H. n = 5, Mean ± SD. **U)** Dotplot depicting gene expression for the clusters annotated in Figure 5I. **V)** UMAP plots showing expression of selected genes split per condition in the dataset shown in Figure 5I.

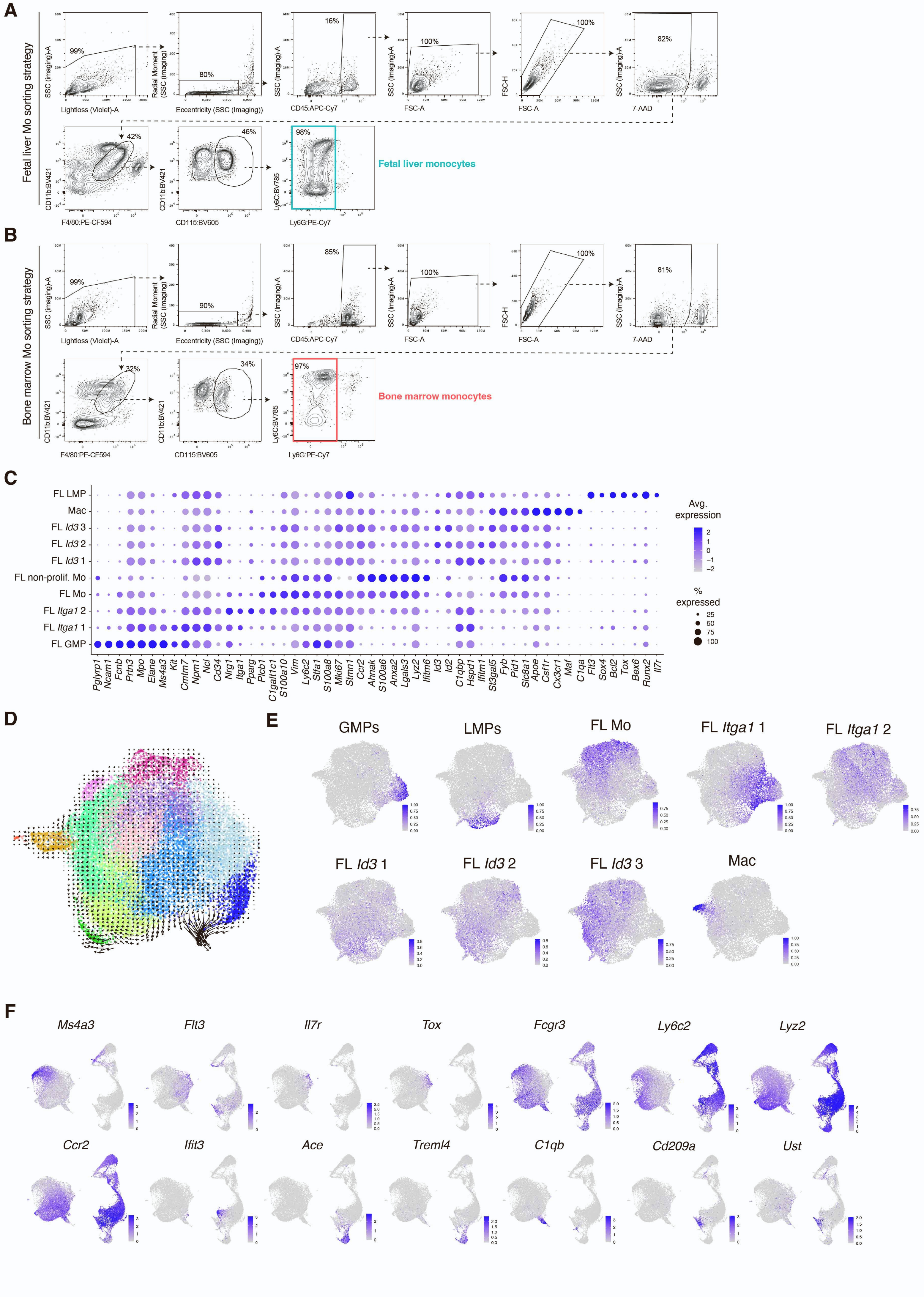

A
Fetal liver Mo sorting strategy
Lightloss (Violet)-A
SSC (imaging)-A
Radial Moment (SSC (imaging))
Eccentricity (SSC (Imaging))
CD45:APC-Cy7
FSC-A
FSC-H
7-AAD
99%
80%
16%
100%
100%
82%
CD11b:BV421
F4/80:PE-CF594
CD115:BV605
Ly6C:BV785
Ly6G:PE-Cy7
42%
46%
98%
Fetal liver monocytes
B
Bone marrow Mo sorting strategy
99%
90%
85%
100%
100%
81%
32%
34%
97%
Bone marrow monocytes
C
FL LMP
Mac
FL Id3 3
FL Id3 2
FL Id3 1
FL non-prolif. Mo
FL Mo
FL Itga1 2
FL Itga1 1
FL GMP
Pglyrp1
Ncam1
Fcnb
Prtn3
Mpo
Elane
Ms4a3
Kit
Cmtm7
Npm1
Ncl
Cd34
Nrg1
Itga1
Pparg
Plcb1
C1galt1c1
S100a10
Vim
Ly6c2
Stfa1
S100a8
Mki67
Stmn1
Ccr2
Ahnak
S100a6
Anxa2
Lgals3
Lyz2
Ifitm6
Id3
Id2
C1qbp
Hspd1
Ifitm1
St3gal5
Fyb
Pid1
Slc8a1
Apoe
Csf1r
Cx3cr1
Maf
C1qa
Flt3
Sox4
Bcl2
Tox
Bex6
Runx2
Il7r
Avg. expression
% expressed
D
E
GMPs
LMPs
FL Mo
FL Itga1 1
FL Itga1 2
FL Id3 1
FL Id3 2
FL Id3 3
Mac
F
Ms4a3
Flt3
Il7r
Tox
Fcgr3
Ly6c2
Lyz2
Ccr2
Ifit3
Ace
Treml4
C1qb
Cd209a
Ust

Figure S5

Figure S5. Fetal liver monocyte and bone marrow monocyte FACS sorting strategies, related to Figure 6.

A) FACS gating strategy for sorting of bone marrow monocytes. **B)** FACS gating strategy for sorting of fetal liver monocytes. **C)** Dotplot depicting gene expression for the FL clusters shown in Figure 6B. **D)** RNA velocity plot of the FL scRNAseq dataset from Figure 6B. **E)** UMAP plots showing prediction scores for each FL Mo scRNA-seq cluster from Figure 6B calculated for each cells of the snATAC-seq dataset from Figure 6D. **F)** UMAP plots showing expression of selected genes in the dataset from Figure 6F.

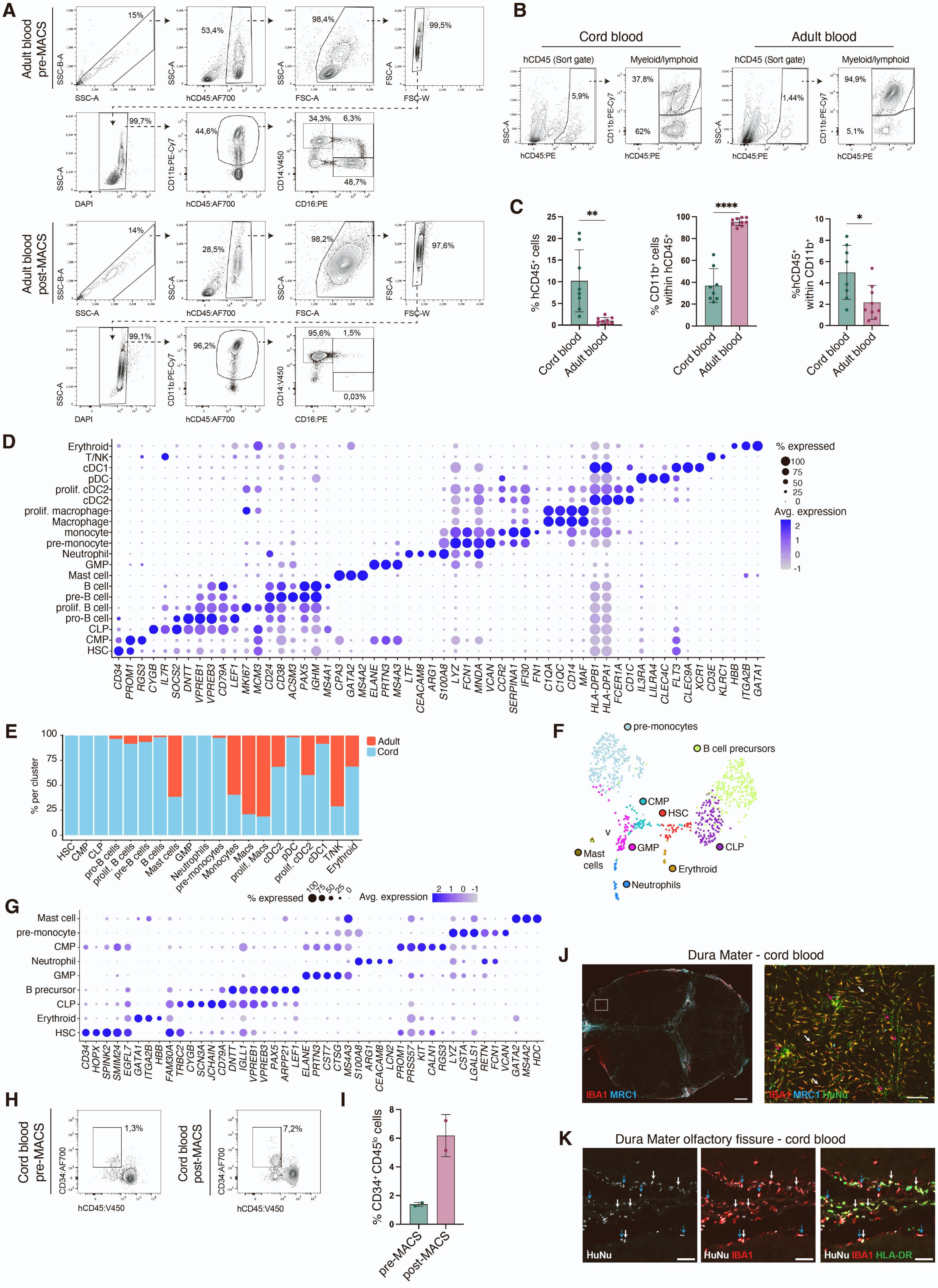
A
Adult blood pre-MACS
Adult blood post-MACS
SSC-B-A
SSC-A
FSC-A
FSC-W
hCD45:AF700
DAPI
CD11b:PE-Cy7
CD14:V450
CD16:PE
B
Cord blood
Adult blood
hCD45 (Sort gate)
Myeloid/lymphoid
hCD45:PE
CD11b:PE-Cy7
C
% hCD45+ cells
% CD11b+ cells within hCD45+
%hCD45+ within CD11b+
Cord blood
Adult blood
D
% expressed
Avg. expression
Erythroid
T/NK
cDC1
pDC
prolif. cDC2
cDC2
prolif. macrophage
Macrophage
monocyte
pre-monocyte
Neutrophil
GMP
Mast cell
B cell
pre-B cell
prolif. B cell
pro-B cell
CLP
CMP
HSC
CD34 PROM1 RGS3 CYGB IL7R SOCS2 DNTT VPREB1 VPREB3 CD79A LEF1 MKI67 MCM3 CD24 CD38 ACSM3 PAX5 IGHM MS4A1 CPA3 GATA2 MS4A2 ELANE PRTN3 MS4A3 LTF CEACAM8 ARG1 S100A8 LYZ FCN1 MNDA VCAN CCR2 SERPINA1 IFI30 FN1 C1QA C1QC CD14 MAF HLA-DPB1 HLA-DPA1 FCER1A CD1C IL3RA LILRA4 CLEC4C FLT3 CLEC9A XCR1 CD3E KLRC1 HBB ITGA2B GATA1
E
% per cluster
Adult
Cord
HSC CMP CLP pro-B cells prolif. B cells pre-B cells B cells Mast cells GMP Neutrophils pre-monocytes Monocytes Macs prolif. Macs cDC2 pDC prolif. cDC2 cDC1 T/NK Erythroid
F
pre-monocytes
B cell precursors
CMP
HSC
GMP
CLP
Mast cells
Erythroid
Neutrophils
G
% expressed
Avg. expression
Mast cell
pre-monocyte
CMP
Neutrophil
GMP
B precursor
CLP
Erythroid
HSC
CD34 HOPX SPINK2 SMIM24 EGFL7 GATA1 ITGA2B HBB FAM30A TRBC2 CYGB SCN3A JCHAIN CD79A DNTT IGLL1 VPREB1 VPREB3 PAX5 ARPP21 LEF1 ELANE PRTN3 CST7 CTSG MS4A3 S100A8 ARG1 CEACAM8 LCN2 PROM1 PRSS57 KIT CALN1 RGS3 LYZ CSTA LGALS1 RETN FCN1 VCAN GATA2 MS4A2 HDC
H
Cord blood pre-MACS
Cord blood post-MACS
CD34:AF700
hCD45:V450
I
% CD34+ CD45lo cells
pre-MACS
post-MACS
J
Dura Mater - cord blood
IBA1 MRC1
IBA1 MRC1 HuNu
K
Dura Mater olfactory fissure - cord blood
HuNu
HuNu IBA1
HuNu IBA1 HLA-DR

Figure S6a

L

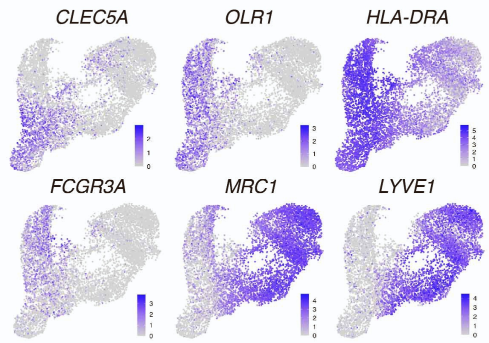

M

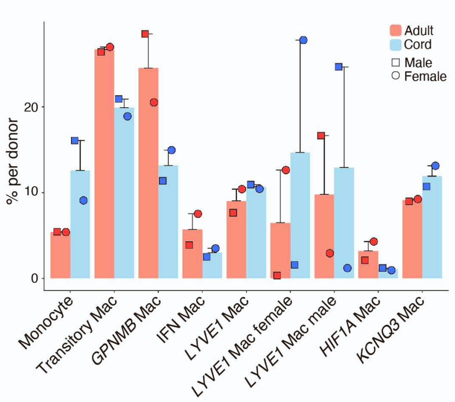

N

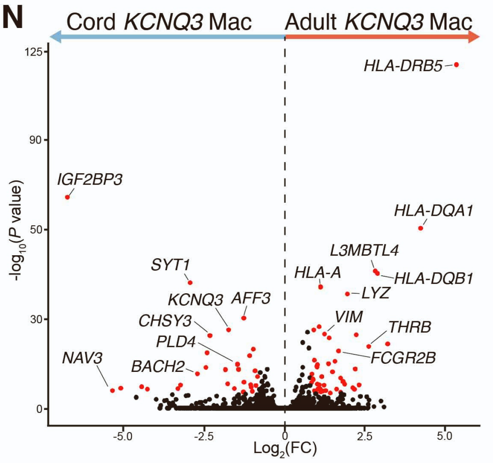

O

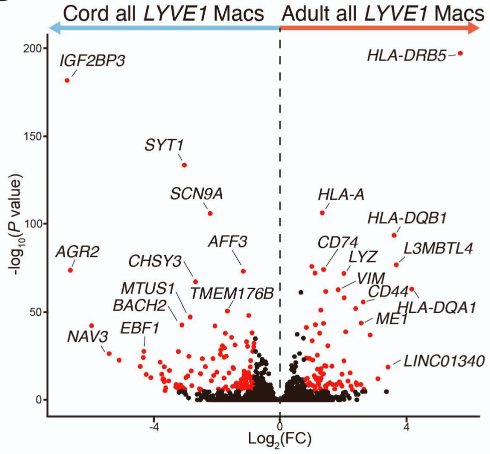

P

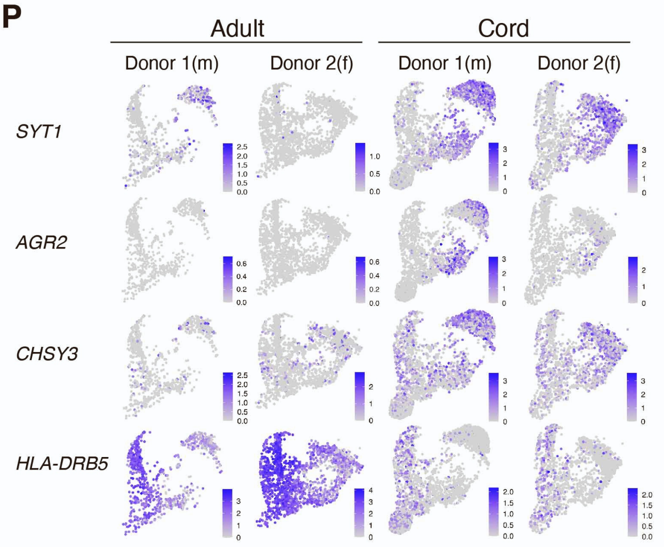

Q

Cord *KCNQ3* Mac:

GO:0050804: modulation of chemical synaptic transmission
SYT1, PDE9A, KCNQ3, CYFIP1
GO:0006897: endocytosis
SYT1, KCNQ3, PLD4, ARHGAP12
GO:0071277: cellular response to calcium ion
MELTF, SYT1, SCN9A, PLEKHA1

Adult *KCNQ3* Mac:

GO:0048002: antigen processing and presentation
HLA-DRB5, CD300E, FCGRA3, CFD
R-HSA-2172127: DAP12 interactions
CD300E, LYZ, CLEC5A, MGAM
GO:0006954: inflammatory response
LYZ, CD44, IER3, TIMP1

R

Cord all *LYVE1* Macs:

GO:0002764: immune response-regulating signaling pathway
IGHM, CD79B, IGLL1, IGLC1, CD24, BACH2
GO:1903034: regulation of response to wounding
AGR2, SPP1, IGLL1, CD24, VPREB1, GNG11
GO:0098900: regulation of action potential
RAMP1, SYT1, KCNQ3, FCMR, MYO1D

Adult all *LYVE1* Macs:

GO:0019882 antigen processing and presentation
HLA-DRB5, CD44, CLEC10A, VIM, PTPN22
R-HSA-6798695 Neutrophil degranulation
RNASE2, LYZ, HK3, CLEC5A, CLEC10A, VIM, ITGAX
GO:0001819 positive regulation of cytokine production
LURAP1, PTGER4, CD151, CLEC4E, CD300LF, CD74

Figure S6b

Figure S6. Human cord and adult blood monocytes engraft as BAMs and microglia-like cells in the mouse brain, related to Figure 7.

A) Flow cytometry plots revealing $CD14^+$ classical monocyte purity before and after MACS purification from adult blood. Classical monocytes are gated as $CD45^+CD11b^+CD14^+CD16^-$ **B)** Representative sorting gates for scRNA-seq experiment described in Figure 7A. Cells were pre-gated as live single cells. **C)** Left bar chart: percentage of $hCD45^+$ engraftment within the hCSF1 mouse brains. Center bar chart: percentage of $CD11b^+$ cells within $hCD45^+$ fraction. Right bar chart: percentage of $hCD45^+$ cells within total $CD11b^+$ fraction. Charts related to scRNA-seq experiment described in Figure 7A. Significances were determined using unpaired two-tailed *t*-tests. **D)** Dotplot depicting gene expression of the annotated clusters in Figure 7B. **E)** Proportion of cells derived from cord or adult blood conditions per cluster in Figure 7B. **F)** UMAP of reclustered progenitor cells from Figure 7B. CMP (common myeloid progenitor), GMP (granulocyte/monocyte progenitor), CLP (common lymphoid progenior) **G)** Dotplot depicting gene expression of the annotated clusters in F. **H)** Flow cytometry plots revealing the percentage of $CD34^+$ cells within live single cells from cord blood before and after MACS purification. Cells were pre-gated as live, single cells. n=2, from 1 experiment. **I)** Percentage of $CD34^+hCD45^{lo}$ cells from cord blood before and after MACS purification in H. n =2, from 1 experiment. Mean ± SD. **J)** Meninges whole mount of hCSF1:$Rag2^{-/-}$:$\gamma c^{-/-}$ mice that received the cord blood intracerebral injection. Scale bar, 1 mm. Right image: inset demonstrating $IBA1^+HuNu^+MRC1^-$ cells (white arrows) and small circular $IBA1^-HuNu^+MRC1^-$ cells (dashed magenta arrows). Scale bar, 50 μm. Representative images of n = 3 mice, from 1 experiment. **K)** Coronal brain sections stained with anti-IBA1 (red), HLA-DR (green), and anti-human nucleus (white). $IBA1^+HuNu^+HLA\text{-}DR^+$ cells (white arrows) and $IBA1^+HuNu^+HLA\text{-}DR^-$ cells (dashed blue arrows). Scale bars, 50 μm. Representative images of n = 3 mice, from one experiment. **L)** UMAP plot showing the expression of selected genes in the dataset shown in Figure 7F. **M)** Percentage of cells derived from cord blood or adult blood conditions for the cluster annotated in Figure 7F. Datapoints are further split up into male and female donors per condition per cluster. n = 2, Mean ± SD **N)** Volcano plot comparing DE genes of cells originating from the adult versus cord blood condition, specifically for the *KCNQ3* Mac cluster found in Figure 7F. (Log_2(FC)>0.8, -Log_{10}(adjusted *P* value)>5). *P* values were adjusted with the Bonferroni correction. **O)** Volcano plot comparing DE genes of the combined *LYVE1* Mac clusters (*LYVE1* Mac, *LYVE1* Mac (female), and *LYVE1* Mac (male)) between the conditions 'cord blood' and 'adult blood' found in Figure 7F. (Log_2(FC)>0.8, -Log_{10}(adjusted *P* value)>5). *P* values were adjusted with the Bonferroni correction. **P)** UMAP plots depicting expression of the indicated genes, split per condition and per donor (m= male, f= female) for the dataset from Figure 7F. **Q)** Chosen terms retrieved from a gene ontology enrichment analysis on the DE genes from N. **R)** Chosen terms retrieved from a gene ontology enrichment analysis on the DE genes from O.

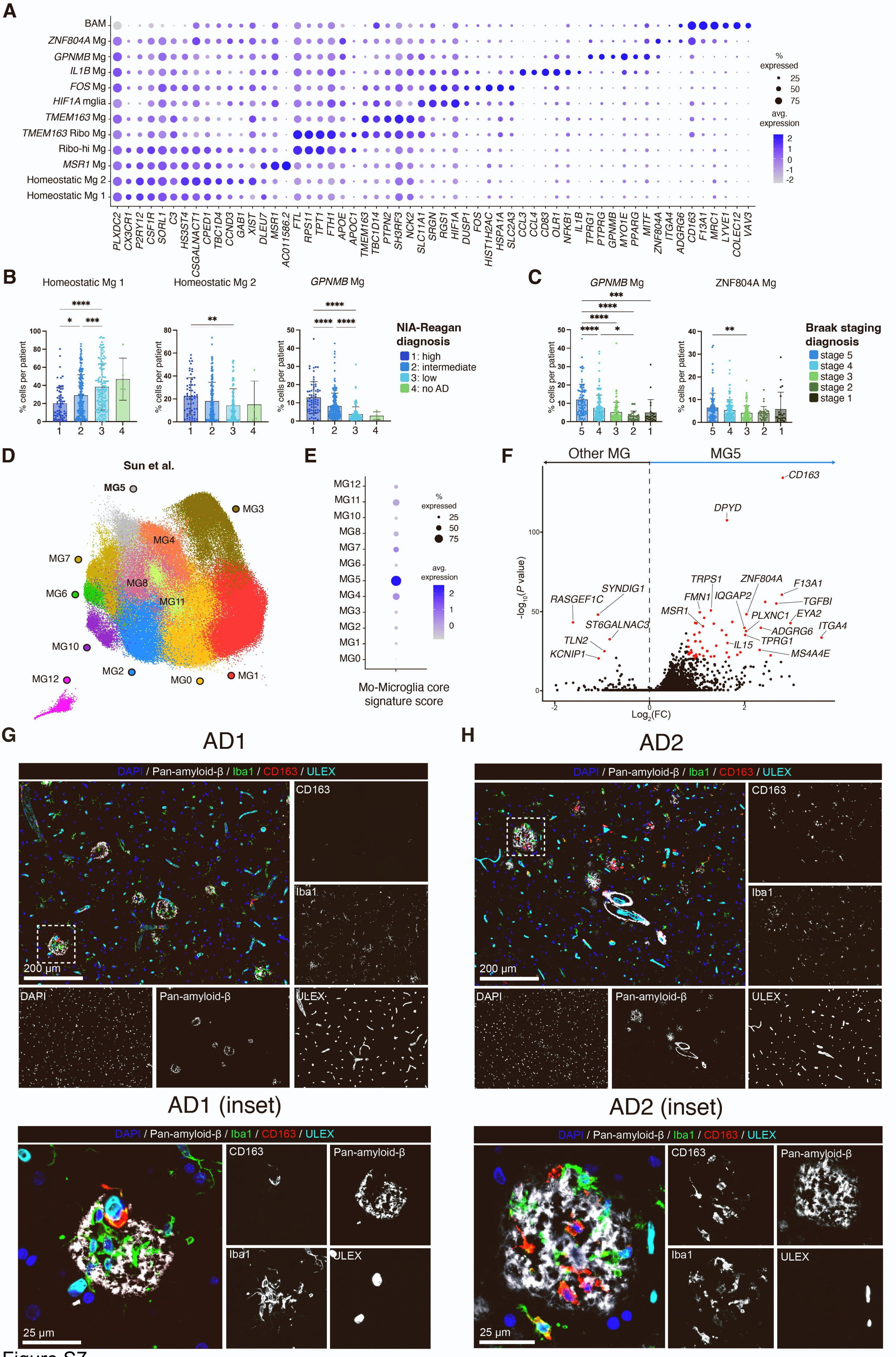
A
BAM
ZNF804A Mg
GPNMB Mg
IL1B Mg
FOS Mg
HIF1A mglia
TMEM163 Mg
TMEM163 Ribo Mg
Ribo-hi Mg
MSR1 Mg
Homeostatic Mg 2
Homeostatic Mg 1
% expressed
avg. expression
B
Homeostatic Mg 1
Homeostatic Mg 2
GPNMB Mg
% cells per patient
NIA-Reagan diagnosis
1: high
2: intermediate
3: low
4: no AD
C
GPNMB Mg
ZNF804A Mg
Braak staging diagnosis
stage 5
stage 4
stage 3
stage 2
stage 1
D
Sun et al.
E
Mo-Microglia core signature score
F
Other MG
MG5
-log10(P value)
Log2(FC)
G
AD1
DAPI / Pan-amyloid-β / Iba1 / CD163 / ULEX
200 µm
AD1 (inset)
25 µm
H
AD2
AD2 (inset)

Figure S7

Figure S7: Microglial identity and abundance in human Alzheimer's disease brains, related to Figure 7.

A) Dotplot depicting gene expression of the annotated clusters in Figure 7H. **B)** Relative abundance per cluster shown in Figure 7H in donors that were stratified based on the NIA-Reagan diagnosis score. Donors stratified per score: n= 68 'score 1', n = 198 'score 2', n = 153 'score 3', and n = 5 'score 4'. Significances were determined using Tukey's multiple comparisons test where column 4 was omitted due to lack of enough datapoints to perform relevant statistical tests. ns, *p < 0.05, **p < 0.01, ***p < 0.001, ****p < 0.0001 **C)** Relative abundance per cluster shown in Figure 7H in donors that were stratified based on the Braak staging diagnosis score. Staging scores 6 and 0 were omitted due to lack of sufficient data points. Donors stratified per score: n= 3 'score 6', n = 91 'score 5', n = 143 'score 4', and n = 124 'score 3', n = 32 'score 2', n = 24 'score 1', and n = 7 'score 0'. Significances were determined using Tukey's multiple comparisons. ns, *p < 0.05, **p < 0.01, ***p < 0.001, ****p < 0.0001 **D)** UMAP plot of 152 459 microglia nuclei of postmortem brain samples from 217 AD and 226 control individuals from the ROSMAP cohort of Sun et al. based on the original analysis of the authors. **E)** Dotplot of a 'Mo-Microglia core signature score' given to all the cells in D. The score shows the enrichment for genes, that are upregulated in *ZNF804A* Mg compared to the remaining cells in the ROSMAP snRNAseq dataset shown in Figure 7H (p_val_adj<0.05 and log2FC>0.8) **F)** Volcano plot comparing DE genes of the MG5 cluster against all other Mg found in D. (Log_2(FC)>0.8, -Log_{10}(adjusted *P* value)>20). *P* values were adjusted with the Bonferroni correction. **G, H)** Representative images of human frontal cortex of Alzheimer's disease patients (AD1 and AD2, respectively) stained with Iba1 (green), CD163 (red), pan-amyloid (white), ULEX (cyan), DAPI (blue). Images vessel associated $Iba1^{+}CD163^{+}$ cell in patient AD1; and reveal many non-vessel associated $Iba1^{+}CD163^{+}$ cells in patient AD2.

Table S1: antibodies for flow cytometry and FACS, related to STAR methods.

Antigen	Fluorophore	Reactivity	Clone	RRID	ID	Manufacturer	Dilution
c-kit	PE	mouse	2B8	AB_313217	105808	BioLegend	1/1000
CD11b	PE-Cy7	mouse/ human	M1/70	AB_312799	101216	BioLegend	1/1000
CD11b	BV421	mouse/ human	M1/70	AB_2562904	101251	BioLegend	1/1000
CD11c	BV605	mouse	N418	AB_2562415	117334	BioLegend	1/1000
CD14	V450	human	M5E2	AB_10611582	561390	BD Biosciences	1/1000
CD14	BV421	human	M5E2	AB_2739154	565283	BD Biosciences	1/1000
CD16	PE	human	3G8	AB_314208	302008	BioLegend	1/1000
CD24	AF700	mouse	M1/69	AB_2566730	101836	BioLegend	1/1000
CD34	AF700	human	561	AB_2632722	343621	BioLegend	1/1000
CD45	PE	human	HI30	AB_314396	304008	BioLegend	1/1000
CD45	AF700	human	HI30	AB_493761	304024	Biolegend	1/1000
CD45	BV421	mouse	30-F11	AB_2562559	103134	BioLegend	1/1000
CD45	APC-Cy7	mouse	30-F11	AB_312981	103116	BioLegend	1/1000
CD45	V450	human	HI30	AB_1645573	560367	BD Biosciences	1/1000
CD115	BV605	mouse	AFS98	AB_2562760	135517	BioLegend	1/1000
CD177	AF647	Mouse	Y127		566599	BD Biosciences	1/1000
CD319	PE	Mouse	4G2	AB_2632676	152005	BioLegend	1/1000
CLEC12A	PE	mouse	5D3/CLEC12A	AB_11126747	143403	BioLegend	1/1000
CX3CR1	BV605	mouse	SA011F11	AB_2565937	149027	BioLegend	1/500
F4/80	PE-CF594	mouse	T45-2342	AB_2734770	565613	BD Biosciences	1/500
FOLR2	APC	mouse	10/FR2	AB_2721313	153306	BioLegend	1/1000
I-A/I-E	PerCP-Cy5.5	mouse	M5/114.15.2	AB_2191071	107626	BioLegend	1/1000
Ly6C	APC	mouse	HK1.4	AB_1732076	128016	BioLegend	1/1000
Ly6C	BV510	mouse	HK1.4	AB_2562351	128033	BioLegend	1/1000
Ly6C	BV785	Mouse	HK1.4	AB_2565852	128041	BioLegend	1/1000
Ly6G	APC	mouse	1A8	AB_2227348	127613	BioLegend	1/1000
Ly6G	PE-Cy7	Mouse	1A8	AB_1877262	127617	BioLegend	1/1000
MMR	APC	mouse	C068C2	AB_10896057	141707	BioLegend	1/1000
MMR	AF700	mouse	C068C2	AB_2629637	141734	BioLegend	1/1000
SiglecF	AF700	mouse	1RNM44N	AB_2637126	56-1702-82	Invitrogen	1/1000

Table S2: antibodies for IHC, related to STAR methods.

Antigen	ID	Poly/mono	RRID	Manufacturer	Dilution
Chicken anti-GFP	Ab13970	Polyclonal	AB_300798	Abcam	1/500
Rat anti-CLEC12A	143402	5D3/CLEC12A	AB_11125370	BioLegend	1/100
Rabbit anti-IBA1	019-19741	Polyclonal	AB_839504	Wako	1/250
Goat anti-IBA1	Ab48004	Polyclonal	AB_870576	Abcam	1/250
Goat anti-Iba1	Ab5076	Polyclonal	AB_2224402	Abcam	1/250 (1/100

					Figure S12)
Rabbit anti-RFP	Ab62341	Polyclonal	AB_945213	Abcam	1/250
Rat anti-Lyve1	14-0443-82	ALY7	AB_1633414	eBioscience	1/100
Goat anti-MMR	AF2535	Polyclonal	AB_2063012	R&D systems	1/100
Mouse anti-human nucleus	MAB1281	235-1	AB_94090	Chemicon	1/200
Rabbit anti-P2RY12	AS-55043A	Polyclonal	AB_2298886	Anaspec	1/500
Rabbit anti-B-amyloid	8243S	D52D2	AB_2797642	Cell Signaling Technology	1/1000 (1/100 Figure S12)
Rat anti-CD31	550274	MEC 13.3	AB_394815	BD Biosciences	1/100
Rabbit anti-CD163	Ab182422	EPR19518	AB_2753196	Abcam	1/200
Rat anti-F4/80	MCA497GA	A3-1	AB_323806	Bio-Rad	1/250
Goat anti-Lyve1	AF2125	polyclonal	AB_2297188	R&D Systems	1/200
Human anti-human HLA-DR	130-122-299	REA805	AB_2801880	Miltenyi	1/50
Streptavidin-Alexa Fluor 750	S21384	/	/	Invitrogen	1/400
Ulex-biotin	B-1065-2	/	/	Vector Laboratories	1/200
Donkey anti-rabbit Alexa Fluor 647	A31573	Polyclonal	AB_2536183	Invitrogen	1/500 (1/400 Figure S12)
Donkey anti-goat Alexa Fluor 647	A21447	Polyclonal	AB_141844	Invitrogen	1/500
Donkey anti-goat Alexa Fluor 647	A32849	Polyclonal	AB_2762840	Invitrogen	1/500
Donkey anti-goat Alexa Fluor 568	A11057	Polyclonal	AB_142581	Invitrogen	1/500
Goat anti-rat Alexa Fluor 555	A21434	Polyclonal	AB_141733	Invitrogen	1/500
Donkey anti-rabbit Alexa Fluor 546	A10040	Polyclonal	AB_2534016	Invitrogen	1/500
Donkey anti-Rabbit 594	A21207	Polyclonal	AB_141637	Invitrogen	1/500
Donkey anti-rat Alexa Fluor 555 plus	A48270	Polyclonal	AB_2896336	Invitrogen	1/1000
Donkey anti-mouse Alexa Fluor 488	A21202	Polyclonal	AB_141607	Invitrogen	1/500
Donkey anti-chicken CF488A	SAB4600031	Polyclonal	AB_2721061	Merck	1/500
Goat anti-chicken Alexa Fluor 488	A11039	Polyclonal	AB_142924	Invitrogen	1/500
Donkey anti-Rabbit Alexa Fluor 488	A21206	Polyclonal	AB_2535792	Invitrogen	1/500
Donkey anti-goat Alexa Fluor 488	A11055	Polyclonal	AB_2534102	Invitrogen	1/500
Donkey anti-human DyLight 488	SA5-10126	Polyclonal	AB_2556706	Invitrogen	1/500
Donkey anti-goat Alexa Fluor 594	A11058	Polyclonal	AB_2534105	Invitrogen	1/400
Rabbit anti-B-amyloid-Alexa Fluor 488	51374S	D52D2	AB_2799392	Cell Signaling Technology	1/100

Table S4: mice used, related to STAR methods.

Figure	Strain	Group	age	n	Sex (M/F)
1A, S1A	*Flt3*^{Cre}:*Yfp*	Control	14w	6	M
	Flt3^{Cre}:*Yfp*	PLX	14w	7	M
1B	*Flt3*^{Cre}:*Yfp*	Control	5mo	4	M
	Flt3^{Cre}:*Yfp*	PLX	5mo	6	M
1C	*Flt3*^{Cre}:*Yfp*	PLX	10W	3	M
S1C,D	C57BL/6	PLX + Mo transfer	13W	4	M
1D,E,H, S1E,G	*Flt3*^{Cre}:*Yfp*	PLX	10-16W	5	M
	Flt3^{Cre}:*Yfp*	PLX+200RAD	10-16W	6	M
	Flt3^{Cre}:*Yfp*	PLX+400RAD	10-16W	7	M
	Flt3^{Cre}:*Yfp*	PLX+600RAD	10-16W	6	M
	Flt3^{Cre}:*Yfp*	PLX+800RAD	10-16W	6	M
	Flt3^{Cre}:*Yfp*	Naïve blood	4-6W	4	M
1F,G, S1F	*Flt3*^{Cre}:*Yfp*	PLX	10W	5	M
	Flt3^{Cre}:*Yfp*	PLX+600RAD	10W	5	M
1I,J	C57BL/6	Control	10W	3	1M/2F
	C57BL/6	1W PLX	10W	6	3M/3F
	C57BL/6	2W PLX	11W	6	3M/3F
1K,L	C57BL/6	Control	10W	3	1M/2F
	C57BL/6	1W PLX	10W	6	2M/4F
	C57BL/6	2W PLX	11W	6	2M/4F
2A, S2A	*Cx3cr1*^{CreER}:*Csf1r*$^{fl/fl}$	No tam	PD3	3	unknown
	Cx3cr1^{CreER}:*Csf1r*$^{fl/fl}$	Tam	PD3	2	unknown
	Cx3cr1^{CreER}:*Csf1r*$^{fl/fl}$	Mo transfer	PD3	2	unknown
	Cx3cr1^{CreER}:*Csf1r*$^{fl/fl}$	No tam	PD7	3	1M/2F
	Cx3cr1^{CreER}:*Csf1r*$^{fl/fl}$	Tam	PD7	3	1M/2F
	Cx3cr1^{CreER}:*Csf1r*$^{fl/fl}$	Mo transfer	PD7	3	unknown
	Cx3cr1^{CreER}:*Csf1r*$^{fl/fl}$	No Tam	PD16	4	1M/3F
	Cx3cr1^{CreER}:*Csf1r*$^{fl/fl}$	Tam	PD16	3	2F/1 unknown
	Cx3cr1^{CreER}:*Csf1r*$^{fl/fl}$	Mo transfer	PD16	8	3M/3F (2 unknown)
2B,C	*Cx3cr1*^{CreER}:*Csf1r*$^{fl/fl}$	Mglia transfer	PD16	9	4M/5F
	Cx3cr1^{CreER}:*Csf1r*$^{fl/fl}$	Mo transfer	PD16	6	3M/3F
	Cx3cr1^{CreER}:*Csf1r*$^{fl/fl}$	Mglia transfer	PD21	14	3M/11F
	Cx3cr1^{CreER}:*Csf1r*$^{fl/fl}$	Mo transfer	PD21	14	5M/5F 4 unknown
	Cx3cr1^{CreER}:*Csf1r*$^{fl/fl}$	Mglia transfer	3mo	12	5M/6F 1 unknown
	Cx3cr1^{CreER}:*Csf1r*$^{fl/fl}$	Mo transfer	3mo	9	4M/5F
	Cx3cr1^{CreER}:*Csf1r*$^{fl/fl}$	Mglia transfer	10mo	9	5M/4F
	Cx3cr1^{CreER}:*Csf1r*$^{fl/fl}$	Mo transfer	10mo	9	6M/3F
	Cx3cr1^{CreER}:*Csf1r*$^{fl/fl}$	Mglia transfer	12mo	5	1M/4F
	Cx3cr1^{CreER}:*Csf1r*$^{fl/fl}$	Mo transfer	12mo	10	3M/7F
	Cx3cr1^{CreER}:*Csf1r*$^{fl/fl}$	Mglia transfer	20mo	3	2M/1F
	Cx3cr1^{CreER}:*Csf1r*$^{fl/fl}$	Mo transfer	20mo	3	2M/1F
2D	*Cx3cr1*^{CreER}:*Csf1r*$^{fl/fl}$	No tam	PD16	4	1M/3F
	Cx3cr1^{CreER}:*Csf1r*$^{fl/fl}$	Mglia transfer	PD16	8	6M/2F
	Cx3cr1^{CreER}:*Csf1r*$^{fl/fl}$	Mo transfer	PD16	6	4M/2F
	Cx3cr1^{CreER}:*Csf1r*$^{fl/fl}$	Tam+sham	3mo	3	3M
	Cx3cr1^{CreER}:*Csf1r*$^{fl/fl}$	No tam	3mo	4	3M/1F
	Cx3cr1^{CreER}:*Csf1r*$^{fl/fl}$	Mglia transfer	3mo	6	3M/3F
	Cx3cr1^{CreER}:*Csf1r*$^{fl/fl}$	Mo transfer	3mo	4	2M/2F
	Cx3cr1^{CreER}:*Csf1r*$^{fl/fl}$	No tam	12mo	3	2M/1F
	Cx3cr1^{CreER}:*Csf1r*$^{fl/fl}$	Mglia transfer	12mo	4	1M/3F
	Cx3cr1^{CreER}:*Csf1r*$^{fl/fl}$	Mo transfer	12mo	5	2M/3F
2E,F	*Cx3cr1*^{CreER}:*Csf1r*$^{fl/fl}$	Naive	4W	10	7M/3F

	Cx3cr1^{CreER}:*Csf1r*$^{fl/fl}$	Tam	4W	6	5M/1F
	Cx3cr1^{CreER}:*Csf1r*$^{fl/fl}$	Mo transfer	4W	7	3M/4F
2G	*Cx3cr1*^{CreER}:*Csf1r*$^{fl/fl}$	No tam	PD16	2	1M/1F
	Cx3cr1^{CreER}:*Csf1r*$^{fl/fl}$	Tam	PD16	2	1M/1F
	Cx3cr1^{CreER}:*Csf1r*$^{fl/fl}$	Tam	3mo	3	1M/2F
S2B	*Cx3cr1*^{CreER}:*Csf1r*$^{fl/fl}$	No tam	3mo	2	1M/1F
	Cx3cr1^{CreER}:*Csf1r*$^{fl/fl}$	Mglia transfer	3mo	3	2M/1F
	Cx3cr1^{CreER}:*Csf1r*$^{fl/fl}$	Mo transfer	3mo	3	2M/1F
S2C,D	*Cx3cr1*^{CreER}:*Csf1r*$^{fl/fl}$:5xFAD$^{+/-}$	Mglia transfer	5mo	4	1M/3F
	Cx3cr1^{CreER}:*Csf1r*$^{fl/fl}$:5xFAD$^{+/-}$	Mo transfer	5mo	4	4F
S2E	*Cx3cr1*^{CreER}:*Csf1r*$^{fl/fl}$	Mo transfer	4W	2	1M/1F
3B, S2F,G,H	*Cx3cr1*^{CreER}:*Csf1r*$^{fl/fl}$	Control	10W	7	4M/3F
	Cx3cr1^{CreER}:*Csf1r*$^{fl/fl}$	Tam C	10W	7	3M/4F
	Cx3cr1^{CreER}:*Csf1r*$^{fl/fl}$	Tam CI	10W	6	4M/2F
	Cx3cr1^{CreER}:*Csf1r*$^{fl/fl}$	1W PLX+Tam CI	10W	5	3M/2F
	Cx3cr1^{CreER}:*Csf1r*$^{fl/fl}$	2W PLX+Tam CI	11W	7	5M/2F
S2I	C57BL/6	Control	10W	5	1M/4F
	Cx3cr1^{CreER}:*Csf1r*$^{fl/fl}$	PLX+Tam CI	10-11W	5	3M/2F
3C,D	*Cx3cr1*^{CreER}:*Csf1r*$^{fl/fl}$	Control	10W	4	2M/2F
	Cx3cr1^{CreER}:*Csf1r*$^{fl/fl}$	Tam C	10W	4	3M/1F
	Cx3cr1^{CreER}:*Csf1r*$^{fl/fl}$	Tam CI	10W	7	4M/3F
	Cx3cr1^{CreER}:*Csf1r*$^{fl/fl}$	1W PLX+Tam CI	10W	6	5M/1F
	Cx3cr1^{CreER}:*Csf1r*$^{fl/fl}$	2W PLX+Tam CI	11W	7	4M/3F
3E	*Cx3cr1*^{CreER}:*Csf1r*$^{fl/fl}$	Naive	7W	4	4F
	Cx3cr1^{CreER}:*Csf1r*$^{fl/fl}$	PLX	7W	4	2M/2F
	Cx3cr1^{CreER}:*Csf1r*$^{fl/fl}$	PLX + Tam CI	7W	4	4M
3F-I, S2J	*Cx3cr1*^{CreER}:*Csf1r*$^{fl/fl}$	1W PLX+Tam CI	10W	4	4M
4A-H,S4A-C, S3A-C,F-K	*Flt3*^{Cre}:*Yfp*	Control	14W	8	M
	Flt3^{Cre}:*Yfp*	2W PLX	14W	7	M
S3D,E	C57BL/6	Control	10W	4	M
	Flt3^{Cre}:*Yfp*	PLX	10W	4	M
4I-O, S4A-K	*Cx3cr1*^{CreER}:*Csf1r*$^{fl/fl}$	adult	14W	4	M
	Cx3cr1^{CreER}:*Csf1r*$^{fl/fl}$	neonate	9W	3	F
5A-G,S4L-P	*Cx3cr1*^{CreER}:*Csf1r*$^{fl/fl}$	MDP-Mo-Mglia	6W	3	2M/1F
	Cx3cr1^{CreER}:*Csf1r*$^{fl/fl}$	GMP-Mo-Mglia	6W	3	2M/1F
5H-N, S4Q-V	*Cx3cr1*^{CreER}:*Csf1r*$^{fl/fl}$	FL neonates	9W	5	M
6, S5	C57BL/6	Fetal liver	E14	27	M/F
	C57BL/6	Bone marrow	10W	3	2M/1F
7A-G, S6B-I, L-R	*hCSF1*^{KI} x *Rag2*$^{-/-}$ x $\gamma c^{/-}$	Adult blood	4W	9	5M/4F
	hCSF1^{KI} x *Rag2*$^{-/-}$ x $\gamma c^{-/-}$	Cord blood	4W	8	3M/5F
7D,E	*hCSF1*^{KI} x *Rag2*$^{-/-}$ x $\gamma c^{-/-}$	Adult blood	4W	3	1M/2F
S6J,K	*hCSF1*^{KI} x *Rag2*$^{-/-}$ x $\gamma c^{-/-}$	Cord blood	4W	3	2M/1F
