# Supplementary material for: Monocytes can efficiently replace all brain macrophages and fetal liver monocytes can generate bona fide SALL1+ microglia
Source: Immunity. 2025 May 13;58(5):1269–1288.e12. doi: 10.1016/j.immuni.2025.04.006 (PMC12094688; doi:10.1016/j.immuni.2025.04.006)
Supplement: Document S2. Article plus supplemental information [file mmc3.pdf]

Article

Immunity

Monocytes can efficiently replace all brain macrophages and fetal liver monocytes can generate bona fide SALL1$^+$ microglia

Graphical abstract

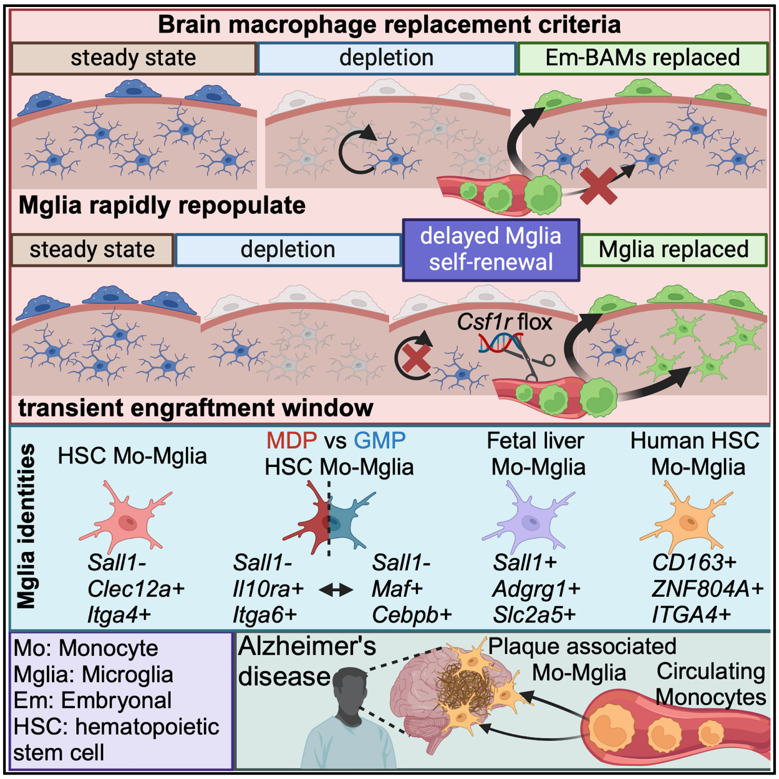

Highlights

- Monocytes efficiently replace microglia and border-associated macrophages
- Monocyte ontogeny shapes their identity as brain macrophages
- Fetal liver monocytes have altered chromatin and acquire a true microglial identity
- Putative monocyte-derived microglia identified in Alzheimer's patients

Authors

Jonathan Bastos, Carleigh O'Brien, Mónica Vara-Pérez, ..., David Gate, F. Chris Bennett, Kiavash Movahedi

Correspondence

kiavash.movahedi@vub.be

In brief

Replacing brain macrophages holds substantial therapeutic promise but remains challenging. Bastos et al. uncover the requirements for monocytes to replace microglia and border-associated macrophages. They show that monocyte ontogeny shapes brain macrophage identity, with fetal monocytes acquiring a true microglial state. Using xenotransplantation and Alzheimer's patient data, they identify human monocyte-derived microglia. These findings are important for microglia replacement therapies.

Bastos et al., 2025, Immunity 58, 1269–1288
May 13, 2025 © 2025 The Author(s). Published by Elsevier Inc.
https://doi.org/10.1016/j.immuni.2025.04.006

CellPress

Immunity

CellPress
OPEN ACCESS

Article

Monocytes can efficiently replace all brain macrophages and fetal liver monocytes can generate bona fide SALL1^{+} microglia

Jonathan Bastos,[1] Carleigh O'Brien,[2] Mónica Vara-Pérez,[1] Myrthe Mampay,[1] Lynn van Olst,[3] Liam Barry-Carroll,[4,5] Daliya Kancheva,[1] Sophia Leduc,[1] Ayla Line Lievens,[1] Leen Ali,[1] Vladislav Vlasov,[1] Laura Meysman,[1] Hadis Shakeri,[1] Ria Roelandt,[6,7] Hannah Van Hove,[1] Karen De Vlaminck,[1] Isabelle Scheyltjens,[1] Fazeela Yaqoob,[2] Sonia I. Lombroso,[2,8] Maria Breugelmans,[9] Gilles Faron,[9] Diego Gomez-Nicola,[4] David Gate,[3] F. Chris Bennett,[2,10] and Kiavash Movahedi[1,11,*]

[1]Brain and Systems Immunology Laboratory, Brussels Center for Immunology, Vrije Universiteit Brussel, Brussels, Belgium
[2]Department of Psychiatry, Perelman School of Medicine, University of Pennsylvania, Philadelphia, PA, USA
[3]The Ken & Ruth Davee Department of Neurology, Northwestern University Feinberg School of Medicine, Chicago, IL, USA
[4]School of Biological Sciences, Southampton General Hospital, University of Southampton, Southampton, UK
[5]Nutrineuro, UMR 1286 INRAE, Bordeaux University, Bordeaux INP, Bordeaux, France
[6]VIB Single Cell Core, VIB, Ghent/Leuven, Belgium
[7]Department of Biomedical Molecular Biology, Ghent University, Ghent, Belgium
[8]Department of Systems Pharmacology and Translational Therapeutics, University of Pennsylvania, Philadelphia, PA, USA
[9]Department of Obstetrics and Prenatal Medicine, UZ Brussel, VUB, Brussels, Belgium
[10]Division of Neurology, Children's Hospital of Philadelphia, Philadelphia, PA, USA
[11]Lead contact
*Correspondence: kiavash.movahedi@vub.be
https://doi.org/10.1016/j.immuni.2025.04.006

SUMMARY

Microglia and border-associated macrophages (BAMs) are critical for brain health, and their dysfunction is associated to disease. Replacing brain macrophages holds substantial therapeutic promise but remains challenging. Here, we demonstrate that monocytes can efficiently replace all brain macrophages. Monocytes readily replaced embryonal BAMs upon their depletion and engrafted as monocyte-derived microglia (Mo-Microglia) upon more sustained niche availability. Mo-Microglia expanded comparably to their embryonic counterparts and showed similar longevity. However, monocytes were unable to replicate the distinct identity of embryonically derived BAMs and microglia. Using xenotransplantation, we found that human monocytes exhibited similar behavior, enabling identification of putative Mo-Microglia in Alzheimer's disease individuals. In mice and humans, monocyte ontogeny shaped their identity as brain macrophages. Importantly, mouse fetal liver monocytes exhibited a distinct epigenetic landscape and could develop a bona fide microglial identity. Our results illuminate brain macrophage development and highlight monocytes as an abundant progenitor source for brain macrophage replacement therapies.

INTRODUCTION

The brain has developed a symbiotic relationship with macrophages. Brain-derived signals instruct and support macrophages that, in turn, help maintain healthy brain homeostasis. The macrophage phenotype is highly dependent on the nature of the tissue niche; as a result, macrophages exhibit clear diversity across brain compartments.[1,2] Microglia are phenotypically distinct from border-associated macrophages (BAMs) present in the meninges, perivascular spaces, and choroid plexus.[2,3] BAMs consist of two main populations, distinguished by major histocompatibility complex class II (MHC class II) expression among other markers.[2,3] A growing body of evidence links brain macrophage dysfunction to disease, as predicted by genome-wide association studies.[4,5] Therefore, cell-based replacement therapies that aim to correct or enhance microglia or BAMs hold great therapeutic potential but remain challenging.[6]

The initial model of the mononuclear phagocyte system proposed that all tissue-resident macrophages are continuously replaced by bone marrow (BM)-derived blood monocytes. While this is true for some tissue macrophage populations, including MHC class II^{hi} BAMs (Hi BAM),[2] it does not apply to all.[7] Microglia and MHC class II^{lo} BAMs (Lo BAM) in the leptomeninges are derived from primitive yolk sac (YS) macrophages that colonize the brain during early embryonic development.[8,9] Under steady-state conditions, these long-lived macrophages are not replaced by BM progenitors.[2,10] Remarkably, even upon widespread microglia depletion using colony-stimulating factor 1 receptor (CSF1R) inhibitors, embryonic microglia can self-renew and repopulate the brain, with no clear evidence for BM engraftment.[2,11,12]

This is an open access article under the CC BY license (http://creativecommons.org/licenses/by/4.0/).

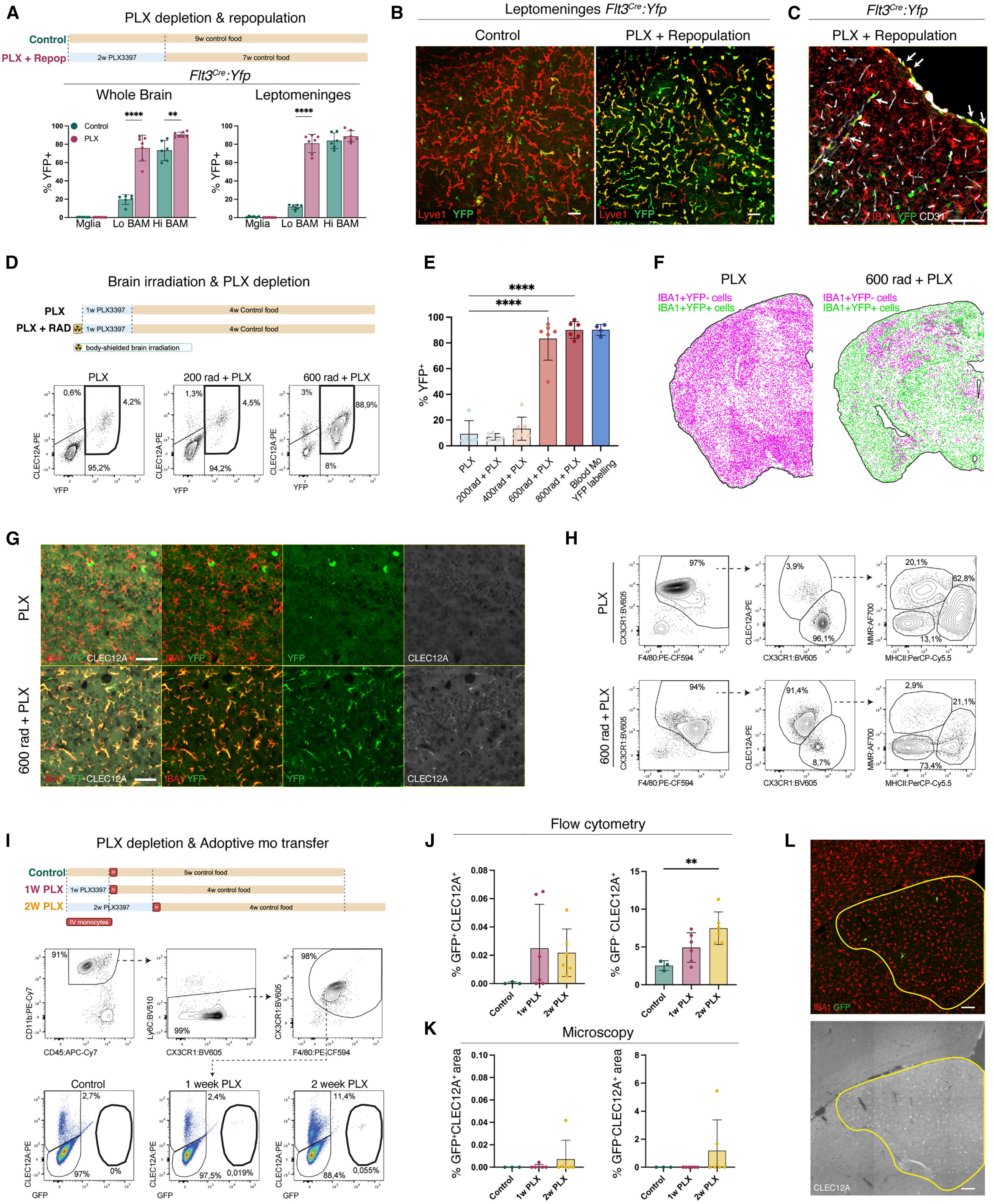

Figure 1. Monocytes can readily replace BAMs but are unable to engraft the brain parenchyma due to microglial self-renewal

(A) Top: experiment layout. *Flt3*^{Cre}*:Yfp* mice were fed for 2 weeks control or PLX3397 chow and then 7 weeks control chow. Flow cytometry of YFP^{+} microglia (Mglia), Lo and Hi BAMs in control and depleted-repopulated brains and leptomeninges. Gating strategy shown in Figure S1A. $n = 6$ for control and $n = 7$ for PLX from 2 independent experiments. Mean ± SD.

(legend continued on next page)

CellPress
OPEN ACCESS

It remains unclear why monocytes are unable to engraft a brain in which nearly all endogenous microglia are depleted. Monocytes do infiltrate the brain parenchyma during certain disease conditions but develop into short-lived macrophages that largely disappear upon disease resolution.[13,14] Microglia replacement by BM progenitors is observed upon whole-body myeloablation followed by hematopoietic stem cell (HSC) transplantation.[15–20] Several hypotheses propose why HSC transplantation induces microglia replacement. Myeloablation was shown to induce microglial senescence,[16] perhaps a prerequisite for BM engraftment. Myeloablative therapy may also alter the blood-brain barrier (BBB) and thereby allow progenitor infiltration.[20] Some studies have also suggested that the engrafting cells are not monocytes, but rather immature HSC progenitors, typically absent in the blood but temporarily introduced after BM injection.[18,20] A deeper understanding of the prerequisites for microglia replacement, coupled with knowledge of the progenitor cells that can engraft as microglia, is foundational for future microglia replacement strategies.

Seminal studies have shown that BM-derived microglia cannot attain the YS-microglial identity, notably lacking SALL1,[15,17,21,22] a key regulator of microglial gene expression.[23] Conversely, other embryonically derived tissue macrophages, such as Kupffer cells (KCs) and alveolar macrophages (AMs), can be faithfully replaced by HSC-derived progenitors.[24,25] The reason for this discrepancy in microglia remains unclear. Microglial identity may be intrinsically linked to their primitive YS macrophage origin or may relate to changes in the brain environment. It remains to be clarified to what extent these ontogeny-related findings also apply to humans.

Here, we combined brain macrophage transplantation paradigms with fate mapping, single-cell omics and human xenotransplantation to unravel the intricacies of brain macrophage replacement. We show that monocytes do have the intrinsic ability to efficiently replace all brain macrophages, including microglia. We provide insights into the prerequisites for this process and the dynamics of their engraftment. Monocyte-derived Lo BAMs retained a distinct transcriptional state compared with their embryonal counterparts. Both monocyte-dendritic-cell progenitor (MDP)- and granulocyte-monocyte progenitor (GMP)-derived monocytes developed into microglia-like cells, but these exhibited distinct gene signatures. Importantly, mouse fetal liver (FL) monocytes could develop into bona fide *Sall1*^{hi} microglia that were transcriptionally equivalent to their YS-derived counterparts. Transcriptional and epigenetic profiling revealed the distinct chromatin landscape of FL monocytes, including at the *Sall1* locus. Finally, by transplanting human adult or umbilical cord blood monocytes into mice, we demonstrate that human monocyte-derived microglia (Mo-Microglia) also maintain a distinct gene signature. This enabled us to identify a putative Mo-Microglia cluster in single-nucleus RNA sequencing (snRNA-seq) datasets of Alzheimer's disease (AD) patients, with Mo-Microglia abundance correlating with disease severity.

RESULTS

Monocytes replace embryonal BAMs after brain macrophage depletion but cannot efficiently engraft the brain parenchyma due to microglial self-renewal

In the homeostatic brain, monocytes gradually replenish BAMs within the dura mater and choroid plexus stroma, whereas Lo BAMs within the leptomeninges and perivascular spaces, along with microglia, are yolk-sac-derived and not replaced by BM progenitors.[2,10] To assess the ontogeny of repopulated brain macrophages following PLX3397 (PLX)-induced depletion, we relied on fate mapping using *Flt3*^{Cre}*:Yfp* mice.[26] Mice were fed high-dose PLX chow[11] for 2 weeks, followed by 7 weeks of repopulation, after which whole brains and leptomeningeal-enriched slices were analyzed via flow cytometry (Figure 1A). In controls,

(B) Whole-mount leptomeninges of controls or 10-week-post-PLX3397 repopulation, stained for Lyve1, GFP. Scale bar: 50 μm. n = 4 control, n = 6 PLX from 1 experiment.

(C) Section of mouse brain 4 weeks post-PLX3397, stained for IBA1, GFP, CD31. Arrows indicate YFP$^+$ perivascular and leptomeningeal BAMs. Scale bar: 100 μm. n = 3 from 1 experiment.

(D) Top: experiment layout. *Flt3*^{Cre}*:Yfp* mice were given different doses of brain-restricted irradiation followed by 1-week PLX3397. Repopulation occurred for 4 weeks (0 rad: n = 5, 200 rad: n = 6, 400 rad: n = 7, 600 rad: n = 6, 800 rad: n = 6, from 2 independent experiments). Flow cytometry of YFP$^+$ cells, pre-gated on CD11b$^+$CD45$^+$CX3CR1$^+$F4/80$^+$ as shown in Figure S1D.

(E) Flow cytometric quantification of %YFP$^+$ cells within CD11b$^+$CD45$^+$CX3CR1$^+$F4/80$^+$ cells from (D). Mean ± SD. Blood monocyte gating strategy shown in Figure S1F.

(F) Representative images of the spatial distribution of IBA1$^+$YFP$^+$ and IBA1$^+$YFP$^-$ cells in brain sections from (D). 0 rad: n = 5, 600 rad: n = 5, from 2 independent experiments.

(G) Brain sections of *Flt3*^{Cre}*:Yfp* mice from (F), stained for IBA1, GFP, CLEC12A. Scale bar: 50 μm.

(H) Flow cytometry of brains from *Flt3*^{Cre}*:Yfp* mice from (D).

(I) Top: C57BL/6 mice received control or PLX3397 chow for 1–2 weeks. All groups received an adoptive transfer of *Cx3cr1*$^{GFP/+}$ monocytes. Mice were then transferred to control chow for 4 weeks. Brain cells pre-gated as CD45$^+$, single, live cells (Figure S1A). n = 3 (control), n = 6 (1 week PLX), n = 6 (2 weeks PLX). Control: 1 experiment, 1 and 2 weeks PLX: 2 independent experiments.

(J) Percentage of GFP$^+$CLEC12A$^+$ and GFP$^-$CLEC12A$^+$ engraftment from (I). Mean ± SD.

(K) Percentage of GFP$^+$CLEC12A$^+$ and GFP$^-$CLEC12A$^+$ engraftment area from (I). n = 3 (control), n = 6 (1 week PLX), n = 6 (2 weeks PLX). Mean ± SD. Control: 1 experiment, 1 and 2 weeks PLX: 2 independent experiments.

(L) Brain section of mouse from 2-week-PLX3397 group (see I and K). Staining: anti-IBA1, anti-GFP, anti-CLEC12A. Perimeter depicts area of CLEC12A$^+$ engraftment. Scale bar: 250 μm.

Statistical tests: (A) significance for each "control vs. PLX" comparison determined using separate unpaired two-tailed t tests. (E, J, and K) Significances were determined using Dunnett's multiple comparisons test, groups were compared with the control group.

ns, $^*p < 0.05$, $^{**}p < 0.01$, $^{***}p < 0.001$, $^{****}p < 0.0001$.

See also Figure S1.

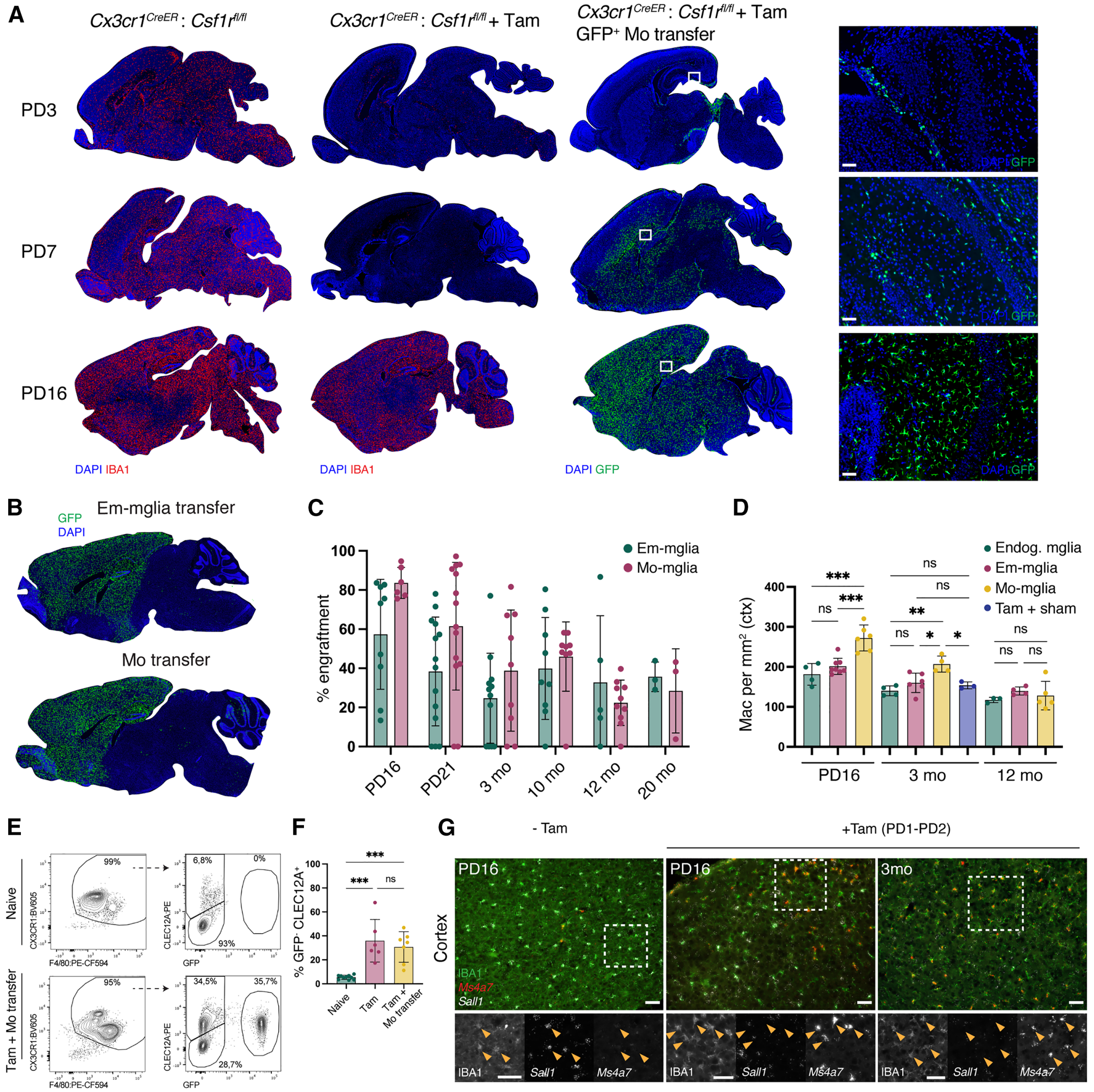

Figure 2. Extending niche availability allows monocytes to efficiently engraft as long-lived Mo-Microglia, comparable to their embryonic counterparts

(A–D) Tamoxifen (Tam) was administered to *Cx3cr1*^{CreER}:*Csf1r*$^{fl/fl}$ neonate mice at post-natal day (PD) 1 and 2. Left column: untreated mice; middle column: mice received Tam; right column: mice received Tam and intracerebrally injected monocytes. Brains collected at PD3, PD7, and PD16. (A) Section stained for IBA1. Insets shown for mice that received Tam + monocytes. Scale bar: 50 μm. Images representative of PD3: *n* = 3 untreated, *n* = 2 Tam, *n* = 2 Tam + monocytes. PD7: *n* = 3 untreated, *n* = 3 Tam, *n* = 3 Tam + monocytes. PD16: *n* = 4 untreated, *n* = 3 Tam, *n* = 8 Tam + monocytes. Untreated and Tam: 2 independent experiments. PD3 and PD7 Tam + monocytes: 1 and PD16: 2 independent experiments. (B) GFP$^+$ Em-Mglia (150,000) or GFP$^+$ monocytes (150,000) were intracerebrally injected at PD3. Brains collected at 3 months. (C) Quantification of the total engrafted area from Em-Mglia or monocyte engrafted brains in (B). Mo transfer: *n* = 6 (PD16), *n* = 14 (PD21), *n* = 9 (3 months), *n* = 9 (10 months), *n* = 10 (12 months), *n* = 3 (20 months). Mglia transfer: *n* = 9 (PD16), *n* = 14 (PD21), *n* = 12 (3 months), *n* = 9 (10 months), *n* = 5 (12 months), *n* = 3 (20 months). Mean ± SD. Data from 6 independent experiments. (D) Quantification of cortical macrophage density at PD16, 3 and 12 months of age post-transplantation. Endog. Mglia: *n* = 4 (PD16), *n* = 4 (3 months), *n* = 3 (12 months). Em-Mglia: *n* = 8 (PD16), *n* = 6 (3 months), *n* = 4 (12 months). Mo-Mglia: *n* = 6 (PD16), *n* = 4 (3 months), *n* = 5 (12 months). Tam + Sham: *n* = 3 (3 months). Endog. Mglia: 2, Em-Mglia: 6, Mo-Mglia: 6, and Tam + Sham: 1 independent experiment(s). Mean ± SD.

(E and F) Representative flow cytometry of Mo-Mglia engraftment in *Cx3cr1*^{CreER}:*Csf1r*$^{fl/fl}$ mice. Experiment identical to (A), compared with naive mice. (F) Quantification of GFP$^-$CLEC12A$^+$ Mo-Mglia engraftment. *n* = 10 naive, *n* = 6 Tam, and *n* = 7 Tam + monocytes. Naive: 3, Tam and Tam + monocytes: 2 independent experiments. Mean ± SD.

(G) *In situ* hybridization of *Ms4a7* and *Sall1* combined with IBA1 immunostaining in the cortex following Tam administration as in (A) (no cells injected). Left column insets: arrowheads indicating *Sall1*$^+$ Em-Mglia in naive mice at PD16. Column 2 and 3 insets: arrowheads indicating *Ms4a7*$^+$ Mo-Mglia at PD16 and 3 months. Scale bar: 50 μm. Representative images of *n* = 2 no Tam PD16, *n* = 2 Tam PD16, and *n* = 3 Tam 3 months, from 2 independent experiments.

(legend continued on next page)

microglia and Lo BAMs showed limited YFP labeling, while most Hi BAMs were BM-derived (Figures 1A and S1A), consistent with previous observations.[2] After PLX-induced depletion, YFP$^-$ microglia repopulated, whereas embryonal Lo BAMs were almost entirely replaced by YFP$^+$ BM-derived cells (Figure 1A). This was confirmed via histology, revealing the replacement of leptomeningeal and perivascular BAMs with YFP$^+$ counterparts (Figures 1B and 1C). To investigate whether blood monocytes replace leptomeningeal and perivascular BAMs, C57BL/6 mice were fed PLX chow for 1 week, followed by intravenous injections of magnetic-activated-cell-sorting (MACS)-purified *Cx3cr1*$^{GFP/+}$ BM monocytes (Figure S1B). 4 weeks later, GFP$^+$ BAMs were observed in the leptomeninges and perivascular spaces (Figures S1C and S1D).

We wondered what factors are preventing monocyte engraftment in the brain parenchyma and focused on the role of microglial self-renewal. *Flt3*^{Cre}*:Yfp* mice were subjected to varying doses of brain-restricted irradiation, which may induce microglial senescence,[18,27] followed by 1 week of PLX treatment and 4 weeks of repopulation. Starting from 600 rad, PLX treatment induced a near-complete replacement of embryonal microglia (Em-Microglia) by YFP$^+$ BM-derived cells as observed by flow cytometry (Figures 1D, 1E, and S1E) and histology (Figures 1F and S1F). At 600 rad, 83.3% ± 6.8% of brain macrophages were YFP labeled, comparable to blood monocytes (Figures 1E and S1G), suggesting full replacement. Since no BM injection was performed, YFP$^+$ microglia originated from naturally trafficking progenitors, presumably monocytes. The putative Mo-Microglia showed a distinct morphology with fewer ramifications (Figure 1G) and were readily distinguishable from Em-Microglia via flow cytometry based on their CX3CR1^{lo}F4/80^{hi} profile and CLEC12A expression (Figure 1H), consistent with previous reports.[22] These results suggest that impairing microglial self-renewal via irradiation enables their replacement by monocyte-derived progenitors following PLX-induced depletion. Alternatively, irradiation may induce neuroinflammation or BBB disruption, facilitating progenitor infiltration. To test whether monocytes can infiltrate a vacant brain parenchyma without irradiation, we sorted *Cx3cr1*$^{GFP/+}$ BM monocytes and intravenously injected them in mice after 1 or 2 weeks of PLX chow (Figure 1I). Flow cytometry detected very few GFP$^+$ cells in the brain (Figure 1I). However, in mice treated with PLX for 2 weeks, a small but significant increase in GFP$^-$CLEC12A$^+$ cells was observed, suggestive of Mo-Microglia (Figure 1J). Histology further revealed CLEC12A$^+$ microglia patches, sometimes containing GFP$^+$ cells (Figures 1K and 1L). This indicates that small numbers of monocytes engrafted the brain parenchyma of mice that received PLX chow for 2 weeks, a treatment regimen in which Em-Microglia have been shown to repopulate slower.[11] Together, these results suggest that monocyte can infiltrate and expand in the brain after microglia depletion, but their engraftment remains inefficient due to competition with repopulating Em-Microglia.

A transiently vacant and engraftable niche enables monocytes to efficiently develop into long-lived microglia-like cells in the neonate brain

To assess monocyte engraftment in a setting of transiently impaired Em-Microglia repopulation, we developed a genetic approach using *Cx3cr1*^{CreER}*:Csf1r*$^{fl/fl}$ mice. Two consecutive tamoxifen injections at PD1 and PD2 in neonates led to extensive, prolonged Em-Microglia depletion, with repopulation occurring by PD16 (Figures 2A and S2A). Intracerebral injection of GFP$^+$ monocytes at PD3 resulted in widespread engraftment, and by PD16, GFP$^+$ Mo-Microglia were distributed throughout the brain (Figure 2A). To directly compare monocyte and Em-Microglia engraftment capacity, freshly isolated GFP$^+$ brain microglia were transplanted in tamoxifen-treated *Cx3cr1*^{CreER}*:Csf1r*$^{fl/fl}$ mice at PD3 (Figure 2B). Both cell types engrafted and expanded similarly, persisting up to 20 months post-transplantation (Figure 2C). Mo-Microglia were less ramified than endogenous or transplanted Em-Microglia (Figure S2B) and initially reached higher cell densities but leveled out by 12 months (Figure 2D). To examine their persistence in a pathological context, *Cx3cr1*^{CreER}*:Csf1r*$^{fl/fl}$ mice were crossed with the 5xFAD amyloid pathology model.[28] Mo- and Em-Microglia engraftment percentages were comparable at 6 months post-transplantation, a time point at which there was extensive amyloid pathology (Figures S2C and S2D).

At 4 weeks post-monocyte transplantation, flow cytometry revealed an increase in both GFP$^+$ and GFP$^-$ CX3CR1^{lo}F4/80^{hi}CLEC12A$^+$ cells (Figures 2E and 2F), suggestive of Mo-Microglia derived from transplanted and endogenous monocytes, respectively. Microscopy confirmed GFP$^+$CLEC12A$^+$ and GFP$^-$CLEC12A$^+$ cells with Mo-Microglia morphology in the brain parenchyma (Figure S2E). To confirm their BM origin, we used *in situ* hybridization to assess *Sall1* and *Ms4a7* expression, key markers of Em- and BM-derived Microglia, respectively.[15,17,21,22] While microglia in untreated *Cx3cr1*^{CreER}*:Csf1r*$^{fl/fl}$ mice were *Sall1*$^+$ and *Ms4a7*$^-$, many IBA1$^+$ cells in tamoxifen-treated mice were *Sall1*$^-$*Ms4a7*$^+$, indicative of BM-microglia (Figure 2G). This confirms that tamoxifen treatment alone, even without intracerebral injections, enables endogenous BM progenitors to engraft as Mo-Microglia. Together, these findings show that a temporary absence of Em-Microglia allows monocytes to differentiate into microglia-like cells, which then exhibit similar expansion and persistence capacity as Em-Microglia.

A transiently vacant and engraftable niche allows peripheral blood monocytes to engraft the adult brain via clonal expansion

The neonatal brain may be particularly receptive to engraftment and has been reported to transiently attract monocytes.[29,30] Thus, we wondered whether the dynamics of monocyte engraftment would be different in adults. Cronk et al. have demonstrated that feeding tamoxifen chow to adult *Cx3cr1*^{CreER}:

Statistical tests: (C) Significance was determined based on a two-way ANOVA statistical test (Sidak's multiple comparison) where the means of each group were compared at each time point. (D) Significance was determined using Tukey's multiple comparisons test per age group. (F) Significance was determined using Tukey's multiple comparisons.
ns, $*p < 0.05$, $**p < 0.01$, $***p < 0.001$, $****p < 0.0001$.
See also Figure S2.

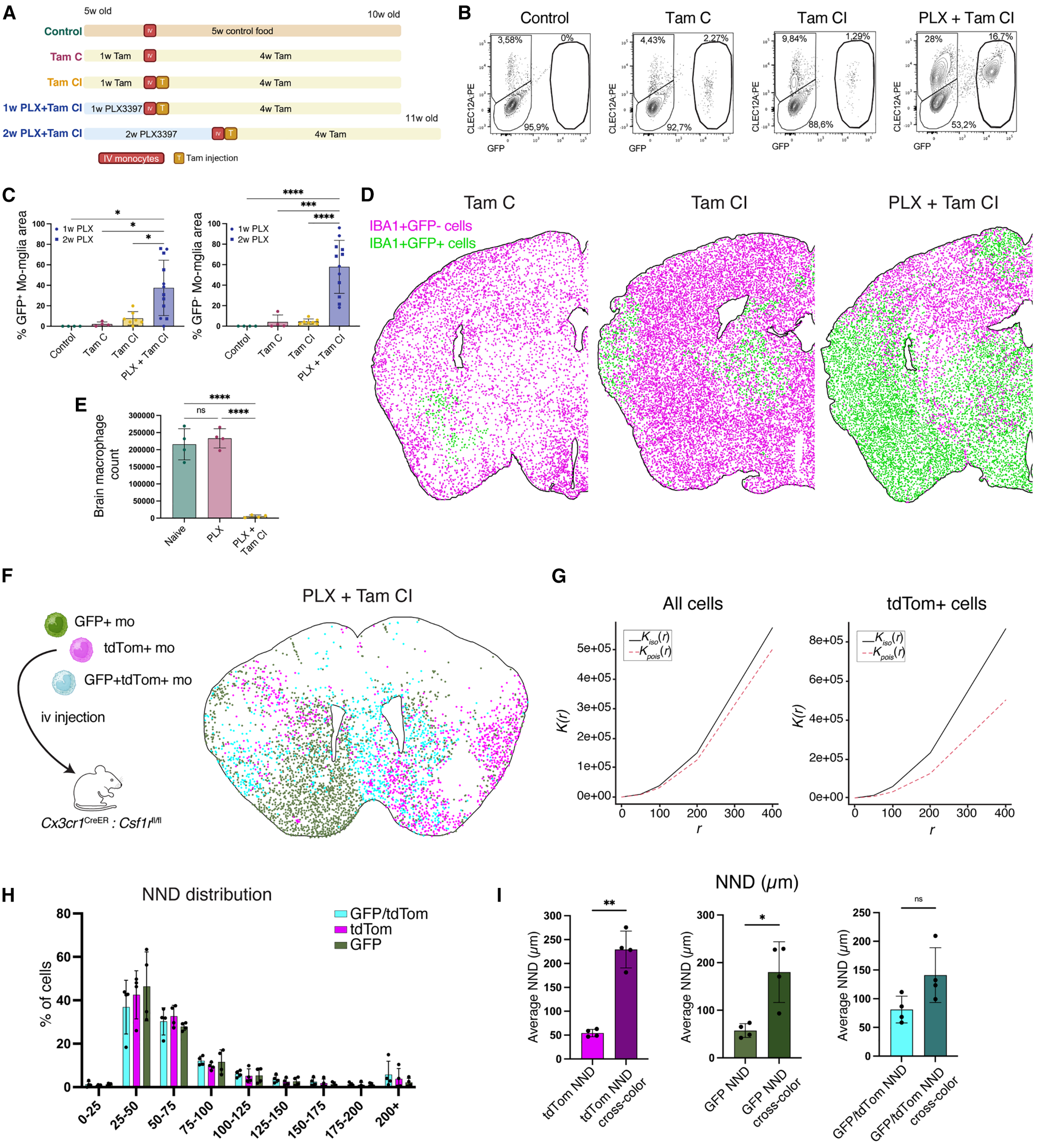

Figure 3. Transient niche availability allows peripheral monocytes to engraft as Mo-Microglia that undergo clonal expansion

(A) Experiment layout of Mo-Microglia transplantation in adults. Detailed explanation in Figure S2F.

(B) Representative gates distinguishing Em-Mglia (GFP$^-$CLEC12A$^-$), transplanted Mo-Mglia (GFP$^+$CLEC12A$^+$), and endogenous Mo-Mglia (GFP$^-$CLEC12A$^+$). Gating strategy in Figure S2F. *n* = 7 (control), *n* = 7 (TamC), *n* = 6 (TamCI), *n* = 5 + 7 (1/2 weeks PLX + TamCI, respectively). Control and TamC: 2, TamCI: 3, and 1/2 weeks PLX + TamCI: 4 independent experiments.

(C) Area analysis of brain sections from (A). Left: brain area percentage of GFP$^+$ transplanted Mo-Mglia engraftment. Right: brain area percentage of GFP$^-$ endogenous Mo-Mglia engraftment. *n* = 4 (control), *n* = 4 (TamC), *n* = 7 (TamCI), *n* = 5 + 7 (1/2 weeks PLX + TamCI, respectively). Mean ± SD. Control: 1, TamC and TamCI: 2, and 1/2 weeks PLX + TamCI: 4 independent experiments.

(D) Representative images of the spatial distribution of IBA1$^+$YFP$^+$ and IBA1$^+$YFP$^-$ cells in brain sections from experiment in (A).

(legend continued on next page)

CellPress
OPEN ACCESS

Csf1r$^{fl/fl}$ mice depletes ~25% of Em-Microglia, enabling low percentages of Mo-Microglia engraftment following monocyte transfer.[15] To investigate this further, *Cx3cr1*^{CreER}:*Csf1r*$^{fl/fl}$ mice were fed tamoxifen chow for 1 week, followed by an intravenous injection of GFP^{+} WT monocytes (TamC) (Figure 3A). A separate cohort additionally received a subcutaneous tamoxifen injection 2 days post-monocyte transfer (TamCI), to further increase tamoxifen exposure. While GFP^{+} Mo-Microglia were present 4 weeks post-transfer, their numbers were low, as quantified by flow cytometry (Figures 3B, S2F, and S2G) and microscopy (Figures 3C and 3D). A likely explanation is that the low percentage of Em-Microglia depletion, coupled with their active repopulation, prevents efficient Mo-Microglia engraftment. We hypothesized that pre-treating *Cx3cr1*^{CreER}:*Csf1r*$^{fl/fl}$ mice with PLX to deplete microglia, followed by tamoxifen administration, would slow Em-Microglia repopulation. To investigate this, mice received PLX for 1 week before switching to a control or tamoxifen diet. In the control group, microglia fully repopulated within 1 week, whereas repopulation was blocked in tamoxifen-treated mice (Figure 3E). Next, mice received PLX for 1 or 2 weeks, followed by an intravenous GFP^{+} monocyte injection and transfer to a tamoxifen diet. This led to a strong increase in GFP^{+} Mo-Microglia engraftment (Figures 3B–3D, S2F, and S2G), with similar engraftment in the 1- and 2-week PLX groups (Figure S2H). As expected, engrafted Mo-Microglia displayed morphological differences compared with Em-Microglia (Figure S2I). We also observed high numbers of GFP^{-} CLEC12A^{+} Mo-Microglia, indicating endogenous monocyte engraftment. Since host Mo-Microglia share the *Cx3cr1*^{CreER}:*Csf1r*$^{fl/fl}$ genotype, they would remain sensitive to tamoxifen. This suggests that Mo-Microglia engraftment does not require a continuous competitive advantage, but temporary niche availability, allowing both endogenous and intravenously injected monocytes to engraft and subsequently compete with Em-Microglia.

To assess whether Mo-Microglia engraftment relied on clonal expansion, PLX + Tam-treated *Cx3cr1*^{CreER}:*Csf1r*$^{fl/fl}$ mice were intravenously injected with equal ratios of GFP^{+}, tdTomato^{+} or GFP^{+}tdTomato^{+} monocytes. Large clusters of same-color Mo-Microglia were frequently observed, suggesting clonal expansion (Figure 3F). To quantify this, we performed a Ripley's K function analysis,[31] comparing the spatial distribution of labeled Mo-Microglia with a theoretical Poisson distribution. The observed Kr values were significantly higher for fluorescently labeled Mo-Microglia, indicating that they were more clustered than the total or unlabeled microglia population (Figures 3G and S2J). The nearest neighbor distance (NND) distribution showed that most same-color Mo-Microglia were <50 μm apart (Figure 3H), and their NND was lower than across colors, further supporting clonal expansion (Figure 3I).

The YS ontogeny underlies a distinct phenotype in both microglia and BAMs that cannot be replicated post-natally by BM monocytes

Previous work has shown that BM-derived microglia remain transcriptionally distinct from Em-Microglia,[15,17,21,22] but it is unclear whether an embryonal origin also underlies a distinct transcriptional state in BAMs. To investigate this, *Flt3*^{Cre}:YFP mice received 2 weeks of PLX chow, followed by 7 weeks of repopulation. Leptomeningeal-enriched slices or whole brains were collected and the replacement of YFP^{-} embryonal BAMs by YFP^{+} monocyte-derived cells was confirmed via flow cytometry (Figure S3A). Next, CLEC12A^{+} myeloid cells were fluorescence-activated cell sorting (FACS)-sorted to enrich for BAMs and potential Mo-Microglia, followed by single-cell RNA sequencing (scRNA-seq) (Figures 4A and S3A). Unsupervised analysis of control and repopulated leptomeningeal samples revealed various myeloid cell populations, including a small number of Em-Microglia (*Tmem119*^{hi}, *Olfml3*^{hi}, *Sall1*$^{+}$) that were co-sorted (Figures 4B and 4C; Figure S3B). Leptomeningeal BAMs were identified as *C1qa*$^{+}$*Fcgr1*$^{+}$*Ms4a6ac*$^{+}$ cells that were low for microglial signature genes. As reported previously,[2] BAMs could be subdivided into Lo BAM (*Mrc1*^{hi}*Lyve1*$^{+}$*Folr2*$^{+}$) and Hi BAM (*H2-Aa*$^{+}$*Cd74*$^{+}$*Fxyd5*$^{+}$), along with intermediate (Int BAM) interferon (IFN)-responsive (*Ifit2*$^{+}$*Ifit3*$^{+}$*Isg15*$^{+}$, IFN BAM) or immediate-early gene-responsive (*Egr1*$^{+}$*Ier3*$^{+}$*Fos*$^{+}$, IER BAM) subtypes (Figures S3B and S3C). We observed two distinct Lo BAM clusters, with Lo BAM1 derived almost exclusively from control samples, representing embryonic BAMs, while Lo BAM 2 primarily originated from repopulated brains, representing monocyte-derived BAMs (Figures 4D and 4E). In contrast, Hi BAMs and most other myeloid cell populations co-clustered in the control and repopulated conditions. Lo BAM1 and Lo BAM2 showed highly distinct gene expression profiles. Both expressed canonical Lo BAM markers (e.g., *Mrc1*, *Lyve1*, *Clec4n*, and *Clec10a*),[2] but certain Lo BAM1 genes, such as *Colec12*, *Ptprk*, *Cd163*, and *Auts2*, were absent or downregulated in Lo BAM2 (Figures 4C, 4F, 4G, and S3C). Gene Ontology (GO) analysis of genes enriched in Lo BAM1 revealed terms related to endocytosis, cell morphogenesis, and regulation of bone mineralization (Figure 4H), whereas Lo BAM2 showed enrichment in immune activation

(E) Quantification of brain macrophages in *Cx3cr1*^{CreER}:*Csf1r*$^{fl/fl}$ mice after 2 weeks of treatment: control, PLX (1 week PLX + 1 week control chow), or PLX + TamCI (1 week PLX + 1 week TamCI), experiment adapted from (A). Cells gated as in Figure S2F. $n = 4$ (control), $n = 4$ (PLX), and $n = 4$ (PLX + TamCI), from 2 independent experiments. Mean ± SD.

(F) The PLX + TamCI method was used as described in (A). *Cx3cr1*$^{GFP/+}$, *Cx3cr1*$^{tdTom/+}$, and *Cx3cr1*$^{GFP/tdTom}$ monocytes were transplanted in *Cx3cr1*^{CreER}:*Csf1r*$^{fl/fl}$ mice. Representative image of the spatial distribution of GFP^{+}, tdTomato^{+}, and GFP^{+}tdTomato^{+} macrophages. $n = 4$, from 1 experiment.

(G) Representative Ripley's K functions (K(r)) for cells derived from PLX + TamCI (F). Left: all cells (Em-Mglia, host Mo-Mglia, and transplanted Mo-Mglia), right: tdTomato^{+} cells. Red dotted and black line represent the theoretical K(r) and observed K(r), respectively.

(H) Frequency distribution of Mo-Mglia NND in PLX + TamCI mice (F). Mean ± SD.

(I) Quantification of average NND of Mo-Mglia in (F). Average values calculated for same-colored cells, and for each colored cell compared with the other two colors combined (cross-color). Mean ± SD.

Statistical tests: (C and E) Significance was determined using Tukey's multiple comparisons test. (I) Significances were determined using paired two-tailed t tests. ns, $^{*}p < 0.05$, $^{**}p < 0.01$, $^{***}p < 0.001$, $^{****}p < 0.0001$.

See also Figure S2.

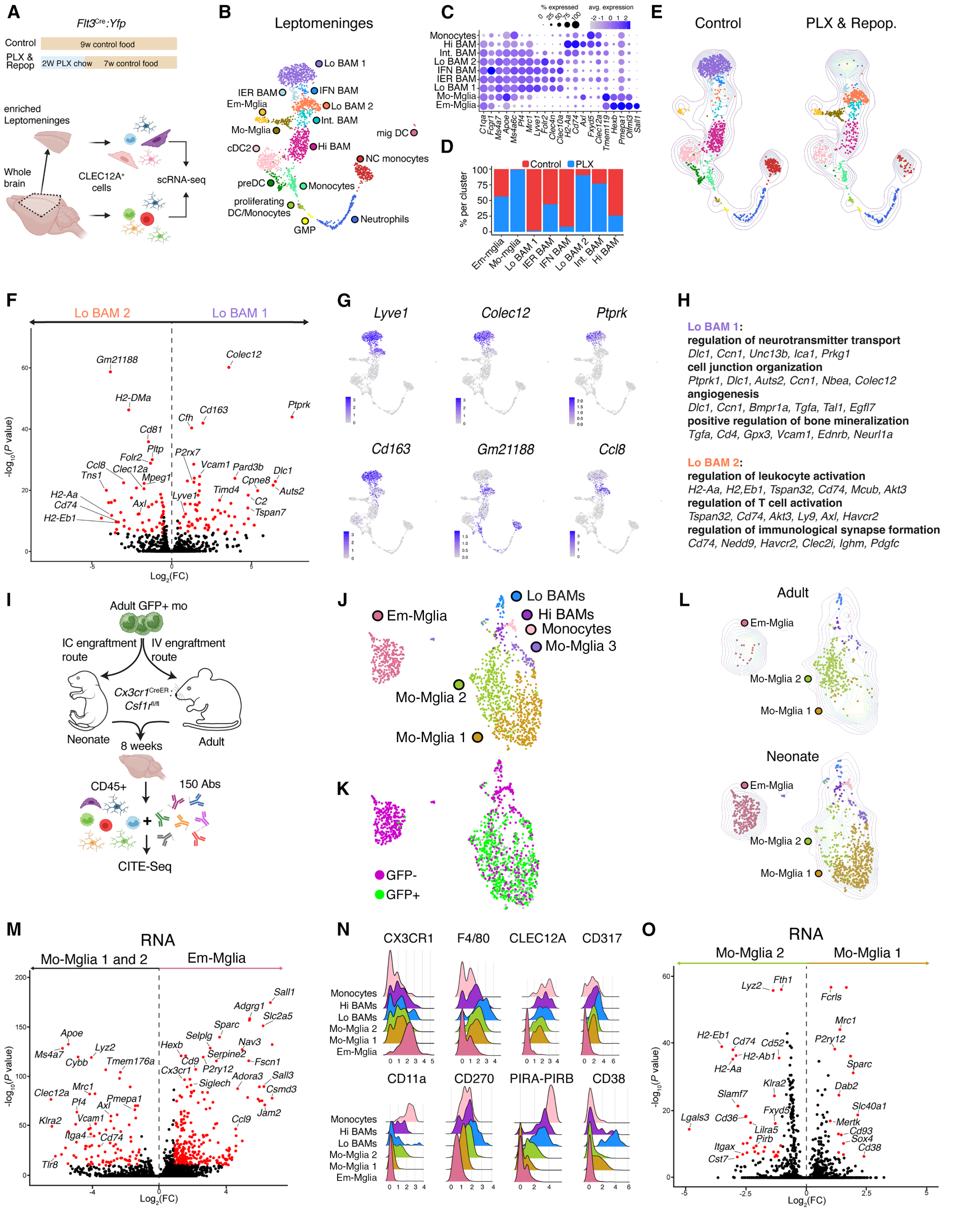

Figure 4. Adult BM monocytes cannot replicate the transcriptional identity of embryonically derived BAMs and microglia

(A) scRNA-seq of brain and leptomeningeal CLEC12A+ myeloid cells from control or repopulated brains. Detailed explanation and sorting strategy in Figure S3A. *n* = 8 (control) and *n* = 7 (PLX&Repop), from 1 experiment.

(B) UMAP plot of immune cells from leptomeningeal control and PLX&Repop conditions.

(legend continued on next page)

and antigen presentation pathways (Figures 4F–4H). To confirm that these transcriptional differences were reflected at the protein level, we analyzed CD163 expression, one of the most downregulated genes in Lo BAM2 (Figures 4F and 4G). In control brains, most LYVE1$^+$ leptomeningeal BAMs showed high CD163 expression, but this was lost upon repopulation (Figures S3D and S3E). To investigate whether ontogeny-dependent transcriptional differences extended beyond the leptomeninges, macrophages from the whole-brain single-cell dataset (Figure S3F) were analyzed separately (Figure S3G). This confirmed the presence of Lo BAM1 and Lo BAM2 clusters in whole-brain samples, again corresponding to control and repopulated conditions, respectively (Figures S3H–S3K). Together, these results reveal that monocyte-derived Lo BAMs that develop upon PLX-induced replacement remain molecularly distinct from their embryonal counterparts within the leptomeninges and perivascular spaces.

In addition to BAM populations, we identified a cluster (termed Mo-Mglia) nearly exclusive to repopulated conditions. These cells expressed both BAM-associated genes (e.g., *Ms4a7*, *Ms4a6c*, and *Clec12a*) and microglial markers (*Tmem119*, *Olfml3*, and *Pmepa1*) (Figures S3C, S3J, and S3K). However, key microglial genes, specific to Em-Microglia,[15,17,21,22] including *Sall1*, *Adgrg1*, and *Csmd3*, were absent (Figures S3C, S3J, and S3K). These cells were therefore identified as putative Mo-Microglia, consistent with our earlier observation that monocytes can engraft the parenchyma following 2 weeks of PLX treatment. To further validate this, GFP$^+$ monocytes were transplanted into *Cx3cr1*^{CreER}:*Csf1r*$^{fl/fl}$ mice to track GFP$^+$ Mo-Microglia engraftment (Figure 4I). To include protein validation, we performed cellular indexing of transcriptomes and epitopes (CITE-seq) using a panel of 150 barcoded antibodies (Table S3). Additionally, to investigate whether neonatal and adult brain environments differentially influence monocyte-to-microglia differentiation, monocyte transplantations were performed in neonates (intracerebral) and adults (intravenous) (Figures S4A and S4B). Brains were collected 8 weeks post-monocyte transplantation and CD45$^+$ cells were FACS-sorted for CITE-seq (Figure S4C). Unsupervised analysis identified various immune cell populations (Figures S4D–S4G), of which monocytes and macrophages were reclustered separately (Figure 4J). GFP-expressing cells were observed in 3 main clusters (Mo-Mglia1–3) that segregated from Em-Microglia and BAMs (Figure 4K). Mo-Mglia1 and Mo-Mglia2 were primarily derived from the neonate-injected and adult-injected conditions, respectively (Figures 4L and S4H). Mo-Mglia exhibited a distinctive gene expression signature as compared with Em-Microglia (Figures 4M and S4I), mirroring what was observed for Mo-Microglia in PLX repopulated brains (Figures S3J and S3K). Em- and Mo-Microglia also exhibited many differentially expressed (DE) proteins, including CLEC12A, CD317, F4/80, and CD270 (Figures 4N and S4J). The neonate-enriched Mo-Mglia1 cluster exhibited higher expression of microglial genes (*P2ry12*, *Fcrls*, and *Sparc*), whereas the adult-enriched Mo-Mglia2 cluster showed increased expression of immune activation genes (*H2-Aa*, *Slamf7*, *Itgax*) (Figure 4O). These differences were also confirmed at the protein level (Figures 4N and S4K). These findings suggest that the neonatal and adult brain environments distinctly influence Mo-Microglia differentiation, yet neither induces a *Sall1*$^+$*Ms4a7*$^-$ Em-Microglia-like state.

GMP-derived and MDP-derived monocytes both differentiate into microglia but retain distinctive identities

Classical monocytes can derive from GMPs or MDPs,[32–34] with recent studies showing that these progenitors differentially contribute to peripheral tissue macrophages.[32] To investigate whether both populations can develop into microglia-like cells, we FACS-sorted each population from *Cx3cr1*$^{Gfp/+}$ BM using the gating strategy developed by Trzebanski et al., which differentiates GMP- and MDP-derived monocytes based on the expression of CD177 and CD319, respectively[32] (Figures 5A, 5B, S4L, and S4M). GMP-Mo or MDP-Mo were intracerebrally injected in tamoxifen-treated *Cx3cr1*^{CreER}:*Csf1r*$^{fl/fl}$ neonates. 6 weeks post-transplantation, the percentage of GFP$^+$ brain-engrafted cells was comparable between the GMP-Mo and MDP-Mo groups (Figures S4N and S4O), indicating similar capacities for Mo-Microglia development. To further investigate this, GFP$^+$ cells were sorted for scRNA-seq analysis. Uniform manifold approximation and projection (UMAP) of GMP-Mo and MDP-Mo-derived cells revealed that most engrafted cells represented Mo-Microglia, alongside smaller fractions of Lo and Hi BAMs (Figure 5C). GMP-Mo and MDP-Mo-Microglia showed segregation, indicating differential gene expression (Figure 5C). We therefore relied on Harmony[35] to integrate the clusters from both samples and analyze whether GMP- and MDP-Mo-derived microglia adopt comparable cell states. Both progenitors generated heterogeneous Mo-Microglia, displaying states related to cytokine signaling, inflammation, and lipid metabolism

(C) Dot plot depicting gene expression of clusters in (B). Dot size represents percentage of cells expressing the gene, color legend represents average expression.
(D) Percentage of cells derived from control or PLX&Repop conditions from (B). Cell proportions were normalized per sample.
(E) UMAPs depicting cells from the control and PLX&Repop conditions separately. Contour lines represent cell density of the combined dataset.
(F) Volcano plot (VP) comparing DE genes between Lo BAM1 and Lo BAM2 clusters in (B). FC, fold change.(Log_2(FC) > 0.5, $-log_{10}$(adjusted p value) > 5).
(G) UMAPs showing expression of the indicated genes from (B).
(H) Chosen terms retrieved from GO enrichment analysis on DE genes from (F).
(I) CITE-seq to study Mo-Mglia engraftment in adult and neonate *Cx3cr1*^{CreER}:*Csf1r*$^{fl/fl}$ mice. Detailed explanation in Figure S4A. Sorting strategy in Figure S4C. n = 4 (adult) and n = 3 (neonate), from 1 experiment.
(J) UMAP of the macrophages from “neonate” and “adult” conditions.
(K) UMAP showing presence of GFP$^+$ cells (green cells: ≥3 *Gfp* transcripts, gray cells: 1–2 *Gfp* transcripts) and GFP$^-$ cells (magenta).
(L) UMAPs depicting cells from the adult or neonate conditions.
(M) VP comparing DE genes between Em-Mglia and the combined “Mo-Mglia1 and Mo-Mglia2” clusters in (J). (Log_2(FC) > 0.5, $-log_{10}$(adjusted p value) > 5).
(N) Ridge plots showing expression of selected proteins from (J) as detected via CITE-seq.
(O) VP comparing DE genes between Mo-Mglia1 and Mo-Mglia2 clusters in (J). (Log_2(FC) > 1, $-log_{10}$(adjusted p value) > 5).
See also Figures S3 and S4.

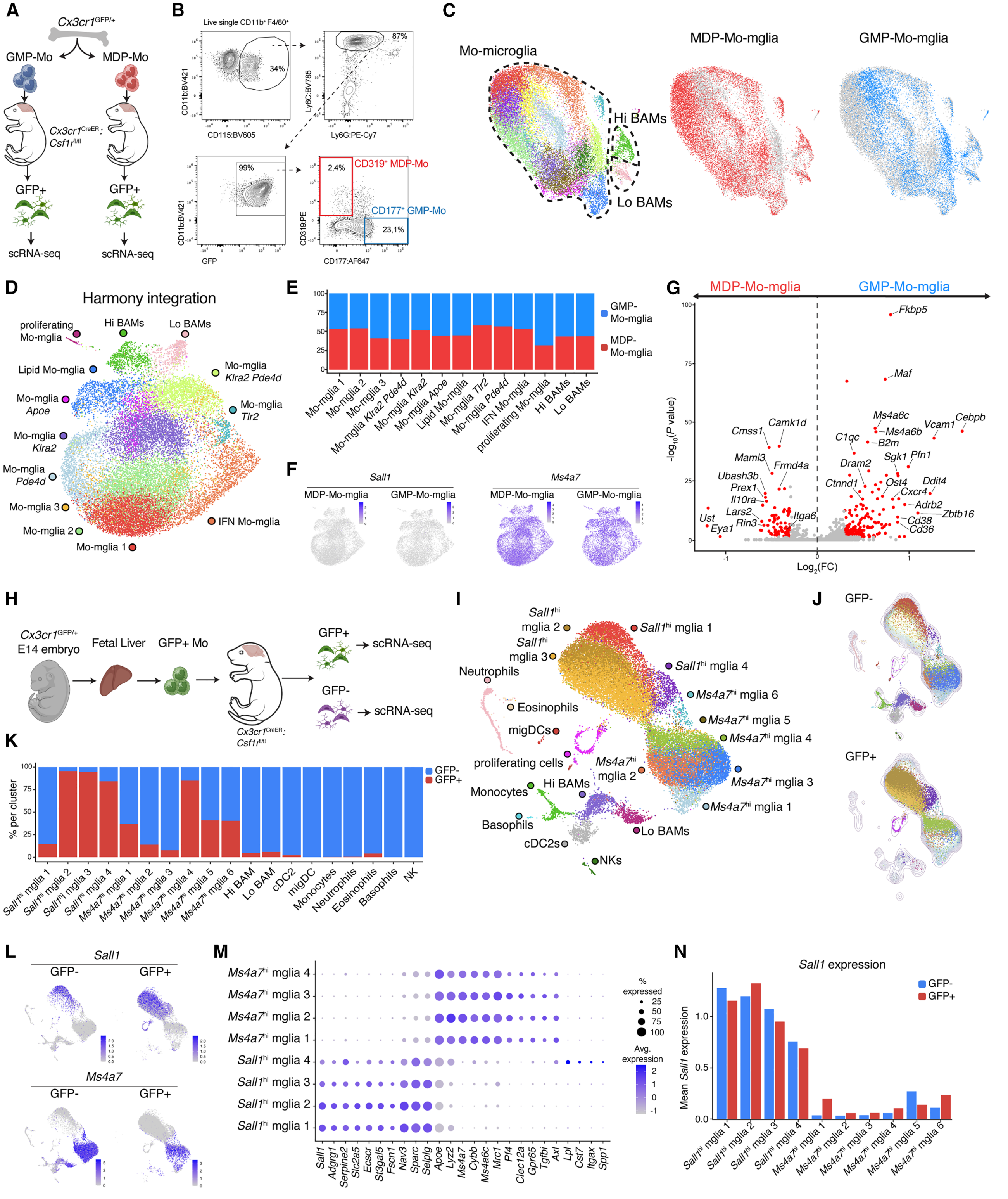

Figure 5. Monocyte ontogeny determines microglial identity

(A) scRNA-seq of MDP- and GMP-Mo-Mglia in *Cx3cr1*^{CreER}:*Csf1r*$^{fl/fl}$ mice. Detailed explanation in Figure S4O. Gating strategies in Figures S4N and S4P. *n* = 3 (MDP) and *n* = 3 (GMP), from 1 experiment.

(B) Gating strategy to FACS sort MDP- and GMP-Mo for (A). Full gating strategy in Figure S4N.

(legend continued on next page)

(Figures 5D and S4P). These states were equally represented across GMP- and MDP-derived progeny (Figure 5E). Both GMP- and MDP-Mo-Microglia exclusively exhibited a *Sall1*$^{-}$*Ms4a7*$^{+}$ profile, demonstrating that neither population expresses *Sall1* (Figure 5F). However, direct comparison revealed substantial transcriptional differences. GMP-Mo-Microglia exhibited higher expression of the transcription factors (TFs) *Maf* and *Cebpb* (Figure 5G), consistent with their upregulation in GMP-derived lung interstitial macrophages.[32] Additional DE genes included cell adhesion molecules (*Vcam1*, *Cd38*, and *Itga6*) and signaling or scavenger receptors (*Adrb2*, *Il10ra*, *Cxcr4*, and *Cd36*). Together, our findings indicate that while GMP- and MDP-derived monocytes have a similar capacity to develop into *Sall1*$^{-}$ microglia and adopt comparable cell states, they retain distinct transcriptional signatures, driven by differences in their ontogeny.

FL monocytes can develop into microglia transcriptionally equivalent to endogenous YS-derived microglia

Mouse Em-Microglia derive from YS macrophages that emerge around E7[36] and colonize the brain around E9.5. A second hematopoietic wave is initiated in the YS at E8.25, which colonizes the FL at E9.5 and result in the production of FL monocytes around E14.[36] The Em-Microglial identity may be tied to their primitive macrophage origin and may not be replicable by monocytes. To investigate this, we collected FLs from *Cx3cr1*$^{Gfp/+}$ embryos at E14 and FACS-sorted Ly6C^{hi} monocytes, as done previously[25] (Figure S4Q). FL monocytes were transplanted in *Cx3cr1*^{CreER}:*Csf1r*$^{fl/fl}$ neonates, and 8 weeks later, GFP^{+} donor and GFP^{-} host myeloid cells were separately sorted from the brain for scRNA-seq (Figures 5H and S4R–S4T). Combined analysis of GFP^{+} and GFP^{-} datasets identified various myeloid cell populations (Figures 5I, 5J, and S4U), with *Gfp* expression restricted to the GFP^{+} sorted fraction (Figure S4V). As expected, within the GFP^{-} host fraction, we identified both *Sall1*$^{+}$*Ms4a7*$^{-}$ Em-Microglia (*Sall1*^{hi} Mglia clusters) and *Sall1*$^{-}$*Ms4a7*$^{+}$ Mo-Microglia (*Ms4a7*^{hi} Mglia clusters) (Figures 5I–5M), the latter originating from endogenous host monocytes. Although the GFP^{+} fraction also harbored *Sall1*$^{-}$*Ms4a7*$^{+}$ cells, most GFP^{+} FL monocytes had developed into *Sall1*$^{+}$*Ms4a7*$^{-}$ microglia. These GFP^{+} Mo-Microglia expressed key microglial signature genes that are absent in BM-derived Mo-Microglia (e.g., *Adgrg1*, *Serpine2*, and *Slc2a5*) (Figures 5L and 5M) and their *Sall1* expression matched those of GFP^{-} Em-Microglia (Figure 5N). Additionally, they lacked BM-derived Mo-Microglia-associated genes, such as *Ms4a7*, *Clec12a*, *Ms4a6c*, and *Mrc1* (Figures 5L and 5M). Both *Sall1*^{hi} and *Ms4a7*^{hi} clusters exhibited heterogeneity, including IFN or lipid/phagocytic associated signatures (Figure S4U). Some segregation between GFP^{-} and GFP^{+} microglia was a result of sex-linked genes (Figures S4U and S4V), as the transplants were not sex matched (Figure S4V). These findings demonstrate that Em-Microglial identity is not strictly tied to a primitive macrophage origin but can also be established in fetal monocytes.

FL monocytes exhibit increased chromatin accessibility within the *Sall1* locus

EMPs, lympho-myeloid progenitors (LMPs) and HSCs coexist within the E14 FL and may contribute to monocyte output.[37–39] To explore the developmental heterogeneity of FL monocytes and their differences from BM monocytes, we FACS-sorted CD45^{+}CD11b^{+}F4/80^{+}CD115^{+} cells from E14 FL and adult BM to enrich for monocytes and their direct progenitors (Figures S5A and S5B). These were analyzed via scRNA-seq and single-nucleus chromatin accessibility profiling (snATAC-seq) (Figure 6A). In the FL, scRNA-seq revealed *Kit*$^{+}$*Cd93*$^{+}$ progenitors, including a *Ms4a3*$^{+}$*Elane*$^{+}$*Mpo*$^{+}$ cluster with a GMP identity (Figures 6B and 6C; Figure S5C). At E14, GMPs primarily derive from EMPs, with only a small fraction developing from HSCs.[40] Additionally, we identified a sizable cluster of *Flt3*$^{+}$*Il7r*$^{+}$ *Tox*$^{+}$ cells, corresponding to LMPs.[39] *Ly6c2* ^{hi}*Lyz2* ^{hi} monocytes (cluster FL Mo) could be traced back to GMPs and LMPs through various intermediate clusters, representing progressive differentiation stages. These included *Id3*$^{+}$ cells that overlapped with *Flt3*$^{+}$ LMPs (clusters FL *Id3* 1–3) and *Itga1*$^{+}$ cells that overlapped with the GMP progeny (clusters FL *Itga1* 1–2) (Figure 6C). These differentiation trajectories were also in line with predictions from RNA velocity (Figure S5D). Next, unsupervised analysis was performed for the chromatin accessibility data obtained via snATAC-seq (Figure 6D). Clusters were annotated based on mapping of the RNA-based cluster identities (Figure S5E), revealing that chromatin accessibility information alone was sufficient to distinguish GMPs, LMPs and monocytes at various stages of differentiation (Figure 6D). ChromVar was used to calculate TF enrichment based on the differential TF motif accessibility, which predicted distinct TF usage within the various progenitors and their progeny (Figure 6E).

(C) Left: UMAP of brain macrophages from both MDP-Mo and GMP-Mo conditions in (A). Right: UMAP depicting cells from the MDP-Mo or GMP-Mo conditions.
(D) UMAP of Harmony-reclustered brain macrophages from MDP-Mo and GMP-Mo conditions in (A).
(E) Percentage of cells from MDP-Mo and GMP-Mo conditions from (D).
(F) UMAP showing expression of indicated genes split per condition from (D).
(G) VP comparing DE genes between all non-proliferating MDP- and GMP-Mo-Mglia clusters in (D). ($\log_2$(FC) > 0.3, $-\log_{10}$(adjusted p value) > 0.05).
(H) Experiment for scRNA-seq of E14 FL-Mo-Mglia in *Cx3cr1*^{CreER}:*Csf1r*$^{fl/fl}$ mice. Detailed explanation in Figure S4T. Sorting strategy in Figure S4U. $n = 5$, from 1 experiment.
(I) UMAP of combined cells from GFP^{+} and GFP^{-} conditions.
(J) UMAP depicting cells from GFP^{+} or GFP^{-} conditions.
(K) Percentage of cells derived from GFP^{+} and GFP^{-} conditions per cluster in (I).
(L) UMAP showing expression of selected genes split per condition from (I).
(M) Dot plot depicting gene expression for the clusters in (I).
(N) Mean *Sall1* expression per cluster and per condition for all Mglia clusters in (I).
See also Figure S4.

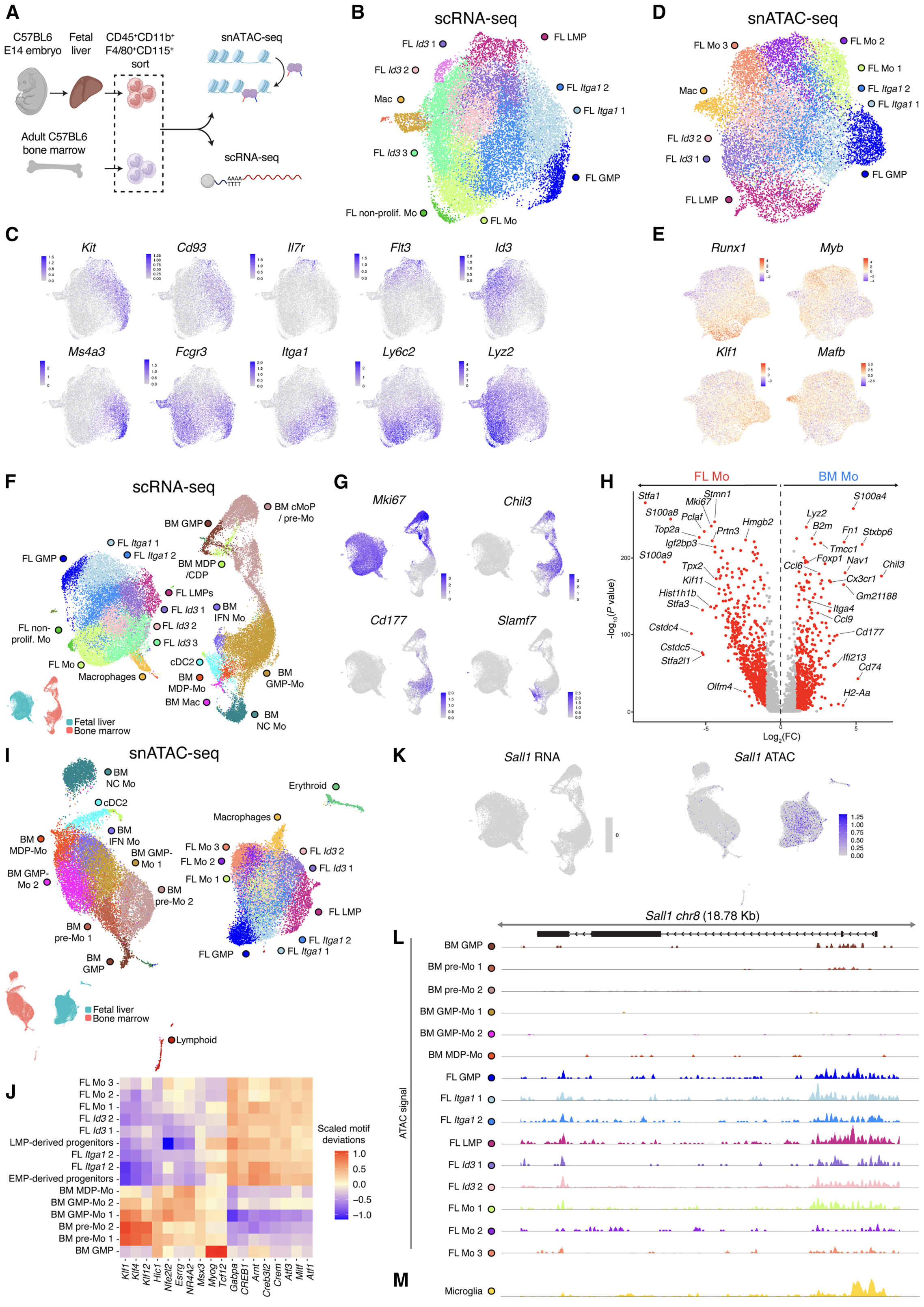

Figure 6. FL monocytes are derived from GMPs and LMPs and exhibit *Sall1* chromatin accessibility

(A) C57BL/6 E14 FL monocytes and adult BM monocytes (*n* = 3), from 1 experiment, were FACS-sorted (Figures S5A and S5B) for snATAC-seq and scRNA-seq.

(B) scRNA-seq UMAP of FL monocytes.

(legend continued on next page)

CellPress
OPEN ACCESS

Next, we combined the FL and BM scRNA-seq data and annotated the various cluster identities (Figures 6F and S5F). While the adult BM contained non-classical monocytes (*Ace* and *Treml4*), no corresponding cluster was identified in the E14 FL. Classical FL monocytes were highly distinct from their adult BM counterparts (Figures 6F–6H). Most FL monocytes exhibited an active proliferation signature (e.g., *Mki67* and *Stmn1*). Furthermore, genes that define GMP-Mo and MDP-Mo in BM monocytes, such as *Chil3*, *Cd177*, and *Slamf7/Cd319*[32] were not expressed in FL monocytes (Figure 6G). Additionally, BM monocytes showed higher expression of immune activation and chemotaxis genes (*Cx3cr1*, *Fn1*, *Ccl6*, and *Ccl9*) (Figure 6H). Chromatin accessibility analysis confirmed clear segregation between FL and BM monocytes (Figure 6I), with ChromVar predicting distinct upstream regulators (Figure 6J). While *Sall1* RNA was not detected in any dataset, there was a clear difference in chromatin accessibility in monocytes and their progenitors in FL vs. BM samples (Figure 6K). Both FL LMPs, FL GMPs, and their progeny showed ATAC signals in the *Sall1* locus, while this was absent in their BM counterparts (Figure 6L). Analysis of a public snATAC-seq dataset of mouse cortex revealed similar regions of accessible chromatin within the *Sall1* locus of cortical microglia (Figure 6M). We conclude that at E14, FL monocytes may primarily develop from LMPs and EMP-derived GMPs, in line with previous fate-mapping studies,[37,39] and exhibit increased *Sall1* accessibility, which may link to their ability to develop into *Sall1*$^+$ Mo-Microglia.

Human adult and cord blood monocytes can engraft the mouse brain as BAMs and microglia-like cells

While mouse BM monocytes fail to acquire an Em-Microglial identity, we wondered whether this also applies to their human counterparts. Xenotransplantation studies have shown that human induced pluripotent stem cell (iPSC)-derived microglial progenitors can engraft as microglia in mice that carry a human CSF1 knockin (hCSF1^{KI}), even without preconditioning.[41,42] To investigate whether human monocytes can engraft the mouse brain, CD14$^+$ classical monocytes were MACS-sorted from umbilical cord or adult blood (Figure S6A) obtained from a male and female donor per condition and intracerebrally injected into naive hCSF1^{KI}Rag2$^{-/-}$γc$^{-/-}$ neonates (Figure 7A). 4 weeks post-injection, human CD45$^+$ (hCD45$^+$) cells were detectable in the brain via flow cytometry (Figures S6B and S6C), with significantly higher engraftment in the cord blood-injected mice. On average 5.0% ± 0.9% and 2.1% ± 0.5% of brain myeloid cells were of human origin, in the cord and adult blood-injected groups, respectively (Figure S6C). In the cord blood condition, more than half of hCD45$^+$ cells were CD11b$^-$ non-myeloid cells (Figure S6C). All brain-isolated hCD45$^+$ cells were FACS-sorted for scRNA-seq analysis. Single-nucleotide polymorphism (SNP)-based demultiplexing was used to identify cells from individual donors.[43] This revealed a heterogeneous human hematopoietic compartment (Figure 7B), with cluster annotations based on known gene signatures[44] (Figure S6D). Cells from adult blood-injected mice were primarily macrophages, with a smaller fraction of monocytes and conventional type 2 dendritic cells (cDC2) (Figures 7B, 7C, S6D, and S6E). Immunostaining confirmed human IBA1$^+$ macrophages in the brain borders and parenchyma, suggesting that injected monocytes differentiated into BAMs and microglia-like cells (Figures 7D and 7E). In contrast, cord blood-injected brains contained a broader range of immune populations, including macrophages, cDC2s, cDC1s, plasmacytoid DCs, and B cells (Figures 7B, 7C, S6D, and S6E). Notably, cord-blood-injected mice also harbored hematopoietic progenitors, which were isolated and reclustered separately, revealing HSCs and their direct progeny (Figures S6F and S6G). This suggests that CD34$^+$ human cord blood HSCs, which were co-purified during MACS-based CD14$^+$ monocyte isolation (Figures S6H and S6I), had engrafted the brain and initiated local human hematopoiesis. Imaging the dura mater in cord-blood-injected mice confirmed the presence of both human IBA1$^-$ cells and human IBA1$^+$ macrophages (Figures S6J and S6K).

Monocytes and macrophages were selected and reclustered separately, revealing clear heterogeneity (Figure 7F). This included monocytes and differentiating macrophages (Transitory Mac), which appeared to branch into *FCGR3A*^{hi}*GPNMB*^{hi}*HLA-DRA*^{hi} cells (GPNMB Mac) or a *LYVE1*^{hi}*CD163*^{hi}*MRC1*^{hi} cells (*LYVE1* Mac) (Figures 7G and S6L). The latter signature relates to that of mouse MHC class II^{lo} BAMs.[2] Immunostaining confirmed the presence of human IBA1$^+$HLA-DR^{hi} and IBA1$^+$HLA-DR^{lo} macrophages within mouse dural tissue (Figure S6K). *LYVE1*$^+$ macrophages further segregated into two clusters exhibiting sex-specific differences (*LYVE1* Mac male, *LYVE1* Mac female), corresponding to either male or female donor origins (Figure S6M). Additionally, we identified a cluster that was enriched for *KCNQ3* and microglia-associated genes, including *P2RY12*, *TMEM119*, *PMEPA1*, and *CX3CR1* (cluster *KCNQ3* Mac), but lacking *SALL1*, suggesting these cells represent human Mo-Microglia (Figure 7G).

Cord blood HSCs are more primitive than adult BM HSCs and have been proposed to behave differently upon transplantation.[45] Notably, when comparing adult vs. cord-blood-derived

(C) UMAPs showing expression of selected genes.
(D) snATAC-seq UMAP of FL monocytes.
(E) UMAP showing ChromVar motif deviations of selected TFs from (D).
(F) scRNA-seq UMAP of the combined FL and BM monocytes datasets.
(G) UMAPs showing expression of selected genes.
(H) VP comparing DE genes between FL Mo ("FL Mo" and "FL non-prolif" Mo) and the combined "BM GMP-Mo, BM MDP-Mo, and BM IFN Mo" clusters in (F). (Log_2(FC) > 1, $-log_{10}$(adjusted *p* value) > 0.05).
(I) snATAC-seq UMAP of FL and BM monocytes.
(J) Heatmap of the average scaled TF motif deviations in different monocyte and progenitor subsets in (I).
(K) UMAP showing left: expression of *Sall1* RNA and right: predicted *Sall1* gene activity based on chromatin accessibility.
(L and M) Genomic tracks illustrating chromatin accessibility in the *Sall1* locus in (L) different monocyte subsets of the dataset from (I) and (M) in microglia derived from a 10× Genomics dataset.
See also Figure S5.

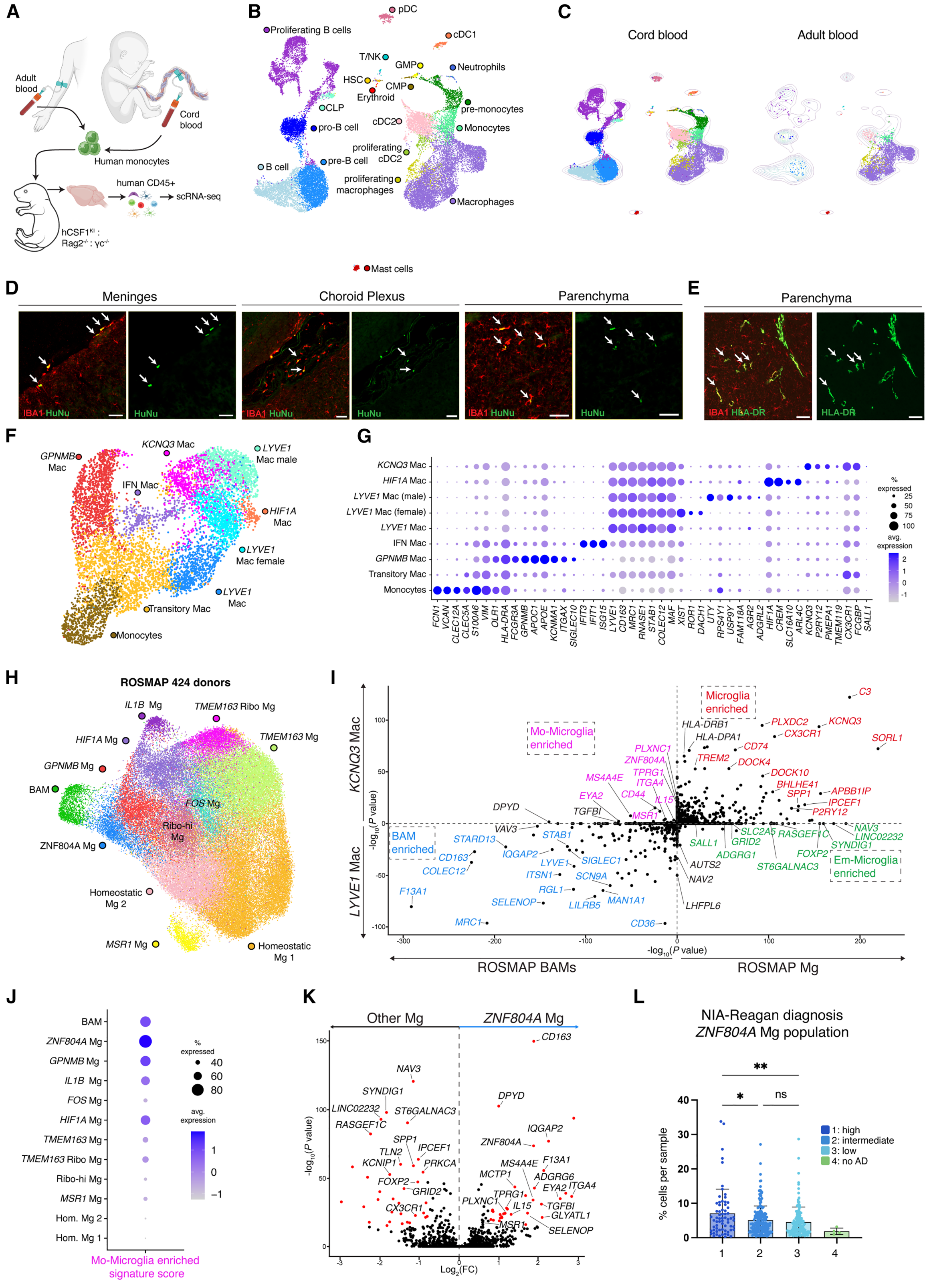

(legend on next page)

CellPress
OPEN ACCESS

cells within the *LYVE1* Mac or *KCNQ3* Mac clusters, we observed differential gene signatures (Figures S6N and S6O). Some enriched genes in cord-blood-derived cells were shared in both clusters (e.g., *SYT1* and *CHSY3*) while others were cluster specific, such as *KCNQ3* and *PLD4* in *KCNQ3* Mac and *AGR2* and *TMEM176B* in *LYVE1* Mac. Conversely, immune-related genes such as *HLA-DRB*, *LYZ*, and *CD44* were upregulated in adult-derived cells. Up- and downregulated genes were observed across both donors per condition (Figure S6P). GO analysis of DE genes revealed terms related to synaptic transmission and endocytosis for cord blood-derived cells, and terms related to inflammation and antigen presentation for the adult-blood-derived counterparts (Figures S6Q and S6R). This indicates that a cord and adult blood monocyte origin differentially impacts their cell state as brain macrophage.

Human Mo-Microglia exhibit a distinctive gene signature that can be identified in the brains of AD patients

To further validate the microglial identity of the *KCNQ3* mac cluster and compare it with human brain microglia, we analyzed brain macrophages stemming from snRNA-seq of 424 human donors collected within the Religious Orders Study and Memory and Aging Project (ROSMAP).[46] Analysis revealed multiple microglial clusters in addition to $LYVE1^{hi}CD163^{hi}MRC1^{hi}$ BAMs (Figures 7H and S7A). We plotted the DE genes of ROSMAP microglia vs. ROSMAP BAMs against the DE genes of the xenotransplanted *KCNQ3* Mac cluster vs. *LYVE1* Mac cluster for comparison (Figure 7I). This confirmed that many of the top human primary microglia-specific genes (e.g., *KCNQ3*, *C3*, *SORL1*, *CX3CR1*, *PLXDC2*, and *P2RY12*) were also enriched in the *KCNQ3* Mac cluster, while BAM-related genes (e.g., *COLEC12*, *MRC1*, and *SELENOP*) were enriched in the *LYVE1* Mac cluster (Figure 7I). This corroborates that *KCNQ3* Mac and *LYVE1* Mac reflect microglia-like and BAM-like states, respectively. Importantly, some ROSMAP microglial genes, including *SALL1*, *ADGRG1*, *SLC2A5*, and *NAV3*, were not upregulated in *KCNQ3* Mac, consistent with earlier findings in mouse Mo-Microglia (Figure 4M). This suggests that, similar to that in mice, human Mo-Microglia fail to fully replicate the Em-Microglia identity. We also noted genes that were enriched in the *KCNQ3* Mac cluster, but not in ROSMAP microglia, including *ZNF804A*, *MS4A4E*, *ITGA4*, and *EYA2*, suggestive of Mo-Microglia-enriched genes (Figure 7I). We observed that within ROSMAP microglia, cluster ZNF804A Mg exhibited an enriched expression of these Mo-Microglia related genes (Figure 7J). Comparing the ZNF804A Mg cluster with all other ROSMAP microglia, also revealed a higher expression of certain BAM-related genes (e.g., *CD163*, *TGFBI*, and *F13A1*) and a reduced expression of Em-Microglia genes (e.g., *NAV3*, *SYNDIG1*, and *FOXP2*) (Figure 7K). Thus, the *ZNF804A* Mg cluster resembles the *KCNQ3* Mac cluster, and similar to mouse Mo-Microglia, exhibits a mixed microglia-BAM signature. The proportion of the *ZNF804A* Mg cluster significantly increased with AD severity, as scored based on NIA-Reagan and Braak staging (Figures 7L, S7B, and S7C). This trend was also observed for GPNMB Mg, which exhibited a disease-associated microglia[47] signature (*GPNMB*, *MYO1E*, and *PPARG*) (Figures S7B and S7C). To assess whether a microglia cluster with a Mo-Microglia signature also emerges from an independent analysis by others, we examined Sun et al.,[48] who annotated 12 microglia clusters in a partially overlapping ROSMAP cohort (Figure S7D). Cluster Mg5, which positively correlates with amyloid and tangles,[48] exhibited the same Mo-Microglia signature (Figures S7E and S7F).

To confirm the presence of these microglia-like cells in the AD brain, we performed immunohistology in the frontal cortex of Braak VI cases, co-staining for β-amyloid, blood vessels (Ulex), Iba1, and CD163, the top DE gene in *ZNF804A* Mg. In some AD cases, $Iba1^{+}CD163^{+}$ cells were predominantly perivascular, consistent with BAMs (Figure S7G), whereas in others, $Iba1^{+}CD163^{+}$ cells were abundant in and around amyloid plaques, suggestive of parenchymal Mo-Microglia (Figure S7H). In conclusion, our findings support the notion that monocytes infiltrate the human brain during AD pathology, differentiating into Mo-Microglia that remain transcriptionally distinct from Em-Microglia.

Figure 7. Human monocytes develop into microglia-like cells within the mouse and human brain

(A) Naive hCSF1:$Rag2^{-/-}$:$\gamma c^{-/-}$ neonates were intracerebrally injected at PD4 with either cord or adult blood-derived MACS-sorted monocytes (Figure S6A). 4 weeks later, brains were isolated and $hCD45^{+}$ cells were FACS-sorted (Figure S6B). Brains were harvested from $n = 9$ (adult blood) and $n = 8$ (cord blood), from 1 experiment.
(B) UMAP of $hCD45^{+}$ cells from cord blood and adult blood conditions.
(C) UMAPs depicting cells from the cord or adult blood conditions.
(D and E) Brain sections of (D) adult blood-transplanted mice stained with anti-IBA1 and anti-human nucleus and (E) adult blood-transplanted mice stained with anti-IBA1 and anti-HLA-DR. Scale bar: 50 μm. $n = 3$ adult and $n = 3$ cord blood, from 1 experiment.
(F) UMAP of reclustered monocyte and macrophage populations from adult and cord blood conditions from (B).
(G) Dot plot depicting gene expression for the clusters annotated in (F).
(H) UMAP plot of brain macrophages originating from "ROSMAP" snRNA-seq study.
(I) Scatterplot comparing $\log_{10}$(adjusted p) values of ROSMAP Mg vs. ROSMAP BAMs (Figure 7H) on the x axis against *KCNQ3* Mac vs. *LYVE1* Mac (F) on the y axis. Mo-Microglia (magenta), Em-Microglia (green), Microglia (red), and BAM (blue) enriched genes. Only overlapping genes between the two datasets were used for analysis. p value sign given based on up or downregulation of the gene.
(J) Dot plot of the "Mo-Microglia enriched signature score" for the clusters in (H). The score shows the enrichment for genes upregulated in the *KCNQ3* vs. *LYVE1* Mac (p_val_adj < 0.05 and $\log_2 FC > 0.5$, human monocyte engraftment dataset) and downregulated in microglia vs. BAM ($\log_2 FC < 0$, ROSMAP dataset).
(K) VP comparing DE genes of the *ZNF804A* Mg cluster against all other Mg clusters from (H). ($\log_2(FC) > 0.8$, $-\log_{10}$(adjusted p value) > 15).
(L) Relative abundance of the *ZNF804A* Mg cluster in donors that were stratified based on the NIA-Reagan diagnosis score in (H). Mean ± SD.
Statistical tests: (L) Significance was determined using Tukey's multiple comparisons test where column 4 was omitted due to lack of enough datapoints to perform relevant statistical tests.
ns, *$p < 0.05$, **$p < 0.01$, ***$p < 0.001$, ****$p < 0.0001$.
See also Figures S6 and S7.

DISCUSSION

Macrophages are essential for maintaining healthy homeostasis, and their residency is accommodated by the tissue niche.[49] Macrophage loss is compensated by self-renewal or the attraction of monocytes, with their relative contribution dependent on the tissue type.[49] Microglia repopulate through self-renewal even after widespread depletion.[11,12] Additionally, microglia along with leptomeningeal and perivascular BAMs remain primarily YS-derived throughout life.[1,7] This has often been attributed to the BBB, which forms after primitive YS macrophages colonize the brain. However, we now show that despite being behind brain barriers, YS-derived BAMs are almost completely replaced by monocytes following PLX3397-induced depletion. BAMs thus behave similarly to other long-lived peripheral tissue macrophages, such as KCs.[24] One explanation is that BAMs are less proficient in self-renewal. Consistent with this, we previously reported that 10 days of repopulation following PLX3397-induced depletion, was insufficient for re-establishing leptomeningeal Lo BAM numbers.[2] This also highlights that monocyte engraftment is a slow process, not yet established 10 days after withdrawing PLX3397 treatment. We propose that similar slow engraftment kinetics apply to the parenchyma, where the rapid repopulation of YS microglia prevents monocyte engraftment. During PLX3397 administration, the niche is vacant but not engraftable, while after PLX3397 withdrawal microglia rapidly repopulate, which largely prevents monocyte engraftment. For monocytes to engraft the parenchyma as microglia, the niche must remain available and engraftable for an extended period, as we for example obtained using genetic targeting. Importantly, just as YS microglia, Mo-Microglia were long-lived cells that could undergo clonal expansion, in line with a high self-renewal capacity.

Adult monocytes are able to faithfully replicate the transcriptional state of embryonically derived peripheral macrophages including KCs and AMs[24,25] but not microglia.[15,17,21,22] CITE-seq analysis revealed many distinct Mo- vs. Em-Microglia markers, of which some may also help to differentiate Em- from Mo-Microglia during inflammation and disease, such as CLEC12A to distinguish microglia vs. monocyte-derived macrophages infiltrating brain tumors.[44] We now show that adult monocytes are also unable to replicate the phenotype of embryonically derived BAMs within the leptomeninges and perivascular spaces. Genes differentially upregulated in embryonically derived BAMs in these regions were associated with GO terms related to potential trophic functions. Recent work has shown that inflammation can also trigger the replacement of BAMs by monocytes.[50] Disease could thus lead to a functionally altered brain macrophage compartment, even after resolution. However, prolonged tissue residency may be able to eventually reprogram brain macrophages to adopt embryonic-like states, including for Mo-Microglia.[51]

Intracerebrally transplanted MDP- and GMP-monocytes developed into Mo-Microglia with distinct transcriptional signatures as has also been shown for lung IM.[32] Future experiments will need to address whether MDP- and GMP-derived Mo-Microglia show comparable niche adaptation potential, localization, and morphology. Both MDP- and GMP-Mo developed into *Sall1*$^{-}$ cells. Why are BM monocytes unable to replicate the *Sall1*$^{+}$ cell state of embryonic brain macrophages. A leading hypothesis attributes this to the fact that microglia directly originate from YS macrophages, which develop without a monocyte intermediate.[52–54] However, we now show that fetal monocytes can faithfully establish the YS microglia identity. Transcriptional and epigenetic profiling showed greater *Sall1* chromatin accessibility in E14 FL vs. adult BM monocytes. *Sall1* expression, regulated by transforming growth factor β (TGF-β)-SMAD binding,[55] may be enabled by this open chromatin state. However, a fraction of E14 fetal monocytes developed into *Sall1*$^{-}$*Ms4a7*$^{+}$ Mo-Microglia. Whether this is driven by monocyte ontogeny, developmental heterogeneity, or brain imprinting remains unclear.

Our data suggest that, as in mice, HSC-derived monocytes in humans are unable to replicate the YS microglia identity, and we provide the differential gene signature for their identification. Consistent with a recent report,[56] we identified CD163 as a Mo-Microglia marker and detected non-vessel-associated CD163^{+} macrophages in AD cortices. *Ms4a3*-based fate mapping has also revealed monocyte infiltration around amyloid plaques in mouse models of AD.[57]

Replacing dysfunctional macrophages with healthy counterparts holds promise for treating various CNS diseases. HSC transplantations have been used for treating X-linked adrenoleukodystrophy and metachromatic leukodystrophy.[58,59] Our results now show that monocytes can be used to directly replace microglia. This direct "microglia-only" replacement circumvents the high morbidity of HSC transplantation and can limit potential genetic modifications to brain macrophages, without affecting the complete hematopoietic lineage. This sets the stage for further developing cell therapies for the brain.

Limitations of the study

In this study, we only examined intravenous and intracerebral delivery of monocytes isolated from peripheral BM to the mouse brain. Future fate-mapping studies may reveal that Mo-Microglia engraftment can also arise from skull BM channels, which have direct access to the meninges and brain,[60] and that these monocytes may behave differently from peripheral monocytes entering via the circulation. Although our experiments clearly highlight transcriptional differences between embryonic and Mo-Microglia and BAMs, we only focused on tissue residency periods of 6–8 weeks. It is possible that longer residency may "erode" certain ontogeny-dependent differences in these populations, as recently suggested.[51] In the human xenotransplantation experiments, we cannot exclude the possibility that mismatches between human and mouse receptor-ligand interactions could influence the phenotype of the human Mo-Microglia and BAMs.

RESOURCE AVAILABILITY

Lead contact

Information and requests for resources should be directed to and will be fulfilled by the lead contact, Kiavash Movahedi (Kiavash.Movahedi@vub.be).

Materials availability

The *Cx3cr1*^{Flip} mice will be made freely available upon request.

Data and code availability

All scRNA-seq datasets described in this article are accessible through our interactive webserver at www.brainimmuneatlas.org, which also offers all

gene-cell count matrices and cell annotation matrices for download. All raw data and gene-cell count matrices are deposited at GEO (NCBI), with accession numbers listed in the key resources table. For human scRNA-seq data, BAM files were processed with BAMboozle[61] to safeguard donor identities by masking donor-specific SNPs and insertions or deletions (indels), replacing them with reference genome sequences. All other data that support the findings reported in this study will be shared by the lead contact upon request.

Relevant code used for scRNA-seq and snATAC-seq analysis is available at https://doi.org/10.5281/zenodo.15263274.

Any additional information required to reanalyze the data reported in this paper is available from the lead contact upon request.

ACKNOWLEDGMENTS

This work was supported by FWO (G058921N), Collen-Franqui start-up, VUB start-up (OZR3657), and ERC consolidator (101088437 ReplaceMi) grants to K.M. and an OZR VUB mandate to J.B.; and NIH grant R01-NS-120960, the Klingenstein-Simons Fellowship award in Neuroscience, and a Paul Allen Frontiers Group Distinguished Investigator award to F.C.B. We thank Jakob Van De Vaerd, Lars Feyaerts, and Geert Stangé for technical assistance; Benjamin Pavie for bioinformatical assistance (VIB Bio-Imaging Core Facility); and Wilfried Cools for advice on statistical analysis. We thank the VIB Flow Core, VIB Single Cell Core, and VIB Nucleomics Core for their support, access to RNA sequencing technologies, and use of the instrument facilities.

AUTHOR CONTRIBUTIONS

K.M., J.B., C.O'B., and F.C.B. conceived the study and designed experiments. J.B., C.O'B., M.V.-P., M.M., L.v.O., S.L., A.L.L., L.A., L.M., H.S., R.R., H.V.H., K.D.V., I.S., F.Y., S.I.L., M.B., and G.F. performed experiments. J.B., C.O'B., L. B.-C., D.K., V.V., and K.M. carried out data and bioinformatics analyses. D.G., D.G.-N., F.C.B., and K.M. provided advice on experimental design, data analyses, and interpretation. J.B. and K.M. wrote the manuscript. K.M. directed the study.

DECLARATION OF INTERESTS

F.C.B. is a co-inventor on a patent filed by The Board of Trustees of The Leland Stanford Junior University (application 16/566,675) related to methods of microglia replacement. F.C.B. holds shares in NovoGlia Inc. D.G. has been a consultant/adviser relating to Alzheimer's therapies for Merck and Novo Nordisk.

STAR★METHODS

Detailed methods are provided in the online version of this paper and include the following:

- KEY RESOURCES TABLE
- EXPERIMENTAL MODEL AND SUBJECT DETAILS
  - Mice
  - Human AD Brain tissue
- METHOD DETAILS
  - Tamoxifen administration
  - PLX3397 administration
  - Brain irradiation
  - Monocyte harvest from mouse bone marrow, mouse fetal livers, and human blood
  - Isolation of donor microglia for neonatal intracerebral transplantation
  - Monocyte intravenous injections in adults
  - Monocyte intracerebral injections in neonates
  - Brain paraformaldehyde fixation, brain cryosectioning and Immunohistochemistry
  - Adoptive monocyte transfer and engraftment in PLX3397-treated mice
  - RNA in situ hybridization (RNAScope)
  - Brain isolation, leptomeninges isolation, and digestion into single cell suspension
  - Flow cytometry and FACS
  - MDP- and GMP-monocyte isolation, sorting and intracerebral injection in neonates
  - scRNA-seq and CITE-seq using the 10× Genomics platform
  - snATAC-seq of fetal liver and BM monocytes using the 10× Genomics platform
  - Mouse scRNA-seq and CITE-seq data processing
  - 10x Genomics mouse brain snATACseq dataset processing
  - RNA Velocity analysis
  - Human Mo-Mglia scRNA-seq data processing
  - ROSMAP snRNAseq data processing
  - Mouse snATACseq data processing
  - Clonality analysis
  - Microglia morphology analysis
  - Immunofluorescence of human AD brain tissue
  - Statistics

SUPPLEMENTAL INFORMATION

Supplemental information can be found online at https://doi.org/10.1016/j.immuni.2025.04.006.

Received: July 5, 2024
Revised: January 29, 2025
Accepted: April 7, 2025
Published: April 30, 2025

REFERENCES

1. Dalmau Gasull, A., Glavan, M., Samawar, S.K.R., Kapupara, K., Kelk, J., Rubio, M., Fumagalli, S., Sorokin, L., Vivien, D., and Prinz, M. (2024). The niche matters: origin, function and fate of CNS-associated macrophages during health and disease. Acta Neuropathol. *147*, 37. https://doi.org/10.1007/s00401-023-02676-9.

2. Van Hove, H., Martens, L., Scheyltjens, I., De Vlaminck, K., Pombo Antunes, A.R., De Prijck, S., Vandamme, N., De Schepper, S., Van Isterdael, G., Scott, C.L., et al. (2019). A single-cell atlas of mouse brain macrophages reveals unique transcriptional identities shaped by ontogeny and tissue environment. Nat. Neurosci. *22*, 1021–1035. https://doi.org/10.1038/s41593-019-0393-4.

3. Mrdjen, D., Pavlovic, A., Hartmann, F.J., Schreiner, B., Utz, S.G., Leung, B. P., Lelios, I., Heppner, F.L., Kipnis, J., Merkler, D., et al. (2018). High-Dimensional Single-Cell Mapping of Central Nervous System Immune Cells Reveals Distinct Myeloid Subsets in Health, Aging, and Disease. Immunity *48*, 599. https://doi.org/10.1016/j.immuni.2018.02.014.

4. Jansen, I.E., Savage, J.E., Watanabe, K., Bryois, J., Williams, D.M., Steinberg, S., Sealock, J., Karlsson, I.K., Hägg, S., Athanasiu, L., et al. (2019). Genome-wide meta-analysis identifies new loci and functional pathways influencing Alzheimer's disease risk. Nat. Genet. *51*, 404–413. https://doi.org/10.1038/s41588-018-0311-9.

5. International Multiple Sclerosis Genetics Consortium (2019). Multiple sclerosis genomic map implicates peripheral immune cells and microglia in susceptibility. Science *365*, eaav7188. https://doi.org/10.1126/science.aav7188.

6. Bennett, M.L., and Bennett, F.C. (2020). The influence of environment and origin on brain resident macrophages and implications for therapy. Nat. Neurosci. *23*, 157–166. https://doi.org/10.1038/s41593-019-0545-6.

7. Ginhoux, F., and Guilliams, M. (2016). Tissue-Resident Macrophage Ontogeny and Homeostasis. Immunity *44*, 439–449. https://doi.org/10.1016/j.immuni.2016.02.024.

8. Masuda, T., Amann, L., Monaco, G., Sankowski, R., Staszewski, O., Krueger, M., Del Gaudio, F., He, L., Paterson, N., Nent, E., et al. (2022). Specification of CNS macrophage subsets occurs postnatally in defined

niches. Nature *604*, 740–748. https://doi.org/10.1038/s41586-022-04596-2.

9. Utz, S.G., See, P., Mildenberger, W., Thion, M.S., Silvin, A., Lutz, M., Ingelfinger, F., Rayan, N.A., Lelios, I., Buttgereit, A., et al. (2020). Early Fate Defines Microglia and Non-parenchymal Brain Macrophage Development. Cell *181*, 557–573.e18. https://doi.org/10.1016/j.cell.2020.03.021.

10. Goldmann, T., Wieghofer, P., Jordão, M.J.C., Prutek, F., Hagemeyer, N., Frenzel, K., Amann, L., Staszewski, O., Kierdorf, K., Krueger, M., et al. (2016). Origin, fate and dynamics of macrophages at central nervous system interfaces. Nat. Immunol. *17*, 797–805. https://doi.org/10.1038/ni.3423.

11. Hohsfield, L.A., Najafi, A.R., Ghorbanian, Y., Soni, N., Crapser, J., Figueroa Velez, D.X., Jiang, S., Royer, S.E., Kim, S.J., Henningfield, C. M., et al. (2021). Subventricular zone/white matter microglia reconstitute the empty adult microglial niche in a dynamic wave. eLife *10*, e66738. https://doi.org/10.7554/eLife.66738.

12. Huang, Y., Xu, Z., Xiong, S., Sun, F., Qin, G., Hu, G., Wang, J., Zhao, L., Liang, Y.X., Wu, T., et al. (2018). Repopulated microglia are solely derived from the proliferation of residual microglia after acute depletion. Nat. Neurosci. *21*, 530–540. https://doi.org/10.1038/s41593-018-0090-8.

13. Ajami, B., Bennett, J.L., Krieger, C., McNagny, K.M., and Rossi, F.M.V. (2011). Infiltrating monocytes trigger EAE progression, but do not contribute to the resident microglia pool. Nat. Neurosci. *14*, 1142–1149. https://doi.org/10.1038/nn.2887.

14. De Vlaminck, K., Van Hove, H., Kancheva, D., Scheyltjens, I., Pombo Antunes, A.R., Bastos, J., Vara-Perez, M., Ali, L., Mampay, M., Deneyer, L., et al. (2022). Differential plasticity and fate of brain-resident and recruited macrophages during the onset and resolution of neuroinflammation. Immunity *55*, 2085–2102.e9. https://doi.org/10.1016/j.immuni.2022.09.005.

15. Cronk, J.C., Filiano, A.J., Louveau, A., Marin, I., Marsh, R., Ji, E., Goldman, D.H., Smirnov, I., Geraci, N., Acton, S., et al. (2018). Peripherally derived macrophages can engraft the brain independent of irradiation and maintain an identity distinct from microglia. J. Exp. Med. *215*, 1627–1647. https://doi.org/10.1084/jem.20180247.

16. Sailor, K.A., Agoranos, G., López-Manzaneda, S., Tada, S., Gillet-Legrand, B., Guerinot, C., Masson, J.B., Vestergaard, C.L., Bonner, M., Gagnidze, K., et al. (2022). Hematopoietic stem cell transplantation chemotherapy causes microglia senescence and peripheral macrophage engraftment in the brain. Nat. Med. *28*, 517–527. https://doi.org/10.1038/s41591-022-01691-9.

17. Shemer, A., Grozovski, J., Tay, T.L., Tao, J., Volaski, A., Süß, P., Ardura-Fabregat, A., Gross-Vered, M., Kim, J.S., David, E., et al. (2018). Engrafted parenchymal brain macrophages differ from microglia in transcriptome, chromatin landscape and response to challenge. Nat. Commun. *9*, 5206. https://doi.org/10.1038/s41467-018-07548-5.

18. Shibuya, Y., Kumar, K.K., Mader, M.M.D., Yoo, Y., Ayala, L.A., Zhou, M., Mohr, M.A., Neumayer, G., Kumar, I., Yamamoto, R., et al. (2022). Treatment of a genetic brain disease by CNS-wide microglia replacement. Sci. Transl. Med. *14*, eabl9945. https://doi.org/10.1126/scitranslmed.abl9945.

19. Xu, Z., Rao, Y., Huang, Y., Zhou, T., Feng, R., Xiong, S., Yuan, T.F., Qin, S., Lu, Y., Zhou, X., et al. (2020). Efficient Strategies for Microglia Replacement in the Central Nervous System. Cell Rep. *32*, 108041. https://doi.org/10.1016/j.celrep.2020.108041.

20. Ajami, B., Bennett, J.L., Krieger, C., Tetzlaff, W., and Rossi, F.M.V. (2007). Local self-renewal can sustain CNS microglia maintenance and function throughout adult life. Nat. Neurosci. *10*, 1538–1543. https://doi.org/10.1038/nn2014.

21. Bennett, F.C., Bennett, M.L., Yaqoob, F., Mulinyawe, S.B., Grant, G.A., Hayden Gephart, M., Plowey, E.D., and Barres, B.A. (2018). A Combination of Ontogeny and CNS Environment Establishes Microglial Identity. Neuron *98*, 1170–1183.e8. https://doi.org/10.1016/j.neuron.2018.05.014.

22. Lund, H., Pieber, M., Parsa, R., Han, J., Grommisch, D., Ewing, E., Kular, L., Needhamsen, M., Espinosa, A., Nilsson, E., et al. (2018). Competitive repopulation of an empty microglial niche yields functionally distinct subsets of microglia-like cells. Nat. Commun. *9*, 4845. https://doi.org/10.1038/s41467-018-07295-7.

23. Buttgereit, A., Lelios, I., Yu, X., Vrohlings, M., Krakoski, N.R., Gautier, E.L., Nishinakamura, R., Becher, B., and Greter, M. (2016). Sall1 is a transcriptional regulator defining microglia identity and function. Nat. Immunol. *17*, 1397–1406. https://doi.org/10.1038/ni.3585.

24. Scott, C.L., Zheng, F., De Baetselier, P., Martens, L., Saeys, Y., De Prijck, S., Lippens, S., Abels, C., Schoonooghe, S., Raes, G., et al. (2016). Bone marrow-derived monocytes give rise to self-renewing and fully differentiated Kupffer cells. Nat. Commun. *7*, 10321. https://doi.org/10.1038/ncomms10321.

25. van de Laar, L., Saelens, W., De Prijck, S., Martens, L., Scott, C.L., Van Isterdael, G., Hoffmann, E., Beyaert, R., Saeys, Y., Lambrecht, B.N., et al. (2016). Yolk Sac Macrophages, Fetal Liver, and Adult Monocytes Can Colonize an Empty Niche and Develop into Functional Tissue-Resident Macrophages. Immunity *44*, 755–768. https://doi.org/10.1016/j.immuni.2016.02.017.

26. Gomez Perdiguero, E., Klapproth, K., Schulz, C., Busch, K., Azzoni, E., Crozet, L., Garner, H., Trouillet, C., de Bruijn, M.F., Geissmann, F., et al. (2015). Tissue-resident macrophages originate from yolk-sac-derived erythro-myeloid progenitors. Nature *518*, 547–551. https://doi.org/10.1038/nature13989.

27. Xu, A., Li, R., Ren, A., Jian, H., Huang, Z., Zeng, Q., Wang, B., Zheng, J., Chen, X., Zheng, N., et al. (2020). Regulatory coupling between long noncoding RNAs and senescence in irradiated microglia. J. Neuroinflammation *17*, 321. https://doi.org/10.1186/s12974-020-02001-1.

28. Oakley, H., Cole, S.L., Logan, S., Maus, E., Shao, P., Craft, J., Guillozet-Bongaarts, A., Ohno, M., Disterhoft, J., Van Eldik, L., et al. (2006). Intraneuronal beta-amyloid aggregates, neurodegeneration, and neuron loss in transgenic mice with five familial Alzheimer's disease mutations: potential factors in amyloid plaque formation. J. Neurosci. *26*, 10129–10140. https://doi.org/10.1523/JNEUROSCI.1202-06.2006.

29. Askew, K., Li, K., Olmos-Alonso, A., Garcia-Moreno, F., Liang, Y., Richardson, P., Tipton, T., Chapman, M.A., Riecken, K., Beccari, S., et al. (2017). Coupled Proliferation and Apoptosis Maintain the Rapid Turnover of Microglia in the Adult Brain. Cell Rep. *18*, 391–405. https://doi.org/10.1016/j.celrep.2016.12.041.

30. Chen, H.R., Sun, Y.Y., Chen, C.W., Kuo, Y.M., Kuan, I.S., Tiger Li, Z.R., Short-Miller, J.C., Smucker, M.R., and Kuan, C.Y. (2020). Fate mapping via CCR2-CreER mice reveals monocyte-to-microglia transition in development and neonatal stroke. Sci. Adv. *6*, eabb2119. https://doi.org/10.1126/sciadv.abb2119.

31. Barry-Carroll, L., Greulich, P., Marshall, A.R., Riecken, K., Fehse, B., Askew, K.E., Li, K., Garaschuk, O., Menassa, D.A., and Gomez-Nicola, D. (2023). Microglia colonize the developing brain by clonal expansion of highly proliferative progenitors, following allometric scaling. Cell Rep. *42*, 112425. https://doi.org/10.1016/j.celrep.2023.112425.

32. Trzebanski, S., Kim, J.S., Larossi, N., Raanan, A., Kancheva, D., Bastos, J., Haddad, M., Solomon, A., Sivan, E., Aizik, D., et al. (2024). Classical monocyte ontogeny dictates their functions and fates as tissue macrophages. Immunity *57*, 1225–1242.e6. https://doi.org/10.1016/j.immuni.2024.04.019.

33. Weinreb, C., Rodriguez-Fraticelli, A., Camargo, F.D., and Klein, A.M. (2020). Lineage tracing on transcriptional landscapes links state to fate during differentiation. Science *367*, eaaw3381. https://doi.org/10.1126/science.aaw3381.

34. Yáñez, A., Coetzee, S.G., Olsson, A., Muench, D.E., Berman, B.P., Hazelett, D.J., Salomonis, N., Grimes, H.L., and Goodridge, H.S. (2017). Granulocyte-Monocyte Progenitors and Monocyte-Dendritic Cell Progenitors Independently Produce Functionally Distinct Monocytes. Immunity *47*, 890–902.e4. https://doi.org/10.1016/j.immuni.2017.10.021.

CellPress
OPEN ACCESS

35. Korsunsky, I., Millard, N., Fan, J., Slowikowski, K., Zhang, F., Wei, K., Baglaenko, Y., Brenner, M., Loh, P.R., and Raychaudhuri, S. (2019). Fast, sensitive and accurate integration of single-cell data with Harmony. Nat. Methods *16*, 1289–1296. https://doi.org/10.1038/s41592-019-0619-0.

36. Hoeffel, G., and Ginhoux, F. (2015). Ontogeny of Tissue-Resident Macrophages. Front. Immunol. *6*, 486. https://doi.org/10.3389/fimmu.2015.00486.

37. Hoeffel, G., Chen, J., Lavin, Y., Low, D., Almeida, F.F., See, P., Beaudin, A.E., Lum, J., Low, I., Forsberg, E.C., et al. (2015). C-Myb(+) erythro-myeloid progenitor-derived fetal monocytes give rise to adult tissue-resident macrophages. Immunity *42*, 665–678. https://doi.org/10.1016/j.immuni.2015.03.011.

38. Zhang, Y., McGrath, K.E., Ayoub, E., Kingsley, P.D., Yu, H., Fegan, K., McGlynn, K.A., Rudzinskas, S., Palis, J., and Perkins, A.S. (2021). Mds1^{CreERT2}, an inducible Cre allele specific to adult-repopulating hematopoietic stem cells. Cell Rep. *36*, 109562. https://doi.org/10.1016/j.celrep.2021.109562.

39. Böiers, C., Carrelha, J., Lutteropp, M., Luc, S., Green, J.C.A., Azzoni, E., Woll, P.S., Mead, A.J., Hultquist, A., Swiers, G., et al. (2013). Lymphomyeloid contribution of an immune-restricted progenitor emerging prior to definitive hematopoietic stem cells. Cell Stem Cell *13*, 535–548. https://doi.org/10.1016/j.stem.2013.08.012.

40. Freyer, L., Iturri, L., Biton, A., Sommer, A., Dardenne, P., Cumano, A., and Perdiguero, E.G. (2021). Fetal Hematopoiesis is Driven by Privileged Expansion and Differentiation of HSC-Independent Erythro-Myeloid Progenitors. Preprint at Res. Square. https://doi.org/10.21203/rs.3.rs-1002760/v1.

41. Hasselmann, J., Coburn, M.A., England, W., Figueroa Velez, D.X., Kiani Shabestari, S., Tu, C.H., McQuade, A., Kolahdouzan, M., Echeverria, K., Claes, C., et al. (2019). Development of a Chimeric Model to Study and Manipulate Human Microglia In Vivo. Neuron *103*, 1016–1033.e10. https://doi.org/10.1016/j.neuron.2019.07.002.

42. Mancuso, R., Van Den Daele, J., Fattorelli, N., Wolfs, L., Balusu, S., Burton, O., Liston, A., Sierksma, A., Fourne, Y., Poovathingal, S., et al. (2019). Stem-cell-derived human microglia transplanted in mouse brain to study human disease. Nat. Neurosci. *22*, 2111–2116. https://doi.org/10.1038/s41593-019-0525-x.

43. Huang, Y., McCarthy, D.J., and Stegle, O. (2019). Vireo: Bayesian demultiplexing of pooled single-cell RNA-seq data without genotype reference. Genome Biol. *20*, 273. https://doi.org/10.1186/s13059-019-1865-2.

44. Pombo Antunes, A.R., Scheyltjens, I., Lodi, F., Messiaen, J., Antoranz, A., Duerinck, J., Kancheva, D., Martens, L., De Vlaminck, K., Van Hove, H., et al. (2021). Single-cell profiling of myeloid cells in glioblastoma across species and disease stage reveals macrophage competition and specialization. Nat. Neurosci. *24*, 595–610. https://doi.org/10.1038/s41593-020-00789-y.

45. Hordyjewska, A., Popiołek, Ł., and Horecka, A. (2015). Characteristics of hematopoietic stem cells of umbilical cord blood. Cytotechnology *67*, 387–396. https://doi.org/10.1007/s10616-014-9796-y.

46. Fujita, M., Gao, Z., Zeng, L., McCabe, C., White, C.C., Ng, B., Green, G.S., Rozenblatt-Rosen, O., Phillips, D., Amir-Zilberstein, L., et al. (2024). Cell subtype-specific effects of genetic variation in the Alzheimer's disease brain. Nat. Genet. *56*, 605–614. https://doi.org/10.1038/s41588-024-01685-y.

47. Keren-Shaul, H., Spinrad, A., Weiner, A., Matcovitch-Natan, O., Dvir-Szternfeld, R., Ulland, T.K., David, E., Baruch, K., Lara-Astaiso, D., Toth, B., et al. (2017). A Unique Microglia Type Associated with Restricting Development of Alzheimer's Disease. Cell *169*, 1276–1290.e17. https://doi.org/10.1016/j.cell.2017.05.018.

48. Sun, N., Victor, M.B., Park, Y.P., Xiong, X., Scannail, A.N., Leary, N., Prosper, S., Viswanathan, S., Luna, X., Boix, C.A., et al. (2023). Human microglial state dynamics in Alzheimer's disease progression. Cell *186*, 4386–4403.e29. https://doi.org/10.1016/j.cell.2023.08.037.

49. Guilliams, M., Thierry, G.R., Bonnardel, J., and Bajenoff, M. (2020). Establishment and Maintenance of the Macrophage Niche. Immunity *52*, 434–451. https://doi.org/10.1016/j.immuni.2020.02.015.

50. Wang, L., Zheng, J., Zhao, S., Wan, Y., Wang, M., Bosco, D.B., Kuan, C.Y., Richardson, J.R., and Wu, L.J. (2024). CCR2^{+} monocytes replenish border-associated macrophages in the diseased mouse brain. Cell Rep. *43*, 114120. https://doi.org/10.1016/j.celrep.2024.114120.

51. Kim, J.-S., Trzebanski, S., Shin, S.-H., Ilani, N.C., Kaushansky, N., Scheller, M., Solomon, A., Liu, Z., Aust, O., Boura-Halfon, S., et al. (2023). Monocyte-derived microglia with *Dnmt3a* mutation cause motor pathology in aging mice. Preprint at bioRxiv. https://doi.org/10.1101/2023.11.16.567402.

52. Ginhoux, F., Greter, M., Leboeuf, M., Nandi, S., See, P., Gokhan, S., Mehler, M.F., Conway, S.J., Ng, L.G., Stanley, E.R., et al. (2010). Fate mapping analysis reveals that adult microglia derive from primitive macrophages. Science *330*, 841–845. https://doi.org/10.1126/science.1194637.

53. Schulz, C., Gomez Perdiguero, E., Chorro, L., Szabo-Rogers, H., Cagnard, N., Kierdorf, K., Prinz, M., Wu, B., Jacobsen, S.E.W., Pollard, J.W., et al. (2012). A lineage of myeloid cells independent of Myb and hematopoietic stem cells. Science *336*, 86–90. https://doi.org/10.1126/science.1219179.

54. Stremmel, C., Schuchert, R., Wagner, F., Thaler, R., Weinberger, T., Pick, R., Mass, E., Ishikawa-Ankerhold, H.C., Margraf, A., Hutter, S., et al. (2018). Yolk sac macrophage progenitors traffic to the embryo during defined stages of development. Nat. Commun. *9*, 75. https://doi.org/10.1038/s41467-017-02492-2.

55. Fixsen, B.R., Han, C.Z., Zhou, Y., Spann, N.J., Saisan, P., Shen, Z., Balak, C., Sakai, M., Cobo, I., Holtman, I.R., et al. (2023). SALL1 enforces microglia-specific DNA binding and function of SMADs to establish microglia identity. Nat. Immunol. *24*, 1188–1199. https://doi.org/10.1038/s41590-023-01528-8.

56. Muñoz-Castro, C., Mejias-Ortega, M., Sanchez-Mejias, E., Navarro, V., Trujillo-Estrada, L., Jimenez, S., Garcia-Leon, J.A., Fernandez-Valenzuela, J.J., Sanchez-Mico, M.V., Romero-Molina, C., et al. (2023). Monocyte-derived cells invade brain parenchyma and amyloid plaques in human Alzheimer's disease hippocampus. Acta Neuropathol. Commun. *11*, 31. https://doi.org/10.1186/s40478-023-01530-z.

57. Silvin, A., Uderhardt, S., Piot, C., Da Mesquita, S., Yang, K., Geirsdottir, L., Mulder, K., Eyal, D., Liu, Z., Bridlance, C., et al. (2022). Dual ontogeny of disease-associated microglia and disease inflammatory macrophages in aging and neurodegeneration. Immunity *55*, 1448–1465.e6. https://doi.org/10.1016/j.immuni.2022.07.004.

58. Biffi, A., Aubourg, P., and Cartier, N. (2011). Gene therapy for leukodystrophies. Hum. Mol. Genet. *20*, R42–R53. https://doi.org/10.1093/hmg/ddr142.

59. Biffi, A., Montini, E., Lorioli, L., Cesani, M., Fumagalli, F., Plati, T., Baldoli, C., Martino, S., Calabria, A., Canale, S., et al. (2013). Lentiviral hematopoietic stem cell gene therapy benefits metachromatic leukodystrophy. Science *341*, 1233158. https://doi.org/10.1126/science.1233158.

60. Cugurra, A., Mamuladze, T., Rustenhoven, J., Dykstra, T., Beroshvili, G., Greenberg, Z.J., Baker, W., Papadopoulos, Z., Drieu, A., Blackburn, S., et al. (2021). Skull and vertebral bone marrow are myeloid cell reservoirs for the meninges and CNS parenchyma. Science *373*, eabf7844. https://doi.org/10.1126/science.abf7844.

61. Ziegenhain, C., and Sandberg, R. (2021). BAMboozle removes genetic variation from human sequence data for open data sharing. Nat. Commun. *12*, 6216. https://doi.org/10.1038/s41467-021-26152-8.

62. Li, J., Chen, K., Zhu, L., and Pollard, J.W. (2006). Conditional deletion of the colony stimulating factor-1 receptor (c-fms proto-oncogene) in mice. Genesis *44*, 328–335. https://doi.org/10.1002/dvg.20219.

63. Yona, S., Kim, K.W., Wolf, Y., Mildner, A., Varol, D., Breker, M., Strauss-Ayali, D., Viukov, S., Guilliams, M., Misharin, A., et al. (2013). Fate mapping reveals origins and dynamics of monocytes and tissue macrophages under homeostasis. Immunity *38*, 79–91. https://doi.org/10.1016/j.immuni.2012.12.001.

64. Parkhurst, C.N., Yang, G., Ninan, I., Savas, J.N., Yates, J.R., 3rd, Lafaille, J.J., Hempstead, B.L., Littman, D.R., and Gan, W.B. (2013). Microglia promote learning-dependent synapse formation through brain-derived neurotrophic factor. Cell *155*, 1596–1609. https://doi.org/10.1016/j.cell.2013.11.030.

65. Jung, S., Aliberti, J., Graemmel, P., Sunshine, M.J., Kreutzberg, G.W., Sher, A., and Littman, D.R. (2000). Analysis of fractalkine receptor CX(3)CR1 function by targeted deletion and green fluorescent protein reporter gene insertion. Mol. Cell. Biol. *20*, 4106–4114. https://doi.org/10.1128/MCB.20.11.4106-4114.2000.

66. Okabe, M., Ikawa, M., Kominami, K., Nakanishi, T., and Nishimune, Y. (1997). 'Green mice' as a source of ubiquitous green cells. FEBS Lett. *407*, 313–319. https://doi.org/10.1016/s0014-5793(97)00313-x.

67. Benz, C., Martins, V.C., Radtke, F., and Bleul, C.C. (2008). The stream of precursors that colonizes the thymus proceeds selectively through the early T lineage precursor stage of T cell development. J. Exp. Med. *205*, 1187–1199. https://doi.org/10.1084/jem.20072168.

68. Srinivas, S., Watanabe, T., Lin, C.S., William, C.M., Tanabe, Y., Jessell, T. M., and Costantini, F. (2001). Cre reporter strains produced by targeted insertion of EYFP and ECFP into the ROSA26 locus. BMC Dev. Biol. *1*, 4. https://doi.org/10.1186/1471-213x-1-4.

69. Rathinam, C., Poueymirou, W.T., Rojas, J., Murphy, A.J., Valenzuela, D. M., Yancopoulos, G.D., Rongvaux, A., Eynon, E.E., Manz, M.G., and Flavell, R.A. (2011). Efficient differentiation and function of human macrophages in humanized CSF-1 mice. Blood *118*, 3119–3128. https://doi.org/10.1182/blood-2010-12-326926.

70. Arganda-Carreras, I., Fernández-González, R., Muñoz-Barrutia, A., and Ortiz-De-Solorzano, C. (2010). 3D reconstruction of histological sections: Application to mammary gland tissue. Microsc. Res. Tech. *73*, 1019–1029. https://doi.org/10.1002/jemt.20829.

71. Schep, A.N., Wu, B., Buenrostro, J.D., and Greenleaf, W.J. (2017). chromVAR: inferring transcription-factor-associated accessibility from single-cell epigenomic data. Nat. Methods *14*, 975–978. https://doi.org/10.1038/nmeth.4401.

72. Haase, R., Royer, L.A., Steinbach, P., Schmidt, D., Dibrov, A., Schmidt, U., Weigert, M., Maghelli, N., Tomancak, P., Jug, F., et al. (2020). CLIJ: GPU-accelerated image processing for everyone. Nat. Methods *17*, 5–6. https://doi.org/10.1038/s41592-019-0650-1.

73. Lun, A.T.L., Riesenfeld, S., Andrews, T., Dao, T.P., Gomes, T.; participants in the 1st Human Cell Atlas Jamboree, and Marioni, J.C. (2019). EmptyDrops: distinguishing cells from empty droplets in droplet-based single-cell RNA sequencing data. Genome Biol. *20*, 63. https://doi.org/10.1186/s13059-019-1662-y.

74. Schindelin, J., Arganda-Carreras, I., Frise, E., Kaynig, V., Longair, M., Pietzsch, T., Preibisch, S., Rueden, C., Saalfeld, S., Schmid, B., et al. (2012). Fiji: an open-source platform for biological-image analysis. Nat. Methods *9*, 676–682. https://doi.org/10.1038/nmeth.2019.

75. Faul, F., Erdfelder, E., Lang, A.G., and Buchner, A. (2007). G*Power 3: a flexible statistical power analysis program for the social, behavioral, and biomedical sciences. Behav. Res. Methods *39*, 175–191. https://doi.org/10.3758/bf03193146.

76. Zhou, Y., Zhou, B., Pache, L., Chang, M., Khodabakhshi, A.H., Tanaseichuk, O., Benner, C., and Chanda, S.K. (2019). Metascape provides a biologist-oriented resource for the analysis of systems-level datasets. Nat. Commun. *10*, 1523. https://doi.org/10.1038/s41467-019-09234-6.

77. Legland, D., Arganda-Carreras, I., and Andrey, P. (2016). MorphoLibJ: integrated library and plugins for mathematical morphology with ImageJ. Bioinformatics *32*, 3532–3534. https://doi.org/10.1093/bioinformatics/btw413.

78. Sternberg. (1983). Biomedical Image Processing. Computer *16*, 22–34. https://doi.org/10.1109/MC.1983.1654163.

79. Satija, R., Farrell, J.A., Gennert, D., Schier, A.F., and Regev, A. (2015). Spatial reconstruction of single-cell gene expression data. Nat. Biotechnol. *33*, 495–502. https://doi.org/10.1038/nbt.3192.

80. McCarthy, D.J., Campbell, K.R., Lun, A.T.L., and Wills, Q.F. (2017). Scater: pre-processing, quality control, normalization and visualization of single-cell RNA-seq data in R. Bioinformatics *33*, 1179–1186. https://doi.org/10.1093/bioinformatics/btw777.

81. Germain, P.L., Lun, A., Garcia Meixide, C., Macnair, W., and Robinson, M. D. (2021). Doublet identification in single-cell sequencing data using scDblFinder. F1000Res *10*, 979. https://doi.org/10.12688/f1000research.73600.2.

82. Lee, T.C., Kashyap, R.L., and Chu, C.N. (1994). Building Skeleton Models via 3-D Medial Surface Axis Thinning Algorithms. CVGIP: Graph. Models Image Process. *56*, 462–478. https://doi.org/10.1006/cgip.1994.1042.

83. Fattorelli, N., Martinez-Muriana, A., Wolfs, L., Geric, I., De Strooper, B., and Mancuso, R. (2021). Stem-cell-derived human microglia transplanted into mouse brain to study human disease. Nat. Protoc. *16*, 1013–1033. https://doi.org/10.1038/s41596-020-00447-4.

84. Bankhead, P., Loughrey, M.B., Fernández, J.A., Dombrowski, Y., McArt, D.G., Dunne, P.D., McQuaid, S., Gray, R.T., Murray, L.J., Coleman, H. G., et al. (2017). QuPath: Open source software for digital pathology image analysis. Sci. Rep. *7*, 16878. https://doi.org/10.1038/s41598-017-17204-5.

85. Chadarevian, J.P., Lombroso, S.I., Peet, G.C., Hasselmann, J., Tu, C., Marzan, D.E., Capocchi, J., Purnell, F.S., Nemec, K.M., Lahian, A., et al. (2023). Engineering an inhibitor-resistant human CSF1R variant for microglia replacement. J. Exp. Med. *220*, e20220857. https://doi.org/10.1084/jem.20220857.

86. Scheyltjens, I., Van Hove, H., De Vlaminck, K., Kancheva, D., Bastos, J., Vara-Pérez, M., Pombo Antunes, A.R., Martens, L., Scott, C.L., Van Ginderachter, J.A., et al. (2022). Single-cell RNA and protein profiling of immune cells from the mouse brain and its border tissues. Nat. Protoc. *17*, 2354–2388. https://doi.org/10.1038/s41596-022-00716-4.

87. Sullivan, D.K., Min, K.H.J., Hjörleifsson, K.E., Luebbert, L., Holley, G., Moses, L., Gustafsson, J., Bray, N.L., Pimentel, H., Booeshaghi, A.S., et al. (2024). kallisto, bustools, and kb-python for quantifying bulk, single-cell, and single-nucleus RNA-seq. Preprint at bioRxiv. https://doi.org/10.1101/2023.11.21.568164.

88. Yao, Z., van Velthoven, C.T.J., Nguyen, T.N., Goldy, J., Sedeno-Cortes, A. E., Baftizadeh, F., Bertagnolli, D., Casper, T., Chiang, M., Crichton, K., et al. (2021). A taxonomy of transcriptomic cell types across the isocortex and hippocampal formation. Cell *184*, 3222–3241.e26. https://doi.org/10.1016/j.cell.2021.04.021.

89. Choi, J., Lee, W., Yoon, J.K., Choi, S.M., Lee, C.H., Moon, H.G., Cho, S., Chung, J.H., Yang, H.K., and Kim, J.I. (2022). Expression-based species deconvolution and realignment removes misalignment error in multispecies single-cell data. BMC Bioinformatics *23*, 157. https://doi.org/10.1186/s12859-022-04676-0.

90. Han, X., Zhou, Z., Fei, L., Sun, H., Wang, R., Chen, Y., Chen, H., Wang, J., Tang, H., Ge, W., et al. (2020). Construction of a human cell landscape at single-cell level. Nature *581*, 303–309. https://doi.org/10.1038/s41586-020-2157-4.

91. Han, X., Wang, R., Zhou, Y., Fei, L., Sun, H., Lai, S., Saadatpour, A., Zhou, Z., Chen, H., Ye, F., et al. (2018). Mapping the Mouse Cell Atlas by Microwell-Seq. Cell *172*, 1091–1107.e17. https://doi.org/10.1016/j.cell.2018.02.001.

92. Bennett, D.A., Buchman, A.S., Boyle, P.A., Barnes, L.L., Wilson, R.S., and Schneider, J.A. (2018). Religious Orders Study and Rush Memory and Aging Project. J. Alzheimers Dis. *64*, S161–S189. https://doi.org/10.3233/JAD-179939.

STAR★METHODS

KEY RESOURCES TABLE

REAGENT or RESOURCE	SOURCE	IDENTIFIER
**Antibodies**		
anti-mouse c-kit PE, 2B8	BioLegend	RRID: AB_313217, Cat#: 105808
anti-mouse/human CD11b PE-Cy7, M1/70	BioLegend	RRID: AB_312799, Cat#: 101216
anti-mouse/human CD11b BV421, M1/70	BioLegend	RRID: AB_2562904, Cat#: 101251
anti-mouse CD11c BV605, N418	BioLegend	RRID: AB_2562415, Cat#: 117334
anti-human CD14 V450, M5E2	BD Biosciences	RRID: AB_10611582, Cat#: 561390
anti-human CD14 BV421, M5E2	BD Biosciences	RRID: AB_2739154, Cat#: 565283
anti-human CD16 PE, 3G8	BioLegend	RRID: AB_314208, Cat#: 302008
anti-mouse CD24 AF700, M1/69	BioLegend	RRID: AB_2566730, Cat#: 101836
anti-human CD34 AF700, 561	BioLegend	RRID: AB_2632722, Cat#: 343621
anti-human CD45 PE, HI30	BioLegend	RRID: AB_314396, Cat#: 304008
anti-human CD45 AF700, HI30	Biolegend	RRID: AB_493761, Cat#: 304024
anti-mouse CD45 BV421, 30-F11	BioLegend	RRID: AB_2562559, Cat#: 103134
anti-mouse CD45 APC-Cy7, 30-F11	BioLegend	RRID: AB_312981, Cat#: 103116
anti-human CD45 V450, HI30	BD Biosciences	RRID: AB_1645573, Cat#: 560367
anti-mouse CD115 BV605, AFS98	BioLegend	RRID: AB_2562760, Cat#:135517
anti-mouse CD177 AF647, Y127	BD Biosciences	RRID: AB_2869790, Cat#: 566599
anti-mouse CD319 PE, 4G2	BioLegend	RRID: AB_2632676, Cat#: 152005
anti-mouse CLEC12A PE, 5D3/CLEC12A	BioLegend	RRID: AB_11126747, Cat#: 143403
anti-mouse CX3CR1 BV605, SA011F11	BioLegend	RRID: AB_2565937, Cat#: 149027
anti-mouse F4/80 PE-CF594, T45-2342	BD Biosciences	RRID: AB_2734770, Cat#: 565613
anti-mouse FOLR2 APC, 10/FR2	BioLegend	RRID: AB_2721313, Cat#: 153306
anti-mouse I-A/I-E PerCP-Cy5.5, M5/114.15.2	BioLegend	RRID: AB_2191071, Cat#: 107626
anti-mouse Ly6C APC, HK1.4	BioLegend	RRID: AB_1732076, Cat#: 128016
anti-mouse Ly6C BV510, HK1.4	BioLegend	RRID: AB_2562351, Cat#: 128033
anti-mouse Ly6C BV785, HK1.4	BioLegend	RRID: AB_2565852, Cat#: 128041
anti-mouse Ly6G APC, 1A8	BioLegend	RRID: AB_2227348, Cat#: 127613
anti-mouse Ly6G PE-Cy7, 1A8	BioLegend	RRID: AB_1877262, Cat#: 127617
anti-mouse MMR APC, C068C2	BioLegend	RRID: AB_10896057, Cat#: 141707
anti-mouse MMR AF700, C068C2	BioLegend	RRID: AB_2629637, Cat#: 141734
anti-mouse SiglecF AF700, 1RNM44N	Invitrogen	RRID: AB_2637126, Cat#: 56-1702-82
Chicken anti-GFP, Polyclonal	Abcam	RRID: AB_300798, Cat#: Ab13970
Rat anti-CLEC12A, 5D3/CLEC12A	BioLegend	RRID: AB_11125370, Cat#: 143402
Rabbit anti-IBA1, Polyclonal	Wako	RRID: AB_839504, Cat#: 019-19741
Goat anti-IBA1, Polyclonal	Abcam	RRID: AB_870576, Cat#: Ab48004
Goat anti-Iba1, Polyclonal	Abcam	RRID: AB_2224402, Cat#: Ab5076
Rabbit anti-RFP, Polyclonal	Abcam	RRID: AB_945213, Cat#: Ab62341
Rat anti-Lyve1, 14-0443-82	eBioscience	RRID: AB_1633414, Cat#: 14-0443-82
Goat anti-MMR, AF2535	R&D systems	RRID: AB_2063012, Cat#: AF2535
Mouse anti-human nucleus, MAB1281	Chemicon	RRID: AB_94090, Cat#: MAB1281
Rabbit anti-P2RY12, AS-55043A	Anaspec	RRID: AB_2298886, Cat#: AS-55043A
Rabbit anti-B-amyloid, 8243S	Cell Signaling Technology	RRID: AB_2797642, Cat#: 8243S
Rat anti-CD31, 550274	BD Biosciences	RRID: AB_394815, Cat#: 550274
Rabbit anti-CD163, Ab182422	Abcam	RRID: AB_2753196, Cat#: Ab182422
Rat anti-F4/80, MCA497GA	Bio-Rad	RRID: AB_323806, Cat#: MCA497GA

(*Continued on next page*)

Continued		
REAGENT or RESOURCE	SOURCE	IDENTIFIER
Goat anti-Lyve1, AF2125	R&D Systems	RRID: AB_2297188, Cat#: AF2125
Human anti-human HLA-DR, 130-122-299	Miltenyi	RRID: AB_2801880, Cat#: 130-122-299
Streptavidin-Alexa Fluor 750, S21384	Invitrogen	Cat#: S21384
Ulex-biotin, B-1065-2	Vector Laboratories	Cat#: B-1065-2
Donkey anti-rabbit Alexa Fluor 647, A31573	Invitrogen	RRID: AB_2536183, Cat#: A31573
Donkey anti-goat Alexa Fluor 647, A21447	Invitrogen	RRID: AB_141844, Cat#: A21447
Donkey anti-goat Alexa Fluor 647, A32849	Invitrogen	RRID: AB_2762840, Cat#: A32849
Donkey anti-goat Alexa Fluor 568, A11057	Invitrogen	RRID: AB_142581, Cat#: A11057
Goat anti-rat Alexa Fluor 555, A21434	Invitrogen	RRID: AB_141733, Cat#: A21434
Donkey anti-rabbit Alexa Fluor 546, A10040	Invitrogen	RRID: AB_2534016, Cat#: A10040
Donkey anti-Rabbit 594, A21207	Invitrogen	RRID: AB_141637, Cat#: A21207
Donkey anti-rat Alexa Fluor 555 plus, A48270	Invitrogen	RRID: AB_2896336, Cat#: A48270
Donkey anti-mouse Alexa Fluor 488, A21202	Invitrogen	RRID: AB_141607, Cat#: A21202
Donkey anti-chicken CF488A, SAB4600031	Merck	RRID: AB_2721061, Cat#: SAB4600031
Goat anti-chicken Alexa Fluor 488, A11039	Invitrogen	RRID: AB_142924, Cat#: A11039
Donkey anti-Rabbit Alexa Fluor 488, A21206	Invitrogen	RRID: AB_2535792, Cat#: A21206
Donkey anti-goat Alexa Fluor 488, A11055	Invitrogen	RRID: AB_2534102, Cat#: A11055
Donkey anti-human DyLight 488, SA5-10126	Invitrogen	RRID: AB_2556706, Cat#: SA5-10126
Donkey anti-goat Alexa Fluor 594, A11058	Invitrogen	RRID: AB_2534105, Cat#: A11058
Rabbit anti-B-amyloid-Alexa Fluor 488, 51374S	Cell Signaling Technology	RRID: AB_2799392, Cat#: 51374S
Chemicals, peptides, and recombinant proteins		
7-amino-actinomycin D (7-AAD)	BioLegend	Cat#: 420403
ammonium chloride	VWR	Cat#: 0621
Actinomycin D	Sigma-Aldrich	Cat#: A1410
ACK lysis buffer	Qualtiy Biological INC	Cat#: 118-156-101
Bovine serum albumin	Thermofisher	Cat#: 15260037
Citric acid	Sigma-Aldrich	Cat#: 251276
Collagenase type I	Worthington	Cat#: LS004216
Collagenase type IV	Worthington	Cat#: LS004209
Corn oil	Merck	Cat#: C8267
DAPI	Sigma-Aldrich	Cat#: D9542
DNAse I	Roche	Cat#: 04536282001
EDTA	Thermofisher	Cat#: A0436121
Fetal bovine serum	PAN-biotech	Cat#: P30-3306
Ficoll Paque Premium	Merck	Cat#: GE17-5442-02
Hanks' buffered salt solution	Gibco	Cat#: 14175053
HEPES	Gibco	Cat#: 15630
Mowiol	Polysciences Inc.	Cat#: 17951
Normal donkey serum (NDS)	Jackson Immuno	Cat#: 017-000-121
Paraformaldehyde (PFA)	Sigma-Aldrich	Cat#: 818715
Phosphate buffered saline (PBS)	Thermofisher	Cat#: 14040-133
Percoll	Cytiva	Cat#: 17-0891-01
PLX3397	Advanced ChemBlocks Inc.	Cat#: H-8970
Roswell Park Memorial Institute (RPMI)	Gibco	Cat#: 52400041
sodium hydrogen carbonate	Merck	Cat#: 106329.1000
Sucrose	Duchefa Biochemie	Cat#: S0809
Tamoxifen	Sigma-Aldrich	Cat#: T5648
Tissue-Tek O.C.T	Sakura Finetek	Cat#: 4583
Triton	Sigma-Aldrich	Cat#: X100

(*Continued on next page*)

Continued

REAGENT or RESOURCE	SOURCE	IDENTIFIER
Tween-20	Sigma-Aldrich	Cat#: P1379
Critical commercial assays		
RNAscope 2.5 Multiplex Fluorescent V2 kit	ACDBio	Cat#: 323100
MACS human classical monocyte isolation kit	Miltenyi	Cat#: 130-117-337
MACS monocyte isolation kit	Miltenyi	Cat#: 130-100-629
CD11b microglia isolation kit	Miltenyi	Cat#: 130-093-636
TSA Vivid Dyes	Tocris	Cat#: 7526 and 7527
Chromium Next Gem Single Cell 3’ Gel Bead and Library kit v3.1	10x Genomics	Cat#: 1000268
Library kit v4	10x Genomics	Cat#: 1000691
ATAC library kit v2	10x Genomics	Cat#: 1000390
Deposited data		
Raw and analyzed data	This paper	GEO: GSE292830
Experimental models: Organisms/strains		
Mouse: C57BL/6J	Janvier Labs, Charles River Labs	JAX 000664
Mouse: *Csf1r*^{Flox}	Li et al.[62]	JAX 021212
Mouse: *Cx3cr1*^{CreER}	Yona et al.[63]	JAX 020940
Mouse: *Cx3cr1*^{CreER}	Parkhurst et al.[64]	JAX 021160
Mouse: *Cx3cr1*^{GFP}	Jung et al.[65]	JAX 005582
Mouse: 5xFAD	Oakley et al.[28]	JAX 034848
Mouse: Osb-GFP	Okabe et al.[66]	JAX 006567
Mouse: *Flt3*^{Cre}	Benz et al.[67]	IMSR_EM:11790
Mouse: R26-YFP	Srinivas et al.[68]	JAX 006148
Mouse: hCSF1^{KI} x Rag2$^{-/-}$ x $\gamma c^{-/-}$	Rathinam et al.[69]	JAX 017708
Software and algorithms		
Adobe Photoshop	Adobe	RRID: SCR_014199
AnalyzeSkeleton plugin	Arganda-Carreras et al.[70]	https://github.com/fiji/AnalyzeSkeleton
BioRender app	BioRender	RRID: SCR_018361
Cell Ranger mkfastq	10x Genomics	RRID: SCR_017344
Cell Ranger count	10x Genomics	RRID: SCR_017344
chromVAR	Schep et al.[71]	RRID:SCR_026570
CLIj2 plugin	Haase et al.[72]	https://clij.github.io/
DropletUtils package	Lun et al.[73]	https://github.com/MarioniLab/DropletUtils/
Fiji (ImageJ)	Schindelin et al.[74]	RRID: SCR_002285
FlowJo	Tree Star Inc.	RRID: SCR_008520
G*Power	Faul et al.[75]	RRID: SCR_013726
GraphPad Prism	Graph Pad Software Inc.	RRID: SCR_002798
Harmony	Korsunsky et al.[35]	https://github.com/immunogenomics/harmony
Metascape	Zhou et al.[76]	RRID: SCR_016620
MorphoLibJ plugin	Legland et al.[77]	https://github.com/ijpb/MorphoLibJ
motifmatchr	Schep et al.[71]	https://greenleaflab.github.io/motifmatchr/
R studio	RStudio	RRID: SCR_000432
Rollin Ball Background Subtraction plugin	Sternberg[78]	https://github.com/nearlyfreeapps/Rolling-Ball-Algorithm
Seurat package	Satija et al.[79]	RRID: SCR_016341
Scater package	McCarthy et al.[80]	RRID: SCR_015954
scDblFinder	Germain et al.[81]	RRID: SCR_022700
Skeletonize3D plugin	Lee et al.[82]	https://github.com/fiji/Skeletonize3D
Bamboozle	Ziegenhain and Sandberg[61]	https://github.com/sandberg-lab/dataprivacy

(*Continued on next page*)

Continued		
REAGENT or RESOURCE	SOURCE	IDENTIFIER
Other		
ScRNA-seq datasets, gene-cell count matrices and cell annotation matrices	This paper	www.brainimmuneatlas.org
Code used for scRNA-seq and snATAC-seq analyses	This paper	https://doi.org/10.5281/zenodo.15263274

EXPERIMENTAL MODEL AND SUBJECT DETAILS

Mice

For experiments in Figures 2A–2D, 2G, and S2A–S2D: All animal studies were performed with approval from the Children's Hospital of Pennsylvania Institutional Animal Care and Use Committee panel in accordance with institutional and national regulations. All animals were housed in a non-barrier facility with 12-hour light-dark cycles at 23 ± 2°C. All animals were provided standard chow and water ad libitum. *Cx3cr1*^{CreER}:*Csf1r*$^{fl/fl}$ mice were generated in house by crossing *Cx3cr1*^{CreER}:B6.129P2(Cg)-$^{Cx3cr1tm2.1(cre/ERT2)Litt}$/WganJ homozygous mice (JAX 021160)[64] with *Csf1r*^{flox}:B6.Cg-Csf1r$^{tm1.2Jwp}$/J homozygous mice (JAX 021212). Experimental animals were obtained by crossing *Cx3cr1*^{CreER}:*Csf1r*$^{fl/fl}$ males with *Csf1r*$^{fl/fl}$ females such that all pups used for transplantation studies were *Cx3cr1*^{CreER}:*Csf1r*$^{fl/fl}$. Donor cells for monocyte and microglia transplants were obtained from C57BL/6-Tg(CAG-EGFP)131Osb/LeySopJ "Osb-GFP" mice (JAX 006567).[66] *Cx3cr1*^{CreER}:*Csf1r*$^{fl/fl}$ mice were also crossed to 5xFAD:B6.Cg-Tg(APPSwFlLon, PSEN1*M146L*L286V)6799Vas/Mmjax heterozygous mice (JAX 034848).[28] For 5xFAD experiments, 5–6-month-old *Cx3cr1*^{CreER}:*Csf1r*$^{fl/fl}$; 5xFAD$^{+/-}$ mice were used.

All mouse information, including their strain, age, sex, and numbers used per experiment, can be found in Table S4. C57BL6 mice were either bred in-house or purchased from Janvier Labs or Charles River Labs. Genetically altered strains were bred in-house: *Cx3cr1*^{GFP}: B6.129P2(Cg)-Cx3cr1t^{m1Litt}/J (JAX 005582),[65] *Cx3cr1*^{CreER}:B6.129P2(C)-Cx3cr1$^{tm2.1(cre/ERT2)Jung}$/J (JAX 020940),[63] *Csf1r*^{flox}:B6.Cg-Csf1r$^{tm1.2Jwp}$/J (JAX 021212),[62] R26-YFP:B6.129X1-Gt(ROSA)26Sor$^{tm1(EYFP)Cos}$/J (JAX 006148),[68] *Flt3*^{Cre}:B6.129-Tg(Flt3-cre)#Ccb/Ieg (IMSR_EM:11790),[67] *hCSF1*^{KI} x Rag2$^{-/-}$ x $\gamma c^{-/-}$:C;129S4-Rag2$^{tm1.1Flv}$ Csf1$^{tm1(CSF1)Flv}$ Il2rg$^{tm1.1Flv}$/J (JAX 017708).[69] Mice expressing the red fluorescent protein tdTomato from the endogenous *Cx3cr1* locus, called *Cx3cr1*^{Flip} mice, were generated via gene targeting in C57BL/6 ES cells. A 3 kb expression cassette was inserted into the mouse *Cx3cr1* locus replacing the endogenous ATG translational start codon. The expression cassette consisted of a tdTomato-polyA sequence in the sense direction, followed by a bicistronic sequence containing the tetracycline transactivator (tTA) protein, a T2A peptide and GFP. Both the *tTa* and *GFP* coding sequences were inserted in the antisense direction. This whole cassette was flanked by opposing lox2272 and loxN sites. In the absence of Cre recombinase, tdTomato will be expressed under the control of the endogenous *Cx3cr1* promoter. However, Cre will induce an irreversible inversion of the cassette and result in the expression of GFP and tTA. The linearized targeting vector was electroporated in ES cells, followed by neomycine selection, PCR screening, and Southern Blot confirmation. Correctly targeted ES cells were injected into C57BL6/J blastocysts to obtain chimeric mice, followed by germ line transmission and establishment of *Cx3cr1*^{Flip} strain. To obtain GFP^{+}tdTomato^{+} monocytes and Mo-Microglia, *Cx3cr1*^{GFP} and *Cx3cr1*^{Flip} mice were crossed. All mouse experiments were performed after receiving ethical approval from Ethische Commissie Dierproeven at the Vrije Universiteit Brussel. Mice were housed under standard conditions, receiving food and water *ad-libitum*. They were kept at 20°C-24°C room temperature, 45%-65% relative humidity, and a 12h day/night cycle. Daily welfare checks were performed, and a welfare log was maintained.

Human AD Brain tissue

The two postmortem brain samples used in this study were obtained from Northwestern Pathology under an Institutional Review Board (IRB) exemption. The samples were fully de-identified prior to their use, with no link to identifiable personal information, in compliance with institutional policies and applicable regulations for the use of anonymized human tissue in research. As such, no additional informed consent was required for this study. Frontal cortex tissues were sourced from the University of California at Irvine Alzheimer's Disease Research Center and Northwestern University Pathology. Patient AD1 was an 89-year-old female with an APOE genotype of 3/4 and neuropathological diagnosis of Alzheimer's disease, BRAAK tangle stage VI and CERAD stage B. Patient AD2 was an 82-year-old male with an APOE genotype of 4/4 and neuropathological diagnosis of Alzheimer's disease, BRAAK tangle stage of VI and CERAD stage 3. The study of de-identified tissues was approved by the Institutional Review Board of Northwestern University (exempt IRB #00219860). Some tissue used in the current study was supported by funding from the UCI ADRC grant NIH/NIA P30AG066519. The UCI ADRC is grateful to the donors and families who participated in the gift of brain donation.

METHOD DETAILS

Tamoxifen administration

Tamoxifen chow (250 mg/kg) was given *ad-libitum* for long-term tamoxifen treatments (Envigo, TD.130856). When a tamoxifen injection was administered, tamoxifen was given in the form of an oral gavage of 200 μl (25 mg/ml tamoxifen (T5648, Merck) in corn oil

CellPress
OPEN ACCESS

(C8267, Merck)). In neonates, tamoxifen was administered intraperitoneally: 10 μl of 10 mg/ml tamoxifen in corn oil was injected per day over two consecutive days. This happened at either PD1 and PD2 or at PD2 and PD3.

For experiments in Figures 2A–2D, 2G, and S2A–S2D: for neonatal tamoxifen administration, 100mg/kg tamoxifen (Sigma, T5648-1G) in corn oil was administered subcutaneously to *Cx3cr1*^{CreER}:*Csf1r*$^{fl/fl}$ pups on PD1 and PD2.

PLX3397 administration

PLX3397 chow was given *ad-libitum* for long-term macrophage depletions. PLX3397 molecule (Advanced Chemblock, Inc.) was mixed into AIN-76A mouse chow at 600 mg/kg (Research Diets, Inc).

Brain irradiation

All mice were irradiated using the RS 2000 X-ray Biological Irradiator (Rad Source Technologies). A 6 mm lead shield was used to protect the body of the mouse from the neck down to the tail from irradiation. Mice were anesthetized with a ketamine-xylazine (140 mg/kg Ketamidor, Ecuphar, BE-V433246 and 10 mg/kg Rompun, Bayer, 0076901) solution to restrict movement during irradiation. Afterwards, mice were irradiated with either 200, 400, 600 or 800 rad of X-ray irradiation.

Monocyte harvest from mouse bone marrow, mouse fetal livers, and human blood

Bone marrow monocytes were harvested from the tibia, femur, hips, and humerus of *Cx3cr1*$^{GFP/+}$, *Cx3cr1*$^{GFP/tdTom}$, or *Cx3cr1*$^{tdTom/+}$ mice. A cervical dislocation was performed, and the bones were dissected, cleaned, and placed in ice-cold Roswell Park Memorial Institute (RPMI) 1640 medium (Gibco, 52400041). All bones were then cut from both ends to create an open tube. A 27-gauge needle (BD, 300635)/10 ml syringe (Braun, 4616103V) was used to purge bone marrow from inside the bone using sterile 1X Hanks' buffered salt solution (HBSS, Gibco, 14175053). The bone marrow was purged above a 70 μm cell strainer (Corning, CLS431751) above a 50 ml falcon tube. The bone marrow was finely crushed using the back of a 10 ml syringe plunger in the filter cup to help dissociate it. The filter was then cleaned using 5% FACS buffer (1X HBSS (Gibco, 14175053), 2 mM EDTA (Thermofisher, A0436121), 5% (v/v) heat-inactivated fetal bovine serum (PAN-biotech, P30-3306)). Cells were centrifuged (450 g, 6 min, 4°C) and an RBC lysis buffer (155 mM ammonium chloride (VWR, 0621-500G), 12mM sodium hydrogen carbonate (Merck, 106329.1000), 0.1 mM EDTA (Thermofisher, A0436121), pH 7.4) was applied for 3-4 minutes before being neutralized with 5% FACS buffer. Cells were then centrifuged, resuspended, and counted (all in 5% FACS buffer). Afterwards, monocytes were purified using a MACS monocyte isolation kit (Miltenyi, 130-100-629). Cells were then counted again, centrifuged, and resuspended in 1X HBSS in the necessary concentrations for injections in either adults or neonates.

For experiments in Figures 2A–2D and S2A–S2D: for the isolation of bone marrow-derived monocytes, adult Osb-GFP (aged 6-12 weeks) were sacrificed, and femurs and tibia were dissected out. Whole bone marrow was then flushed out with cold 1X PBS (Invitrogen, 1133) using a 1 mL syringe, spun down in a centrifuge at 300g at 4°C, and then red blood cells were lysed with ACK lysis buffer (Quality Biological INC, 118-156-101) for 5 minutes. Bone marrow cells were then resuspended in MACS buffer (2% BSA (v/v) (Sigma Aldrich, A4161), 1 mM EDTA (Fisher Scientific, 15-575-038) in 1X PBS) for enrichment of bone marrow derived monocytes. Bone marrow monocytes were then enriched using a monocyte isolation kit following manufacturer's instructions (Miltenyi, 130-100-629). Monocytes were finally resuspended in sterile 1x PBS for transplantation.

Fetal livers were harvested from *Cx3cr1*$^{GFP/+}$ E14 embryos. Mothers were killed by cervical dislocation. The abdomen was sprayed with 70% ethanol, and the uterine horns were dissected out. These were placed in ice-cold 1X HBSS in 50 ml falcon tubes. One by one, embryos were isolated, and the fetal liver was removed and placed inside a 2 ml Eppendorf tube filled with 1 ml RPMI, with a maximum of 3 fetal livers per Eppendorf tube. The fetal livers were finely cut with spring scissors and then further mechanically dissociated by pipetting using a 1ml pipette. Cells were transferred to a 40 μm cell strainer (Greiner, 542040) above a 50 ml falcon. The filter was washed with ice-cold RPMI. Cells were centrifuged (450 g, 6 min, 4°C) and an RBC lysis buffer (155 mM ammonium chloride (VWR, 0621-500G), 12mM sodium hydrogen carbonate (Merck, 106329.1000), 0.1 mM EDTA (Thermofisher A0436121), pH 7.4) was applied for 3-4 minutes before being neutralized with 2% FACS buffer (1X HBSS (Gibco, 14175053), 2 mM EDTA (Thermofisher, A0436121), 2% (v/v) heat-inactivated fetal bovine serum (PAN-biotech, P30-3306)). Cells were centrifuged and passed through a 100 μm filter (Ferrari, PA-13-100 FG). Subsequently, cells were transferred to FACS tubes (Falcon, 352008) and stained for FACS sorting. After sorting, cells were centrifuged (450 g, 6 min, 4°C) and resuspended in 1X HBSS at the necessary concentration for intracerebral injections in neonates.

Human blood from either cord or adult origin was extracted by medical professionals from the UZ Brussel hospital after informed consent was given by the parents or adult donors, respectively, in agreement with the guidelines of the UZ Brussel Commissie Medische Ethiek. Umbilical cord blood collection was obtained by sterile puncture at the umbilical vein. This occurred after the umbilical cord was clamped 3 minutes after birth of the neonate. Cord and adult blood underwent identical procedures of purification to retrieve monocytes. Blood was diluted 1:1 with sterile room temperature 1X PBS (14040-133). 15 ml of room temperature ficoll gradient buffer (Ficoll Paque Premium, Merck, GE17-5442-02) was pipetted into a 50 ml falcon and the blood/PBS mixture was gently layered above the ficoll gradient. Samples were then centrifuged at 1000 g for 20 minutes with no brakes or acceleration. The mononuclear cell interphase was extracted using a glass Pasteur pipet. These cells were then washed with 5 ml of ice cold 1X HBSS and centrifuged (450 g, 10 min, 4°C) twice. Cells were then counted, and a human classical monocyte MACS isolation kit (Miltenyi, 130-117-337) was used following the manufacturer's instructions. After the purification, cells were counted, centrifuged (450 g, 6 min, 4°C) and resuspended in 1X HBSS at the necessary concentration for intracerebral injections in neonatal mice.

Isolation of donor microglia for neonatal intracerebral transplantation

Isolation of microglia for transplantation was performed as previously described.[21] Briefly, p7-p16 Osb-GFP pups were deeply anesthetized and intracardially perfused with 15mL cold 1x PBS. Brains were then harvested and placed in cold medium A buffer (15mM HEPES (Gibco, 15630), 0.5% glucose (Sigma, G7021), 250U/ml DNAse (Worthington Biotech, LS002007) in 1x HBSS (Gibco, 14185052). Brains were then homogenized using a cold glass dounce homogenizer (VWR, 71000-518) (3-5 pulls) and homogenized tissue was spun down at 300g at 4°C. Pellets were resuspended with 30% percoll solution (Percoll Plus, GE Healthcare, 17544501) and spun at 700g, 4°C, for 20 minutes (0 brake) to remove myelin. Following the percoll spin, top myelin layer was discarded, and cell pellets were washed twice in medium A buffer, and then resuspended in MACS buffer (2% BSA (v/v) (Sigma Aldrich, A4161), 1 mM EDTA (Fisher Scientific, 15-575-038) in 1X PBS). Microglia were then enriched using a CD11b microglia isolation kit (Miltenyi, 130-093-636) following manufacturer's instructions. Isolated microglia were then resuspended in sterile 1x PBS for transplantation. Donor microglia were intracerebrally transplanted, or a sham PBS injection given, using a glass microcapillary pulled pipette. One microliter containing $1.5x10^5$ cells was injected bilaterally into the cortex. Pups were then allowed to recover and donor cells to engraft for a few days up to 20 months before harvesting tissue.

Monocyte intravenous injections in adults

After monocyte isolation, monocytes were brought to a concentration of $2x10^6$ cells/100 μl 1X HBSS unless specified otherwise. Mice were temporarily anesthetized using isoflurane. Subsequently, a retro-orbital intravenous injection of these cells was performed. 100 μl was injected per mouse using a 0.5ml Micro Fine syringe (BD, Farmaline BE, 1319821).

Monocyte intracerebral injections in neonates

The procedure was performed as described by Fattorelli et al.[83] Briefly, after monocyte isolation, monocytes were brought to a concentration of $2.5x10^5$ cells/μl 1X HBSS (except for fetal liver monocytes which were brought to a concentration of $8x10^3$ cells/μl 1X HBSS and GMP/MDP monocytes which were injected at a concentration of $1,5x10^4$ cells/μl 1X HBSS). Neonates were injected at PD 3 or PD4, depending on when the first intraperitoneal tamoxifen injection occurred (either PD1 or PD2, respectively). $hCSF1^{KI}$ mice, which did not receive any tamoxifen, were also injected at PD3 or PD4. Neonates were cooled for 30 seconds using ice to anesthetize for a short duration. Neonates were then placed under a stereomicroscope and a small incision was made per hemisphere using a sterile scalpel at the coordinates from bregma: -1 mm anteroposterior, ± 1 mm lateral. Afterwards, 1 μl of the cell suspension was injected per hemisphere in the specific coordinates using a Hamilton syringe with a 26s gauge, 51mm long needle (Merck, 20697). Mice were then placed near a heat lamp until they regained movement, then returned to their nest. For experiments in Figures 2A–2D and S2A–S2D: Neonates were transplanted on PD3 as described in Bennett et al.[21] Donor cells (isolated as described above) were intracerebrally transplanted, or a sham PBS injection given, using a glass microcapillary pulled pipette. One microliter containing $1.5x10^5$ cells was injected bilaterally into the cortex. Pups were then allowed to recover and donor cells to engraft for a few days up to 20 months before harvesting tissue.

Brain paraformaldehyde fixation, brain cryosectioning and Immunohistochemistry

Mice were anesthetized with an overdose of the ketamine-xylazine solution (140 mg/kg Ketamidor, Ecuphar, BE-V433246 and 10 mg/kg Rompun, Bayer, 0076901). Afterwards, the ribcage was opened, and mice were transcardially perfused with 20 ml of 1X PBS followed by 20 ml of 4% paraformaldehyde (in 1X PBS) (Fisher scientific, BP531-500). Mice were decapitated and the brains were carefully isolated. Brains were then placed in 4% paraformaldehyde to post-fixate for 4 hours. These were then placed in a 15% sucrose (Duchefa Biochemie, S0809) 1X PBS solution (w/v) overnight to dehydrate on a shaker at low speed. The next day, the buffer was changed to a 30% sucrose solution and the brains were left overnight on a shaker. Brains were embedded with O.C.T (Sakura Finetek Europe) and placed at -20°C until cryosectioning. Cryosections of the brain were made using a CM3050S (Leica), where sections were 12 μm thick. Sections were placed on Superfrost Plus adhesion microscope slides (Epredia, J1800AMNZ) and frozen at -20°C until staining.

Sections were washed with 1X PBS, and then permeabilized and blocked with a 10% Normal Donkey Serum (NDS, Jackson Immuno, 017-000-121) (v/v) dilution in 0.1% (v/v) 1X PBS-Ttriton X-100 solution (Merck) for an hour. Afterwards, the primary antibodies (Table S2) were diluted in 3% NDS in 0.1% PBS-Triton X and the samples were stained overnight. The secondary antibodies (Table S2) were diluted in 3% NDS in 0.1% PBS-Triton X and samples were stained for 1.5 hours followed by washing and staining with 1:1000 dilution of 5 mg/ml DAPI (Sigma) solution in 1X PBS for 30 minutes. Samples were washed, and a coverslip was mounted using mowiol mounting medium (Polysciences, 17951-500). Brain images were taken using the PhenoCycler-Fusion system (Akoya Biosciences) or the Axioscan 7 (Zeiss). Confocal images seen in Figure 1B were taken using the LSM710 Confocal Microscope (Zeiss). Image analyses such as cell detection, quantification, and surface area calculations were performed using QuPath version 0.4.3 to 0.5.1.[84]

Quantification of area of engraftment in coronal brain sections was performed by manually annotating total brain surface area per section and afterwards identifying the surface area of the brain sections that were engrafted in QuPath. Engraftment was determined by the following parameters: presence of a GFP or YFP signal or CLEC12A signal. Engraftment percentage was then calculated as the 'engraftment surface area' which was divided by the total surface are of each brain section. An average of all slices imaged per mouse was then generated.

Spatial macrophage distribution detections were generated using QuPath. The surface of a brain section was manually annotated and the 'cell detection' tool was used to detect IBA1+ cells within a given brain section. Representative engrafted GFP+ or YFP+ IBA1+ and endogenous GFP- or YFP- IBA1+ cell detections were then manually annotated using the 'points' tool so that an object classifier could be trained to automatically classify all IBA1+ detections as either engrafted or not. All detections were then exported to a.csv file containing each detection's x and y centroid coordinates. The annotated brain perimeter outlines were exported as GeoJSON files.

For experiments in Figures 2A–2D, 2G, and S2A–S2D: following dissection, brains were drop-fixed in 4% PFA overnight at 4°C. Brains were then cryoprotected by placing in 30% sucrose solution (w/v) in 1X PBS for 2-5 days at 4°C, then frozen on dry ice in OCT blocks. 14-16um thick brain slices were cut onto Superfrost Plus slides (Fisher, 1255015) using a CM3050S (Leica) cryostat.

Sections were then blocked for one hour at room temperature using 10% donkey serum (Sigma, S30-100ml) containing 0.5% triton x-100 (Sigma-Aldrich, X-100 T8787). Primary antibodies (Table S2) were diluted in 1% donkey serum containing 0.5% triton and incubated on slides overnight at 4°C. After overnight incubation, slides were washed 3 times (5 minutes each) in 1x PBS. Secondary antibodies (Table S2) were diluted in 1% donkey serum containing 0.5% triton x-100 and incubated on slides for 1 hour at room temperature. Slides were then washed 3 times (5 minutes each) in 1x PBS. Slides were then mounted using DABCO mounting media containing DAPI for a nuclear stain (EMS, 17989-60) and coverslips sealed with quick-dry nail polish.

Stained tissue sections were imaged using a BZ-X800 fluorescent microscope (Keyence) for epifluorescence or a SP8 confocal microscope (Leica) equipped with white light laser (WLL) and diode 405 laser for confocal images. For quantification of engraftment area, 10x tile scans of 2-3 brain sections per animal were acquired. Dot-rendered images and percent area engrafted was calculated as previously described.[85] Briefly, GFP signal of donor cells was rendered into a binary image using FIJI software[74] by subtracting background and adjusting thresholding. The Analyze Particles function was then used to render GFP signal into masks. Percent area was then calculated as brain area covered by GFP signal divided by total brain section area. A similar method was used for rendering Iba1 staining into masks, where background subtraction and thresholding was based on Iba1 signal. Macrophage cell densities were calculated by first using the Polygon tool in FIJI to outline the region of interest. Following background subtraction and thresholding (the same settings applied to all samples), GFP+ or IBA1+ cells were counted using the Cell Counter tool and cell density per area recorded.

For the detection of mouse CD163 in Figure S3E, cryosectioned samples were subjected to antigen retrieval. Hereto, microscope slides were submerged in citrate buffer (10mM citric acid (Sigma, 251275) pH 6.0 and 0.05% Tween-20 (Sigma, P1379)) and heated until 95°C. Samples were kept at 95°C for 5 minutes before slowly cooling down to room temperature. Afterwards, the immunohistochemistry protocol was performed as described above.

Adoptive monocyte transfer and engraftment in PLX3397-treated mice

C57BL6 mice were given 1 week of PLX3397 chow. Afterwards, mice were transferred to regular chow. On the day of the chow switch, 2x10^6 GFP+ bone marrow monocytes were harvested (as described above) and intravenously injected per mouse (as described above) in 8-week-old C57BL6 mice. Two days later, mice were reinjected with 2x10^6 GFP+ monocytes. Four weeks later mice were sacrificed and their brains were processed for immunohistochemistry as described above.

RNA in situ hybridization (RNAScope)

For detection of mRNA in situ, the RNAscope 2.5 Multiplex Fluorescent V2 kit (ACDBio, 323100) and protocol for fixed frozen tissues was followed according to the manufacturer's instructions. TSA Vivid Dyes (Tocris, 7526 and 7527) diluted in TSA buffer (ACDBio, 322810) were used for the development of fluorescent signal. When co-staining for IBA1 or GFP, normal immunofluorescence staining protocol was followed as described above, excluding Triton x-100 in blocking and antibody dilution steps.

Brain isolation, leptomeninges isolation, and digestion into single cell suspension

Brain and leptomeningeal isolations and single-cell supsensions were performed as described previously.[2,86] Mice were anesthetized with an overdose of ketamine-xylazine solution (140 mg/kg Ketamidor, Ecuphar, BE-V433246 and 10 mg/kg Rompun, Bayer, 0076901). Afterwards, the ribcage was opened, and mice were transcardially perfused with 20 ml of 1X PBS. Mice were decapitated and the brains were isolated, sliced sagitally in half, and placed in 2 separate 2 ml Eppendorf tubes filled with 1 ml of ice-cold RPMI. To isolate leptomeninges (also called subdural meninges), a thin cortical slice of the brain was taken with a fine microtome blade, taking care to minimize parenchymal contamination as much as possible, as previously described.[86] Leptomeningeal tissue was placed in a single 1.5 ml Eppendorf tube filled with 0.5 ml RPMI. Leptomeninges samples were digested in the same way brain samples were done with only minor differences. All brain samples were then finely cut with spring scissors and subsequently 0.5 ml of enzyme digestion mix was added consisting of 30 U/ml DNAseI (Roche), 10 U/ml collagenase type I (Worthington) and 400 U/ml collagenase type IV (Worthington) diluted in 1X HBSS. Leptomeninges samples had 0.25 ml of enzyme mix added. Samples were incubated in a water bath at 37°C for 10 minutes, followed by mechanical dissociation with a 1 ml pipette, and incubation for 10 more minutes for a total of 20 minutes. Leptomeninges samples underwent a second mechanical dissociation and 10-minute incubation step for a total of 30 minutes. The solutions were resuspended until all brain/leptomeningeal tissues were fully dissociated and the samples were filtered over a 100 μm filter twice, with filters being washed with RPMI to increase cell yield. Samples were centrifuged (510 g, 7 min, 4°C) and resuspended in 30% standard isotonic Percoll (SIP, 17-0891-01, VWR) in 1X HBSS. Samples were then centrifuged at 800 g for 20 minutes with no acceleration or brakes at 4°C. The upper layer of myelin was carefully removed, and the supernatant was aspirated until the pellet was reached. The pellet was resuspended in 5ml of 2% FACS buffer (1X HBSS (Gibco,

14175053), 2 mM EDTA (Thermofisher, A0436121), 5% (v/v) heat-inactivated fetal bovine serum (PAN-biotech, P30-3306)) and the samples were centrifuged (610 g, 5 min, 4°C). The pellets were resuspended in 2% FACS buffer and were used for flow cytometry or FACS.

Flow cytometry and FACS

Brain and leptomeninges single cell suspensions were acquired as described above. Afterwards, cells were transferred to 5 ml FACS tubes for further processing. For Zombie Aqua Live-dead staining, the Zombie Aqua Fixable Viability Kit-BV510 was used (Biolegend, 423101). Cells were washed with 2% FACS buffer (1X HBSS (Gibco, 14175053), 2 mM EDTA (Thermofisher, A0436121), 5% (v/v) heat-inactivated fetal bovine serum (PAN-biotech, P30-3306)) and then centrifuged (450 g, 6 min, 4°C). The supernatant was discarded, and cells were resuspended in 100 μl of 2% FACS buffer. Fc receptors were blocked for 15 minutes: mouse Fc receptors were blocked with CD16/32 antibody (Thermofisher, 14-0161-82), human Fc receptors were blocked with the Fc block reagent from the human classical monocyte isolation kit (Miltenyi, 130-117-337). After blocking, the samples were stained with the chosen fluorescently labelled antibody mix for 20 minutes (Table S1). Samples were washed with 2 ml of 2% FACS buffer and centrifuged (450 g, 6 min, 4°C). The supernatant was discarded and the cells were resuspended in 100 μl of 2% FACS buffer. 2 minutes prior to data acquisition, 0.2 μg of DAPI / 10^6 cells was pipetted into the cell suspension to label dead cells (DAPI, Sigma, D9542). All single-stain compensations were performed using Ultracomp eBeads (Thermofisher, 01-2222-42).

Flow cytometry experiments were performed on the LSRFortessa (BD) and Aurora CS 5 laser system (Cytek). FACS sorting was performed on the FACSAria III cell sorter (BD), FACSMelody cell sorter (BD), and Aurora CS (Cytek). Analyses were performed using FlowJo v.10.

MDP- and GMP-monocyte isolation, sorting and intracerebral injection in neonates

For MDP- and GMP- monocyte intracerebral transplantations, monocytes were harvested from *Cx3cr1*$^{GFP/+}$ mice as described above. After RBC lysis, whole bone marrow was stained with DAPI and an antibody cocktail and sorted on a Cytek Aurora 5L sorter (sorting strategy found in Figure S4N). After sorting GFP$^+$ MDP and GMP monocytes, 1,5x10^4 cells were intracerebrally injected per hemisphere per brain (3x10^4 cells total) in neonatal *Cx3cr1*^{CreER}:*Csf1r*$^{fl/fl}$ mice (Intracerebral injections in neonates performed as described above). Six weeks post transplantation mice were sacrificed and their brains were processed for FACS sorting. Single cell suspensions of brains were stained with DAPI and an antibody cocktail for sorting in the Cytek Aurora 5L sorter (sorting strategy found in Figure S4P). Sorted GFP$^+$ cells we used for scRNA-seq.

scRNA-seq and CITE-seq using the 10× Genomics platform

For CITE-seq and scRNA-seq, brain and leptomeningeal isolation into single cell suspensions were performed as described above and samples were pooled to obtain sufficient number of cells. If samples were pooled, small aliquots of each sample were taken prior to pooling for assessing percentage brain engraftment via flow cytometry. Actinomycin D (ActD, Merck, A1410) powder was dissolved in sterile DMSO and added to all buffers used for the dissociation protocol to block dissociation-induced expression of immediate-early genes.[86] ActD was added in the following concentrations to all the buffers used in the tissue dissociation procedure: samples were placed in 30 μM ActD RPMI after extraction. The enzyme mix used for tissue dissociation had a concentration of 15 μM ActD. The RPMI used during the filtering procedure had 15 μM of ActD. The 30% percoll gradient step was performed using 3 μM ActD in 30% percoll, and all 2% FACS buffer that was used had 3 μM ActD. For scRNA-seq, single-cell suspensions were incubated with rat anti-mouse CD16/CD32 (Thermofisher, 14-0161-82) for 15 min to block Fc receptors and subsequently stained for 20 min, on ice. The antibodies used for staining varied over the different experiments (Table S1). For CITE-seq samples, Fc blocking and staining were simultaneously performed for 30 min on ice, in 3 μM 0.04% BSA (BSA, ThermoFisher Scientific, 15260037) in PBS (w/v) staining buffer containing mouse TruStain FcX (BioLegend, 101319), the anti-CD45-APC-cy7 antibody and a panel of 150 oligo-conjugated mouse cell surface protein antibodies as previously described[44] (Table S3). Subsequently, the samples were washed and 7-AAD (BioLegend, 420403) or DAPI (Sigma-Aldrich, D9542) were added to label dead cells prior to sorting. Cells were sorted in FBS-precoated tubes and collected in 2% FACS buffer supplemented with 3 μM ActD. Cell sorting was performed using either the Cytek Aurora CS, the BD FACSMelody cell sorter, or the BD FACSAria III cell sorter. Sorted cells were then centrifuged (450 g, 6 min, 4°C) and the pellets were resuspended in the corresponding 0.04% BSA in PBS (w/v) volume to a maximum concentration of 3529 cells/ul.

Single-cell suspensions were loaded on either a GemCode Single Cell Instrument (10x Genomics, CITEseq samples) or a Chromium X Instrument (10x Genomics, scRNAseq samples) to generate single-cell gel beads in emulsion (GEM). All scRNA-seq libraries were prepared with the Chromium Next Gem Single Cell 3' Gel Bead and Library kit v3.1 (Figures 4, 5H–5N, and 7A–7G) (10x Genomics, 1000268) or Library kit v4 (Figures 5A–5G, 6B, 6C, 6F–6H, and 6K) (10x Genomics, 1000691) according to the manufacturer's instructions. Briefly, GEM reverse-transcription incubation was performed in a 96-deep-well reaction module at 53 °C for 45 min, 85 °C for 5 min and ending at 4 °C (Library kit v3.1) and 48 °C for 45 min, 85 °C for 5 min and ending at 4 °C (Library kit v4). Next, GEMs were broken and complementary DNA (cDNA) was cleaned up using DynaBeads MyOne Silane Beads (ThermoFisher Scientific, 37002D) and SPRIselect Reagent kit (Beckman Coulter, B23318).

Full-length barcoded cDNA, originating from both the mRNA and the oligonucleotide-labeled cell surface protein antibodies, was amplified by a polymerase chain reaction (PCR) using a 96-deep-well reaction module at 98 °C for 3 min, 12-13 cycles at 98 °C for 15 s, 63 °C for 20 s and 72 °C for 1 min, 1 cycle at 72 °C for 1 min and ending at 4 °C (Library kit v3.1) and 98 °C for 45 s, 11-12 cycles at

98 °C for 20 s, 63 °C for 30 s and 72 °C for 1 min, 1 cycle at 72 °C for 1 min and ending at 4 °C (Library kit v4). Size selection via a SPRIselect Reagent kit was used to separate the amplified cDNA molecules for 3′ gene expression and cell surface protein library construction. Gene expression library construction to generate Illumina-ready sequencing libraries was performed after clean-up using SPRIselect Reagent Kit. Subsequently, Illumina-ready sequencing libraries were generated by appending to the cDNA the following sequences via enzymatic fragmentation, end-repair, A-tailing, adapter ligation, post-ligation SPRIselect cleanup/size selection and sample index PCR: R1 (read 1 primer), P5, P7, i5 and i7 sample dual index and R2 (10x Genomics). For cell surface protein library construction, separate sample index PCR and SPRIselect size selection were performed. The cDNA content of both pre-fragmentation and post-sample index PCR samples was analyzed using a High Sensitivity DNA kit (Agilent, 5067-4626) in a 2100 BioAnalyzer (Agilent). Sequencing libraries were loaded on an Illumina HiSeq4000 or an Illumina NovaSeq6000 flow cell, using the sequencing settings recommended by 10x Genomics.

snATAC-seq of fetal liver and BM monocytes using the 10× Genomics platform

For snATAC-seq of fetal liver and bone marrow, tissues were isolated as described above. No ActD was used in the buffers as the cells remained in 4°C from the dissection to the sort step. A modified protocol from the Nuclei Isolation for Single Cell ATAC Sequencing Protocol from 10x Genomics (CG000169) was used for the ATAC mouse liver and bone marrow samples. Briefly, after mouse fetal liver and bone marrow tissue digestion, FACS Abs and 7-AAD staining were used and 150 K cells were sorted on a BD FACSDiscover S8 into 1.5 mL low bind Eppendorf tube containing DPBS + 0.04% BSA as described in Figures S5A and S5B. The sorted cells were centrifuged at 400g, 4°C for 5 min. and the supernatant was discarded. Subsequently, nuclei were isolated and permeabilized by resuspending the pellet in lysis buffer (10 mM Tris-HCl pH 7.4, 10 mM NaCl, 3 mM MgCl2, 0.1% Tween-20 (Abcam, Cat. No 285432), 0.1% IGEPAL CA-630, 0.01% Digitonin (Thermo Scientific, Cat. No BN2006), 1% BSA) and incubating on ice for 3 min. After incubation, 200 μL Wash Buffer (10 mM Tris-HCl pH 7.4, 10 mM NaCl, 3 mM MgCl2, 0.1% Tween-20, 1% BSA) was added to the nuclei. The nuclei were spun down at 500g, 4°C for 5 min. The obtained nuclei pellet, after supernatant removal, was resuspended in 1x nuclei buffer (10x Genomics, Cat. No 2000207). Nuclei yield, morphology and purity was visualized using a LUNA-FL Automated cell counter (Logos biosystems).

The ATAC libraries were generated according to the manufacturer's Next GEM Single Cell ATAC protocol (10x Genomics, CG00496)). In brief, the single mouse fetal liver and bone marrow nuclei were incubated for 30 min at 37°C with a transposase (10x Genomics, Cat. No 2000265) that enters the nuclei and preferentially fragments the DNA in open regions of the chromatin and adds adapter sequences to the ends of the DNA fragments. Next, the transposed nuclei were loaded on a Chromium X Single-Cell Instrument (10x Genomics) to generate single-cell gel beads-in-emulsion (GEM). Incubation of the GEMs produced 10x Barcoded DNA from the transposed DNA. The ATAC libraries were prepared using the ATAC library kit v2 (10x Genomics, Cat. No 1000390) according to the manufacturer's instructions.

The cDNA content of the final ATAC libraries was quantified using the Qubit Flex Fluorometer (Life Technologies). The fragment size of every library was analyzed using the 5200 Fragment Analyzer (Agilent). ATAC libraries were sequenced on NovaSeq6000 instruments (Illumina) v1.5 sequencing kit S2 100 paired-end reads with sequencing settings according to the recommendations of 10x Genomics (50-8-16-50 (R1-i7-i5-R2), 1% PhiX) at VIB Nucleomics core. The generated fastq files of ATAC were processed with cellranger-atac (v2.1.0). Reads were aligned to Mus musculus reference genome (mm10-2020-A).

Mouse scRNA-seq and CITE-seq data processing

The Cell Ranger software (v.6.0.2, v.7.1.0, or v.9.0.0, 10x Genomics) was used to perform sample demultiplexing, RNA read mapping to the mm10 reference genome and RNA and ADT barcode processing, unique molecular identifiers filtering and single-cell UMI counting. The average of the mean number mapped RNA reads per cell was 29 458 ± 5 201 SE, with a mean sequencing saturation of 49.69% ± 5.59%, as calculated by Cell Ranger. The ADT libraries yielded 5593 ± 1668 mean reads per cell, and 94.1% ± 1.59% ADT sequencing saturation on average. The RNA expression matrices were further filtered and preprocessed using the Seurat[79] (v.3.2.3 and v.4.0.5) and Scater[80] (v.1.22.0) R packages. For filtering the low-quality cell barcodes, associated with droplets that do not contain intact cells, the "emptyDrops" function of the DropletUtils package[73] (v.1.14.2) has been applied on the RNA expression data, using an FDR cutoff of 0.01. Outlier cells were additionally identified based on three metrics (library size, number of expressed genes and mitochondrial proportion per cell); cells were tagged as outliers, when they were more than three median absolute deviations distant from the median value of each metric across all cells. Doublet score was assigned to each cell barcode based on generation of cluster-based artificial doublets with the scDblFinder function of the scDblFinder package[81] (1.8.0). The resulting RNA matrix was normalized using the global-scaling normalization and log-transformation 'LogNormalize' Seurat function. Highly variable genes were detected in Seurat and the gene expression was scaled by linear transformation. Subsequently, the identified highly variable genes were used for performing principal component analysis (PCA). The PCA embeddings were used downstream for unsupervised Leiden clustering of the cells and Uniform Manifold Approximation and Projection (UMAP) dimensionality reduction as implemented in Seurat. The scRNAseq dataset of CD319$^+$ (MDP-Mo) and CD177$^+$ (GMP-Mo) transplanted brains was additionally subjected to integration using Harmony v. 1.2.3. Harmony performs soft k-means clustering in the PCA space and the cluster centroids are used to compute cluster-specific linear correction factors that correct the assignment of the cluster-weighted average of each cell. Harmony was run with the theta parameter set to zero. Higher values of theta favor higher diversity of batches in the clusters and result in stronger alignment. Then, the corrected Harmony embeddings were used to perform clustering of the cells and UMAP dimensionality reduction.

Differential gene expression analysis was done using Wilcoxon Rank Sum test and Bonferroni correction has been applied for adjustment of the P values. We excluded cell populations that showed both high doublet score and expression of markers, specific for two different cell populations, e.g., of the macrophage markers C1qa/C1qb and the T cell markers Cd3e/Cd8a.

To estimate the GFP/YFP transgene expression in each dataset we further performed mapping to a modified mouse reference genome (mm10), where the transgene sequence was integrated. Subsequently, mapping to this custom-modified genome, read filtering, barcode, and unique molecular identifier (UMI) counting were performed using kb-python v.0.27.3.[87] The transgene counts were integrated into the metadata of each dataset.

The processing of the ADT expression matrix was done as described previously.[86] In brief, the ADT cell barcodes, associated with artefact cells based on the RNA expression analysis were discarded, and the remaining data was normalized using the ASINH_GEOM transformation (inverse hyperbolic sine transformation with a cofactor).

To predict potential biological functions of cell populations of interest based on differential gene expression, we performed a GO analysis using the Metascape (http://metascape.org/)[76] online tool with default parameters.

10x Genomics mouse brain snATACseq dataset processing

A public snATAC-seq dataset of fresh cortex from P50 adult mice, preprocessed with Cell Ranger ATAC 1.2.0 (2019, November 21), was downloaded from https://www.10xgenomics.com/datasets/fresh-cortex-from-adult-mouse-brain-p-50-1-standard-1-2-0. The peak-cell matrix and fragment file were imported in R and preprocessed with Seurat v.4.3.0, Signac v.1.14.0, EnsDb.Mmusculus.v79_2.99.0. The peak-cell matrix was filtered for outlier cells for the following metrics: number of reads in peaks< 3000 or >100000, the percentage of reads in peaks <40%, transcriptional start site (TSS) enrichment score <2, nucleosome banding pattern signal >4, as well as the ratio of reads in genomic blacklist regions > 0.06. Next, peak signal was normalized via the term frequency-inverse document frequency (TF-IDF) method, followed by dimensionality reduction using singular value decomposition and UMAP projection was calculated utilizing LSI components 2-30. Graph-based clustering of the ATAC data was performed using the smart local moving (SLM) algorithm and LSI components 2-30 in Seurat. Each cell was classified using the data transfer and integration method of Seurat, based on an annotated adult mouse brain scRNA-seq dataset from the Allen Brain Atlas[88] (seurat object downloaded from https://signac-objects.s3.amazonaws.com/allen_brain.rds). Then, scATAC-seq clusters were annotated based on the prediction scores for the scRNAseq clusters.

RNA Velocity analysis

RNA velocity was estimated with the velocyto.R_v.0.6 package using gene-relative model with k=25 cell kNN pooling and using top/bottom 2% quantiles for gamma fit ("fielocyto.R_" option). The RNA velocities were visualized on the UMAP embeddings via correlation-based transition probability matrix within the kNN graph using default parameters.

Human Mo-Mglia scRNA-seq data processing

The Cell Ranger software (v.7.1.0) was used to perform sample demultiplexing, RNA read mapping to the GRCh38 reference genome and barcode processing, unique molecular identifiers filtering and single-cell UMI counting. The average of the mean number mapped RNA reads per cell was 28 142 ± 2 081 SE, with a mean sequencing saturation of 47.5% ± 2.55%. The RNA expression matrices were further filtered and preprocessed as described above for the mouse scRNA-seq data processing.

In order to identify contaminating mouse or mouse-human multiplet droplets, the RNA sequencing reads were further mapped to a combined reference of the mouse mm10 and human GRCh38 genomes, using Cell Ranger. To detect mouse-human multiplets, we followed the approach from Choi et al.[89] In brief, correlation values are calculated with known cell types using the single cell Human Cell Landscape (scHCL)[90] and single cell Mouse Cell Atlas (scMCA).[91] Barcodes with high correlation to both human and mouse cells were classified as cross-species multiplets. The remaining barcodes were classified as human or mouse singlets based on the majority of reads and correlation value. The mouse singlet and cross-species multiplet barcodes were removed from further analysis.

The pooled individuals in the human Mo-Mglia scRNA-seq dataset were demultiplexed based on the single nucleotide polymorphisms (SNPs) that segregated between the samples in the pool. First, the number of alleles at each SNP in each cell were counted using cellSNP-lite (v. 1.2.1). Then, the cellSNP results were used to demultiplex the data with Vireo (v.0.2.3).[43] For the adult and the umbilical cord human monocytes, 96.84% and 92.68% of the barcodes were assigned to either of the donor individuals, respectively. The remaining barcodes were assigned as doublets or left unassigned and were discarded from further analysis.

To remove batch effects during integration of the adult and cord blood monocyte data we applied the Harmony algorithm v. 1.2.0.[35] Then, the corrected Harmony embeddings were used rather than principal components to perform unsupervised clustering of the cells and UMAP dimensionality reduction.

ROSMAP snRNAseq data processing

The ROSMAP snRNA-seq cohort has been presviously described.[46,92] All ROSMAP participants enrolled without known dementia and agreed to detailed clinical evaluation and brain donation at death [https://pubmed.ncbi.nlm.nih.gov/29865057/]. Both studies were approved by an Institutional Review Board of Rush University Medical Center (ROS IRB# L91020181, MAP IRB# L86121802). Both studies were conducted according to the principles expressed in the Declaration of Helsinki. Each participant

signed an informed consent, Anatomic Gift Act, and an RADC Repository consent (IRB# L99032481) allowing her data and biospecimens to be repurposed. ROSMAP is supported by P30AG10161, P30AG72975, R01AG15819, R01AG17917, U01AG46152, and U01AG61356.

ROSMAP resources can be requested at https://www.radc.rush.edu and www.synpase.org. For the analysis, non-macrophage cells were filtered out from the dataset, including T cells, NK cells, monocytes and unidentified cells. Further data processing was done using Seurat (v.4.0.5). The RNA matrix was normalized, highly variable genes were detected in Seurat and the gene expression was scaled by a linear transformation, followed by PCA. Next, batch correction was performed using Harmony v. 1.2.0 with theta set to zero. The harmony embeddings were used for unsupervised clustering and UMAP dimensionality reduction. Clusters, outliers for the high proportion of mitochondrial genes expressed per cell and low library size per cell were excluded from the analysis.

Mouse snATACseq data processing

The cellranger-atac-2.1.0 pipeline was used to perform sample demultiplexing and alignment of sequencing reads to the reference genome (Mus musculus mm10), barcode processing, unique molecular identifiers (UMI) filtering and the feature-barcode matrix generation for the ATAC sequencing reads. The mean raw pair reads per cell was 24 617 and 22 464, for the adult BM and the FL monocyte sample, respectively. The further pre-processing and analysis of the gene expression and ATAC count matrices was performed in R using Seurat v.4.3.0, Signac v.1.14.0, EnsDb.Mmusculus.v79_2.99.0, BSgenome.Mmusculus.UCSC.mm10 v.1.4.3, chromVAR[71] v.1.28.0, motifmatchr[71] v.1.28.0. Peak-cell matrices and fragment files generated by cellranger were imported, then the matrices were filtered for outlier cells for the following metrics: number of reads in peaks (sequencing depth measure; 2 median absolute deviations (MAD) < median), the percentage of reads in peaks (6 MAD < median), transcriptional start site (TSS) enrichment score (6 MAD < median), nucleosome banding pattern signal (6 MAD > median), as well as the ratio of reads in genomic blacklist regions (3 MAD > median), using the Scater package v.1.34.0. Next, peak signal was normalized via the term frequency-inverse document frequency (TF-IDF) method, followed by dimensionality reduction using singular value decomposition and UMAP projection was calculated utilizing LSI components 2-30. Graph-based clustering of the ATAC data was performed using the smart local moving (SLM) algorithm and LSI components 2-30 in Seurat. The gene activity matrix was calculated using the GeneActivity() function of Seurat, which sums the reads within each gene's body and promoter region (2 kb upstream). The scATACseq clusters were annotated as lymphoid, erythroid and myeloid cells based on the activity of canonical marker genes.

The FL and BM myeloid cells were subsetted and reclustered separately using LSI components 2-20 for each subset. Each cell of the respective subset was classified based on the clusters from scRNAseq data of FL or BM myeloid cells, respectively. The classification was performed using the data transfer and integration method of Seurat, which identifies pairwise correspondences between single cells across the scATACseq and scRNAseq datasets, termed "anchors", which allows transforming the datasets into a shared space, and transfer of the cluster labels between them. The anchors were identified using the canonical correlation analysis (CCA) dimensionality reduction method on the top 5000 highly variable genes from the scRNAseq datasets, and in the gene activity space of the scATACseq datasets. Then, the cluster labels were transferred to the scATACseq datasets by constructing a weights matrix that defines the association between each query cell and each anchor, using the 2-20 scATACseq LSI components and k.weigth=20 (number of neighbors for weighting). After the cluster label transfer, the scATACseq clusters were annotated based on the prediction scores for the scRNAseq clusters and the activity of specific marker genes in each cluster. Artefact clusters, identified based on higher backlist ratio signal or higher number of counts and features, were excluded from further analysis. The differential chromatin accessibility analysis was performed with the "FindMarkers" function of Seurat (Wilcoxon Rank Sum test). The p-values of differential accessibility were adjusted for multiple testing with Bonferroni correction.

Transcription factor activity was estimated on the final snATAC-seq dataset using chromVAR. Peaks with less than one read across all cells and overlapping peaks were filtered out. The M.musculus motives were obtained from the JASPAR2016 database. Then, matchMotifs() function from the motifmatchr package was used to identify which peaks contain which motifs. Then, for each set of peaks and each cell a bias corrected deviation in accessibility (ChromVar motif accessibility) was computed, using the computeDeviations() function. The "deviation" metric represents how accessible the set of peaks is relative to the expectation based on equal chromatin accessibility profiles across cells, normalized by a set of background peak sets matched for GC and average accessibility. Differential deviation signal was computed with the FindMarkers function (FDR < 0.05).

Clonality analysis

The Rstudio package SpatStat was used to analyse the spatial distribution of microglia as previously described.[31] Briefly, point patterns were generated based on the two-dimensional coordinates (XY) of each cell and the boundary coordinates of the region of interest. The 'mark' function was used to define two or more different classes of cells. The nearest neighbour distance (NND) was computed using SpatStat (command 'nndist'). The NND between cells from different classes (NND cross color) was computed using the 'nncross' functionin SpatStat. Ripley's K function (command 'Kest' in SpatStat) was used to define the spatial distribution of cells at 50 μm, 100 μm, 200 μm and 400 μm. The K function of a stationary point pattern *X* was defined that lambda *K(r)* equals the expected number of additional random points within a distance *r* of a typical random point of *X*. Here lambda is the intensity of the process, i.e. the expected number of points of *X* per unit area. The K function was determined by the second order moment properties of *X*. In order to make inferences about the spatial distribution, the estimate of *K* for the observed spatial points was compared to the true value of *K* for a Poisson point process (Theoretical), which is:

$$K(r) = \pi r^2$$

Deviations between the observed and Poisson K curves suggested spatial clustering or spatial regularity. The estimates of K(r) are of the form:

$$Kest(r) = (a / (n * (n - 1))) * sum[i,j]\, I(d[i,j] \; < \; = r)\, e[i,j])$$

where *a* refers to the area of the window, *n* is the number of data points, and the sum is taken over all ordered pairs of points *i* and *j* in X. Here *d[i,j]* is the distance between the two points, and *I(d[i,j] <= r)* is the indicator that equals 1 if the distance is less than or equal to *r*. The term *e[i,j]* is the edge correction weight that is applied to reduce bias (Ripley, 1988). The correction implemented here is Ripley's isotropic correction used for rectangular and polygonal windows (Ripley, 1988).

Points were assumed to be randomly distributed when the observed *K(r)* was equal to the theoretical *K(r)* (when K(r) Observed-Theoretical is 0). Points were considered as uniformly distributed or dispersed when the observed *K(r)* was less than the theoretical *K(r)* (when K(r) Observed-Theoretical is negative). Conversely, when the observed *K(r)* was greater than the theoretical *K(r)*, the points were considered clustered (when K(r) Observed-Theoretical is positive).

Microglia morphology analysis

3D analysis has been performed on Fiji using an ImageJ custom macro. Background subtraction has been performed using Rolling Ball Background Subtraction.[78]

Using Clij2,[72] a median filter of sigma 2 has been applied followed by an automatic threshold using the IJ-iso method. Connected component labeling box has been performed to label the different binary components, and the small ones (under 200 pixels) have been filtered out. The skeletonization has been performed using the Skeletonize3D plugin,[82] analyzed and labeled using the AnalyzeSkeleton[70] plugin to extract multiple features (Number of branches, junction, etc.). Finally, 3D Marker Controlled Watershed has been used to match the skeleton labels with the detected microglia, and intensity (Mean/Median/Max/Min/standard deviation) and shape measurements (Volume, kewness, Kurtosis, NumberOfVoxels) have been performed using the MorpholibJ plugin.[77]

Immunofluorescence of human AD brain tissue

FFPE sections (UCI-13-17, and NMA22-300) were placed in an oven at 60°C for 30 minutes before deparaffinization in xylenes and rehydration through a graded ethanol series (10 minutes in xylenes, 3 minutes in 100% ethanol twice, followed by 96%, 70%, 50%, and two washes in dH2O). Antigen retrieval was performed at 95°C for 30 minutes in Tris-EDTA buffer (pH 9.0, #AB93684, Abcam). Tissues were blocked for 2.5 hours in 10% normal donkey serum (#017-000-121, Jackson ImmunoResearch) prepared in PBS with 0.03% Triton-X (#21568-2500, Acros Organics). Primary antibodies were spun down at 4°C for 10 minutes at 4000 × g prior to use and incubated with the sections overnight at 4°C. The primary antibodies used included Ulex-biotin (1:200, #B-1065-2, Vector Labs), goat anti-Iba1 (1:100, #AB5076, Abcam), and rabbit anti-CD163 (1:200, clone EPR19518, #ab182422, Abcam). After primary antibody incubation, secondary antibodies were spun down at 4°C for 10 minutes at 4000 × g before use. Sections were incubated with Alexa Fluor-conjugated secondary antibodies (1:400) for 1 hour at room temperature. The secondary antibodies used included streptavidin-AF750 (for Ulex), donkey anti-goat AF594 (for Iba1), and donkey anti-rabbit AF647 (for CD163). Following secondary antibody incubation, slides were washed five times for 5 minutes each in PBS to remove unbound antibodies. For additional staining, sections were incubated with rabbit anti-pan-Aβ directly conjugated to AF488 (1:100, #51374S, Cell Signaling) at room temperature for 2 hours. Nuclear counterstaining was performed using DAPI (1:5000, #62248, ThermoFisher) for 15 minutes. Slides were imaged using a PhenoImager HT (Akoya Biosciences) in slide scanner mode at 40× magnification. Autofluorescence was unmixed using a library generated from tissue regions on the same slide that were processed without antibody or DAPI staining.

Statistics

Statistics on the required group size of mice for experiments were performed using G*Power 3.1.[75] Graphpad Prism v10 was used for all statistical analyses. All quantitative data was represented using mean ± standard deviation (SD). Significances were determined based on the P values being lower than 0.05 (*), 0.01 (**), 0.001 (***), and 0.0001 (****). Differential gene expression analysis was done using Wilcoxon Rank Sum test and Bonferroni correction has been applied for adjustment of the P values.

Immunity, Volume *58*

Supplemental information

Monocytes can efficiently replace all brain macrophages and fetal liver monocytes can generate bona fide SALL1^{+} microglia

Jonathan Bastos, Carleigh O'Brien, Mónica Vara-Pérez, Myrthe Mampay, Lynn van Olst, Liam Barry-Carroll, Daliya Kancheva, Sophia Leduc, Ayla Line Lievens, Leen Ali, Vladislav Vlasov, Laura Meysman, Hadis Shakeri, Ria Roelandt, Hannah Van Hove, Karen De Vlaminck, Isabelle Scheyltjens, Fazeela Yaqoob, Sonia I. Lombroso, Maria Breugelmans, Gilles Faron, Diego Gomez-Nicola, David Gate, F. Chris Bennett, and Kiavash Movahedi

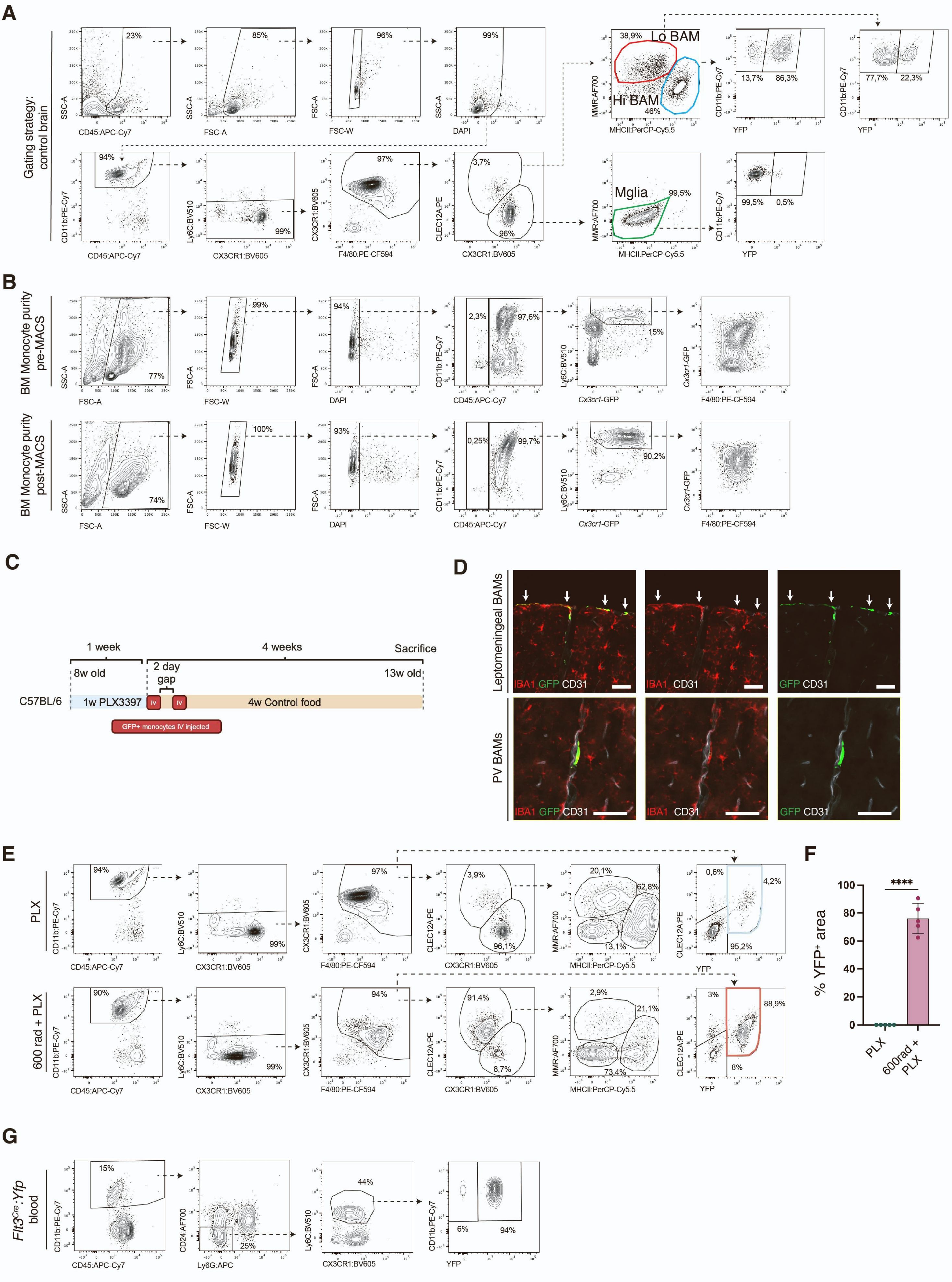

A
Gating strategy: control brain
23%
85%
96%
99%
SSC-A
CD45:APC-Cy7
FSC-A
FSC-W
DAPI
38,9%
Lo BAM
Hi BAM
46%
MMR:AF700
MHCII:PerCP-Cy5.5
13,7%
86,3%
77,7%
22,3%
CD11b:PE-Cy7
YFP
94%
99%
97%
3,7%
96%
Ly6C:BV510
CX3CR1:BV605
F4/80:PE-CF594
CLEC12A:PE
Mglia
99,5%
0,5%
B
BM Monocyte purity pre-MACS
77%
99%
94%
2,3%
97,6%
15%
BM Monocyte purity post-MACS
74%
100%
93%
0,25%
99,7%
90,2%
Cx3cr1-GFP
C
1 week
4 weeks
Sacrifice
8w old
2 day gap
13w old
C57BL/6
1w PLX3397
IV
4w Control food
GFP+ monocytes IV injected
D
Leptomeningeal BAMs
PV BAMs
IBA1 GFP CD31
IBA1 CD31
GFP CD31
E
PLX
600 rad + PLX
94%
99%
97%
3,9%
96,1%
20,1%
62,8%
13,1%
0,6%
4,2%
95,2%
90%
99%
94%
91,4%
8,7%
2,9%
21,1%
73,4%
3%
88,9%
8%
F
% YFP⁺ area
****
PLX
600rad + PLX
G
Flt3Cre:Yfp blood
15%
25%
44%
6%
94%
CD24:AF700
Ly6G:APC

Figure S1

Figure S1: Detection of YFP-labelled cells in *Flt3*^{Cre}:*Yfp* mouse brains and blood , related to Figure 1.

A) Flow cytometric gating strategy for the identification of YFP^{+} Mglia, Lo BAMs, and Hi BAMs in *Flt3*^{Cre}*:Yfp* control and depleted-repopulated brains and leptomeninges. Experimental paradigm described in Figure 1A. Data representative of control: n = 3 and PLX3397-treated: n = 3. Mice were 14-week-old males, from 2 independent experiments. **B)** Flow cytometry plots revealing the purity of *Cx3cr1*$^{\text{GFP/+}}$ monocytes from adult bone marrow before and after MACS purification, n=1 from 1 experiment. **C)** C57BL/6 mice were treated for 1 week with PLX3397 chow. Afterwards, normal chow was given for 4 weeks and on the same day mice were intravenously injected with 2 million GFP^{+} monocytes. Two days later, mice were re-injected with 2 million GFP^{+} monocytes. n = 4 C57BL/6 mice 13-week-old males, from 1 experiment. **D)** Representative images of mice in C. Slides stained with anti-IBA1 (red), anti-GFP (green), and anti-CD31 (white). Scale bar, 50 μm. **E)** Flow cytometric gating strategy to identify YFP^{+}CLEC12A^{+} Mo-Mglia engraftment in *Flt3*^{Cre}*:Yfp* male mice. Experimental paradigm described in Figure 1D. Data representative of 0 rad: n = 5, 200 rad: n = 6, 400 rad: n = 7, 600 rad: n = 6, 800 rad: n = 6. Mice were 10- to 16-week-old males, from 2 independent experiments. Cells were pre-gated as CD45^{+} live single cells as shown in A. **F)** Surface area analysis of brain cryosections derived from Figure 1F. Brain surface area of YFP^{+} Mo-Mglia was quantified. n = 5 PLX and n = 5 600 rad + PLX, 10-week-old males, from 2 independent experiments. Mean ± SD. Significance was determined using an unpaired two-tailed *t*-test. ns, *p < 0.05, **p < 0.01, ***p < 0.001, ****p < 0.0001 **G)** Gating strategy to identify YFP^{+} monocytes in *Flt3*^{Cre}*:Yfp* mouse blood. Cells were pre-gated as CD45^{+}, single, live cells. n=4, from 1 experiment.

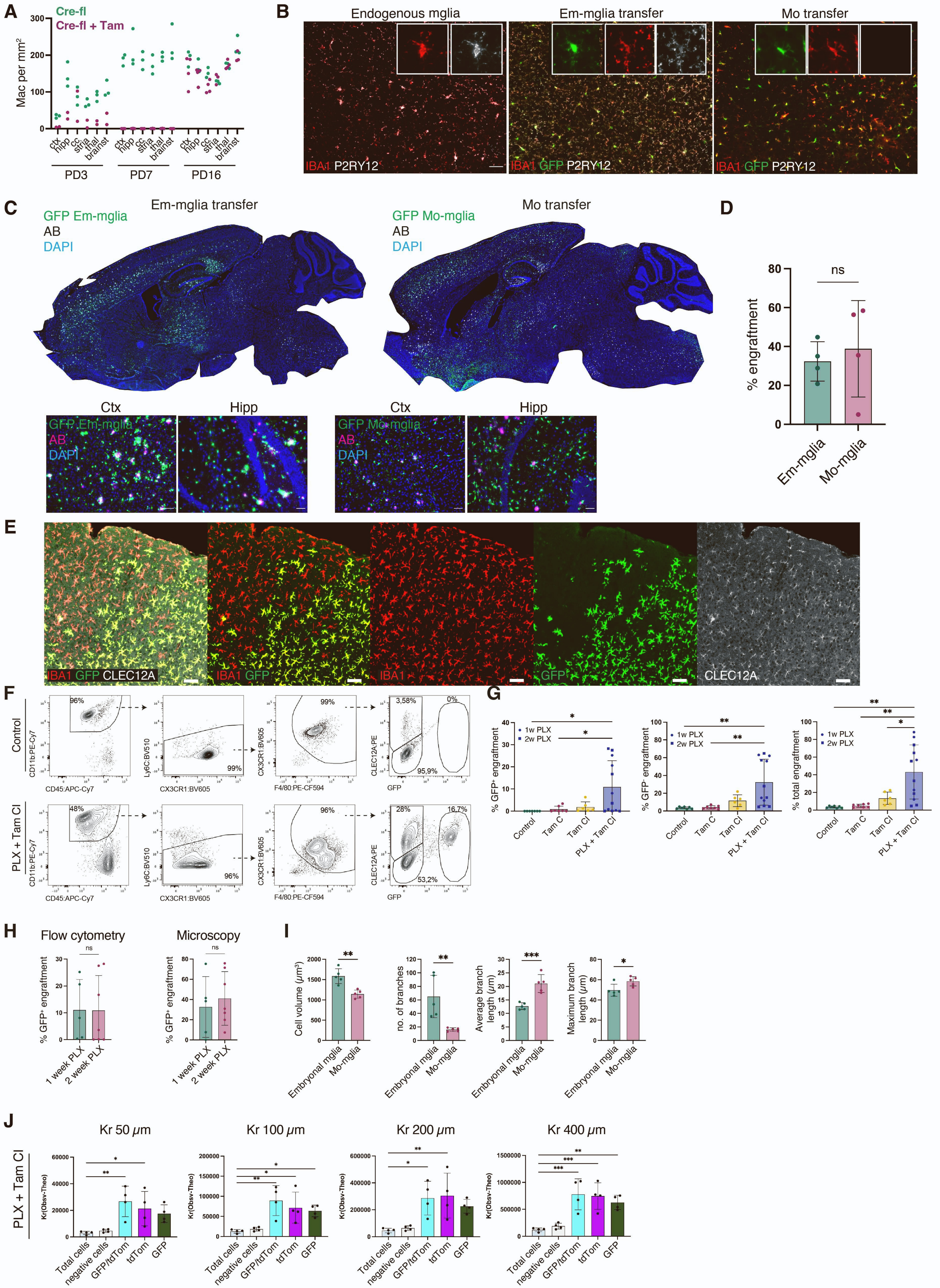
A
Cre-fl
Cre-fl + Tam
Mac per mm²
ctx hipp cc stria thal brainst
PD3
PD7
PD16
B
Endogenous mglia
Em-mglia transfer
Mo transfer
IBA1 P2RY12
IBA1 GFP P2RY12
C
Em-mglia transfer
GFP Em-mglia
AB
DAPI
Mo transfer
GFP Mo-mglia
Ctx
Hipp
D
ns
% engraftment
Em-mglia
Mo-mglia
E
IBA1 GFP CLEC12A
IBA1 GFP
IBA1
GFP
CLEC12A
F
Control
PLX + Tam CI
CD11b:PE-Cy7
CD45:APC-Cy7
Ly6C:BV510
CX3CR1:BV605
F4/80:PE-CF594
CLEC12A:PE
GFP
G
1w PLX
2w PLX
% GFP⁺ engraftment
% GFP⁻ engraftment
% total engraftment
Control
Tam C
Tam CI
PLX + Tam CI
H
Flow cytometry
Microscopy
% GFP⁺ engraftment
1 week PLX
2 week PLX
I
Cell volume (μm³)
no. of branches
Average branch length (μm)
Maximum branch length (μm)
Embryonal mglia
Mo-mglia
J
PLX + Tam CI
Kr 50 μm
Kr 100 μm
Kr 200 μm
Kr 400 μm
Kr(Obsv-Theo)
Total cells
negative cells
GFP/tdTom
tdTom
GFP

Figure S2

Figure S2: Temporary microglia absence allows monocytes to efficiently engraft the brain parenchyma, related to Figure 2 and Figure 3.

A) Same experimental setup as in Figure 2A. *Cx3cr1*^{CreER}:*Csf1r*$^{fl/fl}$ neonates were either untreated or were injected with Tam. Depletion and repopulation was followed up at PD3, PD7, and PD16 by analyzing macrophage density per mm^2.PD3: n= 3 untreated, n = 2 Tam. PD7: n= 3 untreated, n = 3 Tam. PD16: n= 4 untreated, n = 3 Tam. PD3: 2, PD7: 2, and PD16: 3 independent experiments. **B)** Representative images of *Cx3cr1*^{CreER}:*Csf1r*$^{fl/fl}$ brains retrieved at PD16. Same experimental setup as Figure 2A). Mice were either untreated (endogenous Mglia) or received a GFP$^+$ Em-Mglia or GFP$^+$ Mo transfer. Slides stained with anti-IBA1 (red) and anti-P2RY12 (white). Scale bar, 50 μm. n = 2 untreated 3 mo, n = 3 Em-Mglia transfer 3 mo, and n = 3 Mo transfer 3 mo, from 2 independent experiment. **C)** *Cx3cr1*^{CreER}:*Csf1r*$^{fl/fl}$:5xFAD mice were treated with Tam at PD1-2 and transplanted with GFP$^+$ monocytes or Em-Mglia at PD3. Representative images of persistent engraftment of Em-Mglia (left panels) and Mo-Mglia (right panels) at 25 weeks post-transplantation. Top images: GFP (green), B-amyloid (white), and DAPI (blue). Bottom images: representative images of donor cells present in the cortex and hippocampus. GFP (green), B-amyloid (magenta), and DAPI (blue). n = 4 Em-Mglia transfer and n = 4 Mo transfer, from 2 independent experiments. Scale bar, 50 μm. **D)** Percentage of engraftment at 25 weeks post-transplantation of either GFP$^+$ Em-Mglia or Mo-Mglia in C. Significance was determined using an unpaired two-tailed *t*-test. Mean ± SD **E)** Coronal brain cryosections of 4-week-old *Cx3cr1*^{CreER}:*Csf1r*$^{fl/fl}$ that received a Mo-transfer as described in Figure 2A. Slides were stained with anti-IBA1 (red), anti-GFP (green), and anti-CLEC12A (white). Scale bar, 50 μm. Images representative of n = 2 mice, from 1 experiment. **F)** Flow cytometric gating strategy to identify Mo-Mglia engraftment in treated *Cx3cr1*^{CreER}:*Csf1r*$^{fl/fl}$ mice as shown in Figure 3A. *Cx3cr1*^{CreER}:*Csf1r*$^{fl/fl}$ mice were treated with various combinations of tamoxifen chow (C), Tamoxifen injection (I) and PLX3397 chow. All mice received an adoptive transfer of *Cx3cr1*$^{GFP/+}$ monocytes. Mice were sacrificed 4 weeks after adoptive transfers. Cells were pre-gated as CD45$^+$ live single cells. Cells were then gated as CD11b$^+$Ly6C$^-$CX3CR1$^+$F4/80$^+$. Em-Mglia were gated as GFP$^-$CLEC12A$^-$, adoptively transferred Mo-Mglia were gated as GFP$^+$CLEC12A$^+$, and endogenous Mo-Mglia were gated as GFP$^-$CLEC12A$^+$. Data representative of n = 7 (control), n = 7 (Tam C), n = 6 (Tam + CI), n = 5+7 (1 and 2w PLX + Tam CI, respectively), 10- to 11- weeks old male and female mice. Control and TamC: 2, TamCI: 3, and 1/2w PLX + TamCI: 4 independent experiments. **G)** Percentage of Mo-Mglia engraftment within experiment described in F. Left bar chart depicts adoptively transferred Mo-Mglia engraftment (GFP$^+$ CLEC12A$^+$), center bar chart depicts the endogenous Mo-Mglia engraftment (GFP$^-$ CLEC12A$^+$), and the right bar chart depicts the combined engraftment. Mean ± SD. Significances were determined using Tukey's multiple comparisons test. **H)** Comparison of the percentage of GFP$^+$ engraftment between 1W and 2W PLX + Tam CI groups described in Figure 3C and Figure S2G. Mean ± SD. Significances were determined using unpaired two-tailed *t*-tests. **I)** Morphology analysis of embryonic Mglia versus Mo-mglia. n = 5 10-11-week-old C57BL/6 male and female mice (embryonal Mglia) and n = 5 10-11-week-old *Cx3cr1*^{CreER}:*Csf1r*$^{fl/fl}$ male and female mice (Mo-Mglia) that received PLX + Tam CI treatment as described in Figure 3A. Data from 2 independent experiments. Mean ± SD. Significances were determined using unpaired two-tailed *t*-tests. **J)** Quantification of the normalized K(r) (observed value – theoretical value) for all cell detections across 50, 100, 200, and 400 μm distances for the PLX + Tam CI experiment (Figure 3F). Mean ± SD. Significances were determined using Dunnett's multiple comparisons test.

ns, *p < 0.05, **p < 0.01, ***p < 0.001, ****p < 0.0001

ctx = cortex, hipp = hippocampus, cc= corpus callosum, stria = striatum, thal = thalamus, brainst = brainstem.

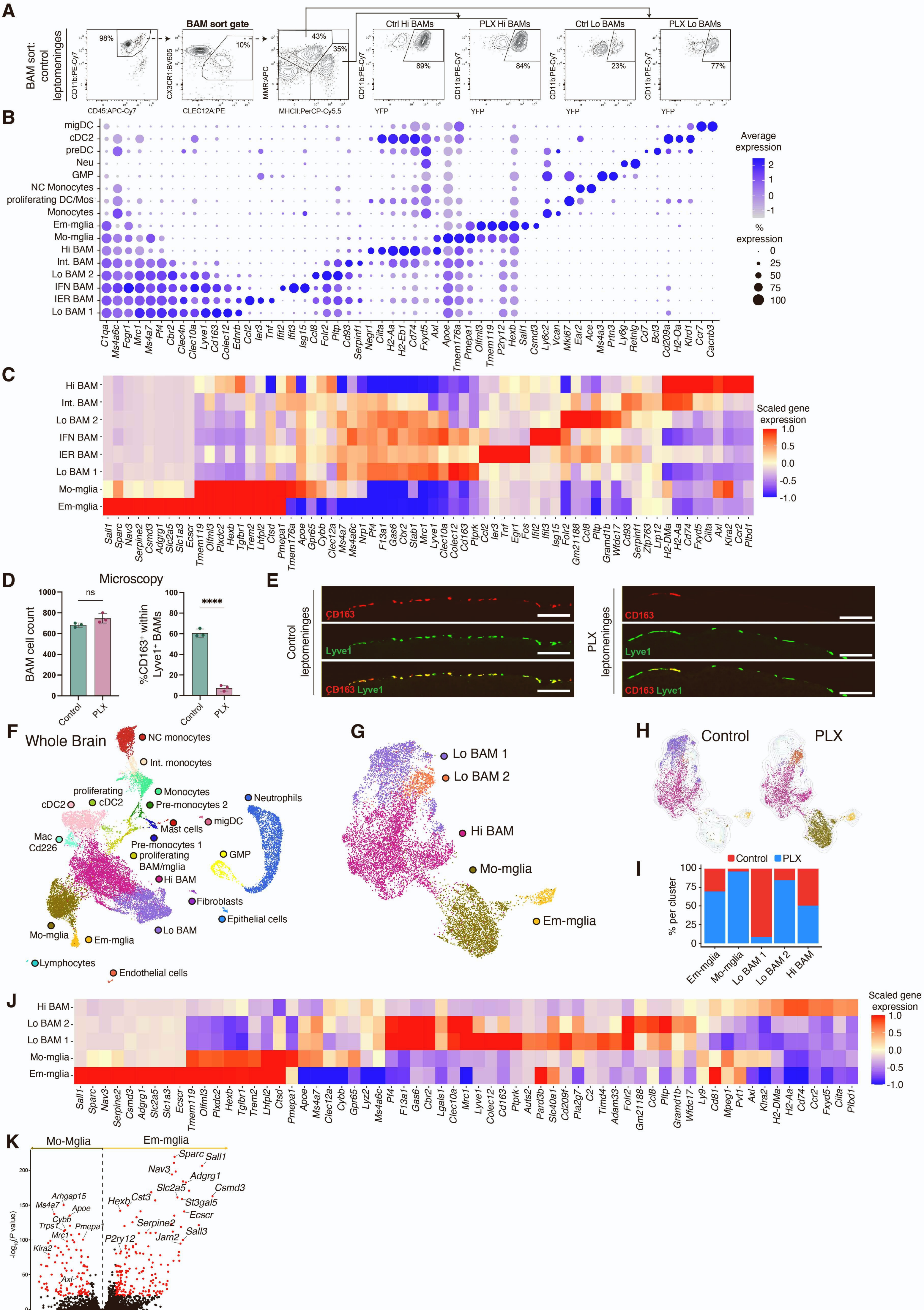
A
BAM sort: control leptomeninges
BAM sort gate
Ctrl Hi BAMs
PLX Hi BAMs
Ctrl Lo BAMs
PLX Lo BAMs
CD45:APC-Cy7
CLEC12A:PE
MHCII:PerCP-Cy5.5
YFP
B
Average expression
% expression
C
Scaled gene expression
D
Microscopy
BAM cell count
%CD163+ within Lyve1+ BAMs
Control
PLX
E
Control leptomeninges
PLX leptomeninges
CD163
Lyve1
F
Whole Brain
G
H
Control
PLX
I
% per cluster
J
Scaled gene expression
K
Mo-Mglia
Em-mglia
Log2(FC)

Figure S3

Figure S3: Transcriptional signature of leptomeningeal and whole brain Lo BAMs and Microglia in untreated and PLX3397-treated *Flt3*^{Cre}:*Yfp* mice, related to Figure 4.

A) Representative FACS gating strategy of sorting performed for the scRNA-seq in Figure 4A. Mice received control chow (Control) or 2 weeks PLX3397 chow followed by 7 weeks of repopulation on control chow (PLX & Repop). CLEC12A$^+$ cells were sorted from brain and enriched leptomeninges of n = 8 (Control) and n = 7 (PLX & Repop) *Flt3*^{Cre}*:Yfp* mice, from 1 experiment, using FACS sorting. Cells were pre-gated as CD45$^+$ live single cells. Sorted cells were gated as CD45$^+$CD11b$^+$CX3CR1$^+$CLEC12A$^+$ live single cells. Plots also show %YFP expression in Lo BAMs and Hi BAMs in control and depleted-repopulated brains. **B)** Dotplot depicting gene expression of clusters annotated in Figure 4B. **C)** Heatmap of the normalized and scaled expression of marker genes for the different macrophage subsets found in Figure 4B. **D)** Leptomeningeal BAM analysis of Lyve1 and CD163 expression in control and PLX groups. Coronal section regions analyzed correlate to leptomeninges under the skull cap. Mice were fed normal chow or PLX3397 chow for 1 week. Subsequently, they were given normal chow for 4 weeks after which they were sacrificed. n = 3 (control) and n = 3 (PLX), from 1 experiment. **E)** Representative images of mice in D. Slides stained with anti-CD163 (red), anti-Lyve1 (green), and anti F4/80 (not shown). Scale bar, 100 μm. **F)** UMAP plot of immune cells from both control and 'PLX & Repop' conditions of the whole brain. Experimental paradigm described in Figure 4A and in A. **G)** UMAP plot of reclustered macrophages retrieved from the whole brain dataset shown in F. **H)** UMAP plots depicting cells originating from the 'control' and 'PLX & Repop' conditions in B. **I)** Percentage of cells derived from 'control' or 'PLX & Repop' conditions per cluster in G. **J)** Heatmap of the normalized and scaled expression of marker genes for the different macrophage subsets found in G. **K)** Volcano plot comparing DE genes between Em-Mglia and Mo-Mglia found in G. (Log_2(FC)>0.1, -Log_{10}(adjusted *P* value)>20). *P* values were adjusted with the Bonferroni correction.

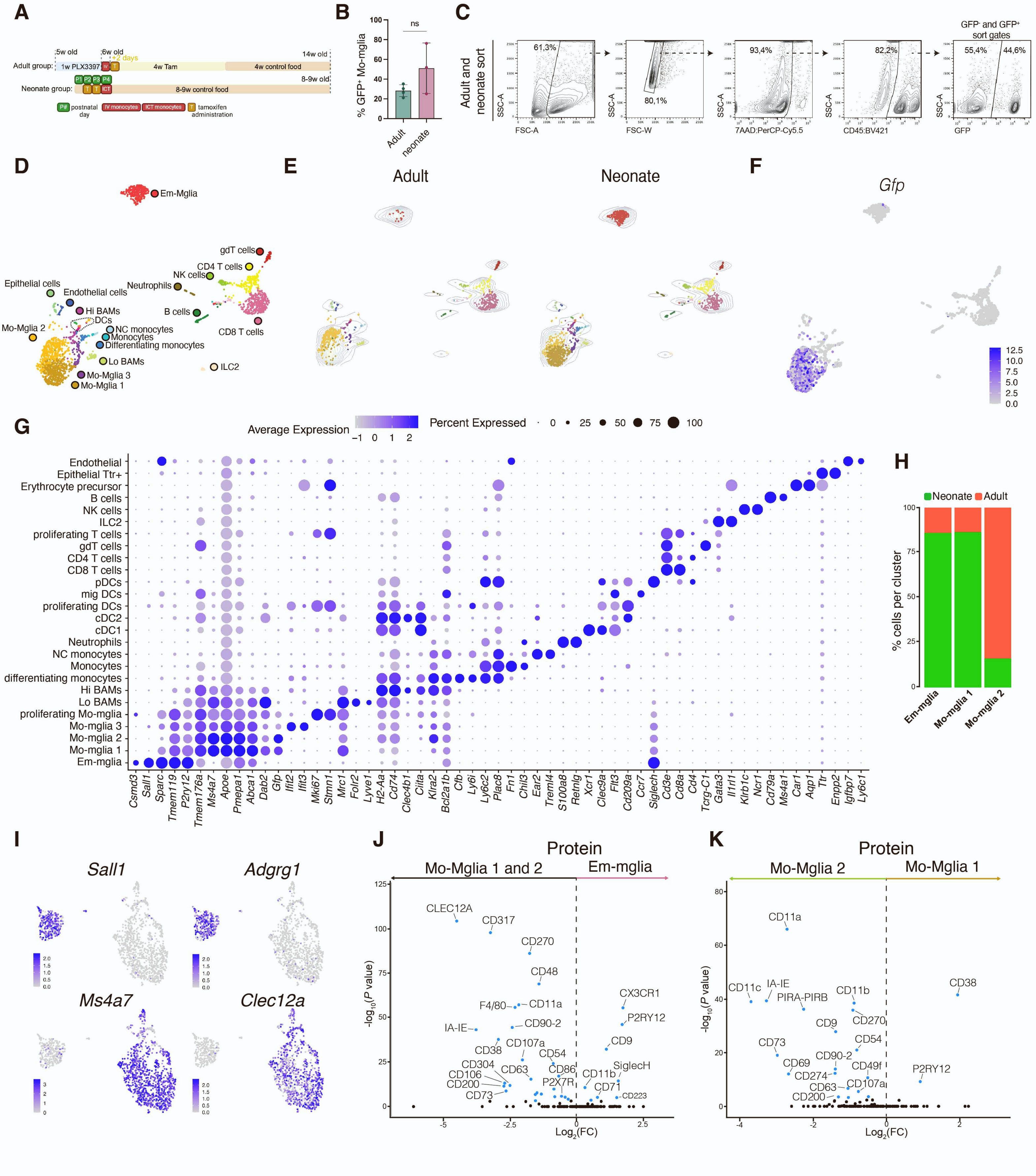
A
5w old
6w old
+2 days
14w old
Adult group:
1w PLX3397
IV
T
4w Tam
4w control food
8-9w old
Neonate group:
P1 P2 P3 P4
T T ICT
8-9w control food
P# postnatal day
IV monocytes
ICT monocytes
T tamoxifen administration
B
% GFP+ Mo-mglia
ns
Adult
neonate
C
Adult and neonate sort
61,3%
80,1%
93,4%
82,2%
GFP- and GFP+ sort gates
55,4%
44,6%
SSC-A
FSC-A
FSC-W
7AAD:PerCP-Cy5.5
CD45:BV421
GFP
D
Em-Mglia
gdT cells
CD4 T cells
NK cells
Epithelial cells
Neutrophils
Endothelial cells
Hi BAMs
B cells
DCs
Mo-Mglia 2
NC monocytes
Monocytes
CD8 T cells
Differentiating monocytes
Lo BAMs
ILC2
Mo-Mglia 3
Mo-Mglia 1
E
Adult
Neonate
F
Gfp
G
Average Expression
Percent Expressed
Endothelial
Epithelial Ttr+
Erythrocyte precursor
B cells
NK cells
ILC2
proliferating T cells
gdT cells
CD4 T cells
CD8 T cells
pDCs
mig DCs
proliferating DCs
cDC2
cDC1
Neutrophils
NC monocytes
Monocytes
differentiating monocytes
Hi BAMs
Lo BAMs
proliferating Mo-mglia
Mo-mglia 3
Mo-mglia 2
Mo-mglia 1
Em-mglia
Csmd3 Sall1 Sparc Tmem119 P2ry12 Tmem176a Ms4a7 Apoe Pmepa1 Abca1 Dab2 Gfp Ifit2 Ifit3 Mki67 Stmn1 Mrc1 Folr2 Lyve1 H2-Aa Cd74 Clec4b1 Ciita Klra2 Bcl2a1b Cfb Ly6i Ly6c2 Plac8 Fn1 Chil3 Ear2 Treml4 S100a8 Retnlg Xcr1 Clec9a Flt3 Cd209a Ccr7 Siglech Cd3e Cd8a Cd4 Tcrg-C1 Gata3 Il1rl1 Klrb1c Ncr1 Cd79a Ms4a1 Car1 Aqp1 Ttr Enpp2 Igfbp7 Ly6c1
H
Neonate
Adult
% cells per cluster
Em-mglia
Mo-Mglia 1
Mo-Mglia 2
I
Sall1
Adgrg1
Ms4a7
Clec12a
J
Protein
Mo-Mglia 1 and 2
Em-mglia
-log10(P value)
Log2(FC)
CLEC12A
CD317
CD270
CD48
F4/80
CD11a
CD90-2
IA-IE
CD107a
CD38
CD54
CD304
CD106
CD200
CD73
CD63
CD86
P2X7R
CX3CR1
P2RY12
CD9
SiglecH
CD11b
CD71
CD223
K
Protein
Mo-Mglia 2
Mo-Mglia 1
CD11a
CD11c
IA-IE
PIRA-PIRB
CD11b
CD38
CD270
CD9
CD54
CD73
CD90-2
CD69
CD274
CD49f
P2RY12
CD63
CD107a
CD200

Figure S4a

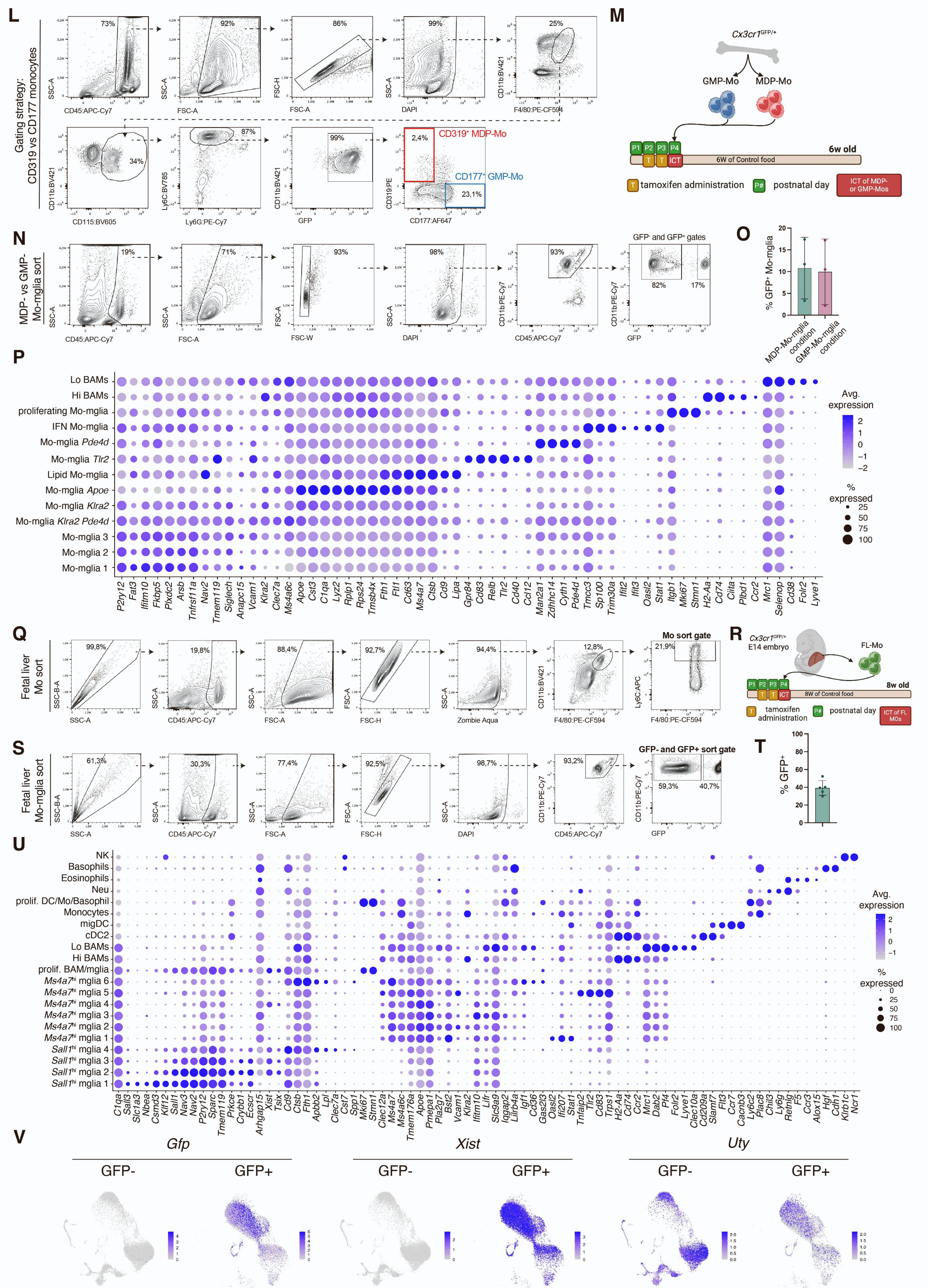
L
Gating strategy: CD319 vs CD177 monocytes
73%
92%
86%
99%
25%
CD45:APC-Cy7
FSC-A
DAPI
F4/80:PE-CF594
CD11b:BV421
34%
87%
99%
2,4%
CD319+ MDP-Mo
CD177+ GMP-Mo
23,1%
CD115:BV605
Ly6G:PE-Cy7
GFP
CD177:AF647
CD319:PE
Ly6C:BV785
M
Cx3cr1GFP/+
GMP-Mo
MDP-Mo
P1 P2 P3 P4
T T ICT
6W of Control food
6w old
tamoxifen administration
postnatal day
ICT of MDP- or GMP-Mos
N
MDP- vs GMP- Mo-mglia sort
19%
71%
93%
98%
93%
GFP- and GFP+ gates
82%
17%
CD45:APC-Cy7
FSC-A
FSC-W
DAPI
CD11b:PE-Cy7
GFP
O
% GFP+ Mo-mglia
MDP-Mo-mglia condition
GMP-Mo-mglia condition
P
Lo BAMs
Hi BAMs
proliferating Mo-mglia
IFN Mo-mglia
Mo-mglia Pde4d
Mo-mglia Tlr2
Lipid Mo-mglia
Mo-mglia Apoe
Mo-mglia Klra2
Mo-mglia Klra2 Pde4d
Mo-mglia 3
Mo-mglia 2
Mo-mglia 1
Avg. expression
% expressed
P2ry12 Fat3 Ifitm10 Fkbp5 Plxdc2 Arsb Tnfrsf11a Nav2 Tmem119 Siglech Anapc15 Vcam1 Klra2 Clec7a Ms4a6c Apoe Cst3 C1qa Lyz2 Rplp1 Rps24 Tmsb4x Fth1 Ftl1 Cd63 Ms4a7 Ctsb Cd9 Lipa Gpr84 Cd83 Relb Tlr2 Cd40 Ccl12 Man2a1 Zdhhc14 Cyth1 Pde4d Tmcc3 Sp100 Trim30a Ifit2 Ifit3 Oasl2 Stat1 Itgb1 Mki67 Stmn1 H2-Aa Cd74 Ciita Plbd1 Ccr2 Mrc1 Selenop Cd38 Folr2 Lyve1
Q
Fetal liver Mo sort
99,8%
19,8%
88,4%
92,7%
94,4%
12,8%
Mo sort gate
21,9%
SSC-A
CD45:APC-Cy7
FSC-A
FSC-H
Zombie Aqua
F4/80:PE-CF594
Ly6C:APC
R
Cx3cr1GFP/+
E14 embryo
FL-Mo
8w old
8W of Control food
tamoxifen administration
postnatal day
ICT of FL MOs
S
Fetal liver Mo-mglia sort
61,3%
30,3%
77,4%
92,5%
98,7%
93,2%
GFP- and GFP+ sort gate
59,3%
40,7%
DAPI
CD45:APC-Cy7
GFP
T
% GFP+
U
NK
Basophiles
Eosinophils
Neu
prolif. DC/Mo/Basophil
Monocytes
migDC
cDC2
Lo BAMs
Hi BAMs
prolif. BAM/mglia
Ms4a7hi mglia 6
Ms4a7hi mglia 5
Ms4a7hi mglia 4
Ms4a7hi mglia 3
Ms4a7hi mglia 2
Ms4a7hi mglia 1
Sall1hi mglia 4
Sall1hi mglia 3
Sall1hi mglia 2
Sall1hi mglia 1
Avg. expression
% expressed
C1qa Sall3 Slc1a3 Nbea Csmd3 Klf12 Sall1 Nav3 Nav2 P2ry12 Sparc Tmem119 Prkce Crybb1 Ecscr Arhgap15 Xist Tsix Cd9 Ctsb Fth1 Apbb2 Lpl Clec7a Cst7 Spp1 Mki67 Stmn1 Clec12a Ms4a7 Ms4a6c Tmem176a Apoe Pmepa1 Pla2g7 Bst2 Vcam1 Klra2 Ifitm10 Lifr Slc9a9 Iqgap2 Lilrb4a Igf1 Cd36 Gas2l3 Oasl2 Ifi207 Stat1 Tnfaip2 Tlr2 Cd83 Trps1 H2-Aa Cd74 Ccr2 Mrc1 Dab2 Pf4 Folr2 Lyve1 Clec10a Cd209a Slamf7 Flt3 Ccr7 Cacnb3 Ly6c2 Plac8 Chil3 Ly6g Retnlg F5 Ccr3 Alox15 Hgf Cdh1 Klrb1c Ncr1
V
Gfp
Xist
Uty
GFP-
GFP+

Figure S4b

Figure S4: Bone marrow monocytes give rise to microglia which remain transcriptionally distinct from embryonal microglia while fetal liver monocytes generate bona fide microglia, related to Figure 4 and Figure 5.

A) Experimental layout for the CITE-seq in Figure 4I. Adult mice received the 1w PLX + Tam CI treatment and an adoptive transfer of *Cx3cr1*$^{GFP/+}$ monocytes. After 4 weeks on Tam chow, mice were switched to control chow for an additional 4 weeks before they were sacrificed. Neonates received intraperitoneal tamoxifen over 2 days at PD2 and PD3, and at PD 4 they were intracerebrally injected with *Cx3cr1*$^{GFP/+}$ monocytes. 8 weeks later, this group was sacrificed. Both conditions were processed for scRNA-seq on the same day. Brains were harvested from n = 4 (adult) 14-week-old males and n = 3 (neonate) 8-week-old females, from 1 experiment. CD45^{+}GFP^{+} and CD45^{+}GFP^{-} cells were FACS sorted from whole brains of both groups. GFP^{+} and GFP^{-} cells were combined in a 1:1 ratio after sorting. **B)** Percentage of CD45^{+}GFP^{+} cells in the brain per condition. Mean ± SD n = 4 (adult) n = 3 (neonate)**.** Significance was determined using an unpaired two-tailed *t*-tests. **C)** Representative FACS gating strategy for the sorted cells used for CITE-seq. **D)** UMAP plot of immune cells from both the neonate and adult conditions. **E)** UMAP plots depicting cells originating from either the adult or neonate conditions. **F)** UMAP plot showing the expression of *Gfp* in the dataset from D. **G)** Dotplot depicting gene expression of clusters annotated in D. **H)** Percentage of cells derived from adult or neonate conditions for the Em-Mglia, Mo-Mglia 1, and Mo-Mglia 2 clusters from Figure 4J. **I)** UMAP plots showing expression of the indicated genes in the dataset from Figure 4J. **J)** Volcano plot comparing DE proteins between Em-mglia and the combined ‘Mo-Mglia 1 and Mo-Mglia 2’ clusters found in Figure 4J. (Log_2(FC)>0.25, -Log_{10}(adjusted *P* value)>2). *P* values were adjusted with the Bonferroni correction. **K)** Volcano plot comparing DE proteins between Mo-Mglia 1 and Mo-Mglia 2 clusters found in Figure 4J. (Log_2(FC)>0.25, -Log_{10}(adjusted *P* value)>2). *P* values were adjusted with the Bonferroni correction. **L)** FACS gating strategy for sorting of GMP- and MDP-monocytes. **M)** Schematic of experiment layout. CD319^{+} (MDP-Mo) and CD177^{+} (GMP-Mo) bone marrow monocytes were FACS sorted from *Cx3cr1*$^{GFP/+}$ mice and intracerebrally injected in PD4 *Cx3cr1*^{CreER}:*Csf1r*$^{fl/fl}$ neonates that received tam-treatment at PD2 and PD3 (experimental paradigm similar to Figure 4I). Six weeks later, CD45^{+} CD11b^{+} GFP^{+} cells were FACS sorted from whole brains. Sorting strategy shown in N. Brains were harvested from n = 3 (MDP-Mo) and n = 3 (GMP-Mo) 6-week-old males and females, from 1 experiment. **N)** FACS gating strategy of GMP-Mo-Mglia and MDP-Mo-Mglia cells sorted for scRNA-seq analysis in M. **O)** Percentage of GFP^{+} engraftment within CD45^{+}CD11b^{+} cells in the mice described in M. n = 3 MDP-Mo-Mglia and n = 3 GMP-Mo-Mglia, Mean ± SD. **P)** Dotplot depicting gene expression for the clusters shown in Figure 5D. **Q)** FACS gating strategy for sorting of fetal liver monocytes used in Figure 5H. **R)** *Cx3cr1*$^{GFP/+}$ E14 fetal liver monocytes were FACS sorted and intracerebrally injected in PD4 *Cx3cr1*^{CreER}:*Csf1r*$^{fl/fl}$ neonates that received tam-treatment at PD2 and PD3 (experiment similar to Figure 4I). 8 weeks later CD45^{+}CD11b^{+}GFP^{+} and GFP^{-} cells were FACS sorted from whole brains. GFP^{+} and GFP^{-} cells were individually processed after FACS sorting and later combined *in-silico*. Brain myeloid cell sorting strategy available in S. Brains harvested from n = 5 males. **S)** FACS gating strategy of Fetal liver Mo-Mglia cells sorted for scRNA-seq analysis in R. **T)** Percentage of CD45^{+}CD11b^{+}GFP^{+} engraftment in the mice described in R,S and Figure 5H. n = 5, Mean ± SD. **U)** Dotplot depicting gene expression for the clusters annotated in Figure 5I. **V)** UMAP plots showing expression of selected genes split per condition in the dataset shown in Figure 5I.

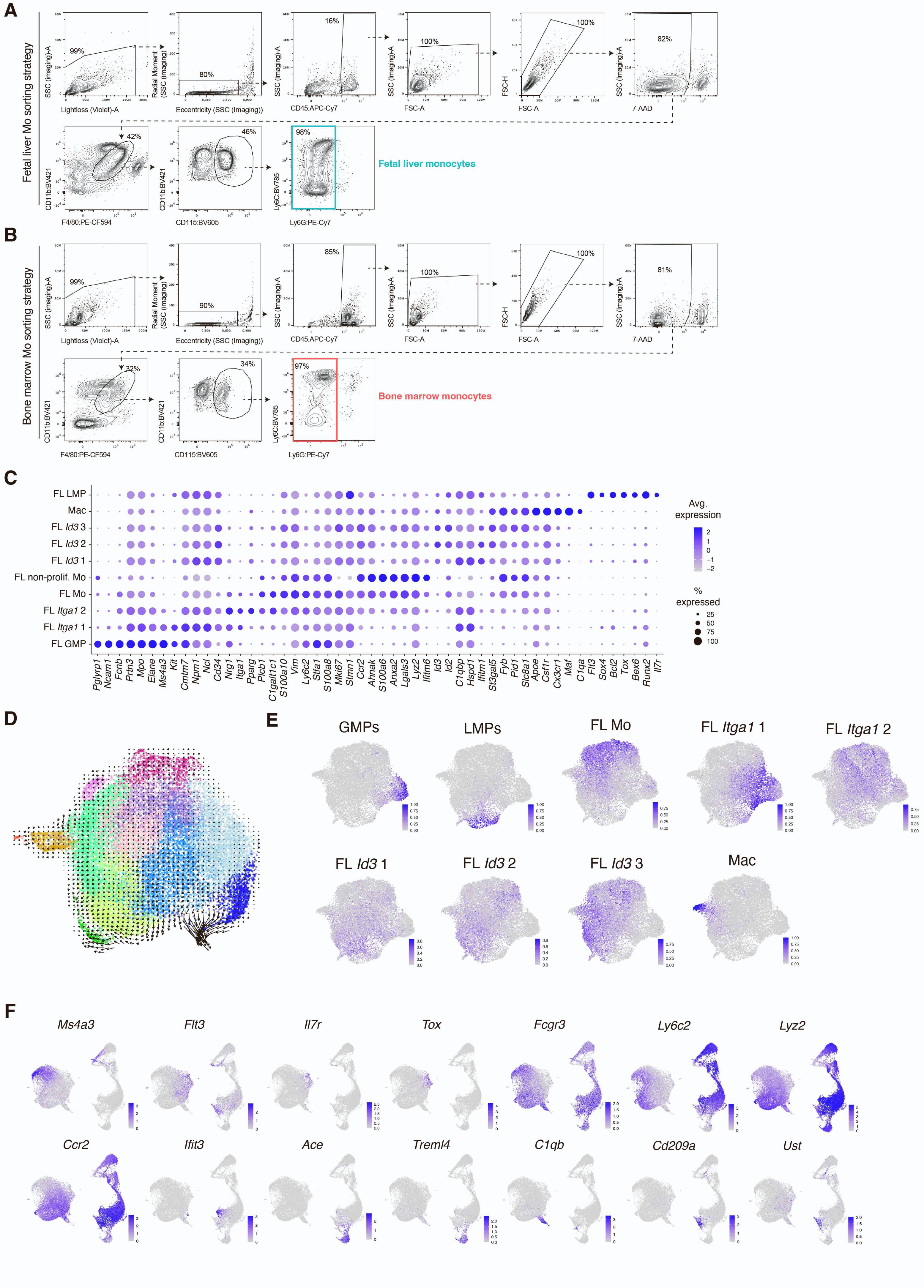
A
Fetal liver Mo sorting strategy
99%
SSC (imaging)-A
Lightloss (Violet)-A
80%
Radial Moment (SSC (imaging))
Eccentricity (SSC (Imaging))
16%
SSC (Imaging)-A
CD45:APC-Cy7
100%
FSC-A
FSC-H
82%
7-AAD
42%
CD11b:BV421
F4/80:PE-CF594
46%
CD115:BV605
98%
Ly6C:BV785
Ly6G:PE-Cy7
Fetal liver monocytes
B
Bone marrow Mo sorting strategy
99%
90%
85%
100%
81%
32%
34%
97%
Bone marrow monocytes
C
FL LMP
Mac
FL Id3 3
FL Id3 2
FL Id3 1
FL non-prolif. Mo
FL Mo
FL Itga1 2
FL Itga1 1
FL GMP
Pglyrp1 Ncam1 Fcnb Prtn3 Mpo Elane Ms4a3 Kit Cmtm7 Npm1 Ncl Cd34 Nrg1 Itga1 Pparg Plcb1 C1galt1c1 S100a10 Vim Ly6c2 Stfa1 S100a8 Mki67 Stmn1 Ccr2 Ahnak S100a6 Anxa2 Lgals3 Lyz2 Ifitm6 Id3 Id2 C1qbp Hspd1 Ifitm1 St3gal5 Fyb Pld1 Slc8a1 Apoe Csf1r Cx3cr1 Maf C1qa Flt3 Sox4 Bcl2 Tox Bex6 Runx2 Il7r
Avg. expression
% expressed
25
50
75
100
D
E
GMPs
LMPs
FL Mo
FL Itga1 1
FL Itga1 2
FL Id3 1
FL Id3 2
FL Id3 3
Mac
F
Ms4a3
Flt3
Il7r
Tox
Fcgr3
Ly6c2
Lyz2
Ccr2
Ifit3
Ace
Treml4
C1qb
Cd209a
Ust

Figure S5

Figure S5. Fetal liver monocyte and bone marrow monocyte FACS sorting strategies, related to Figure 6.

A) FACS gating strategy for sorting of bone marrow monocytes. **B)** FACS gating strategy for sorting of fetal liver monocytes. **C)** Dotplot depicting gene expression for the FL clusters shown in Figure 6B. **D)** RNA velocity plot of the FL scRNAseq dataset from Figure 6B. **E)** UMAP plots showing prediction scores for each FL Mo scRNA-seq cluster from Figure 6B calculated for each cells of the snATAC-seq dataset from Figure 6D. **F)** UMAP plots showing expression of selected genes in the dataset from Figure 6F.

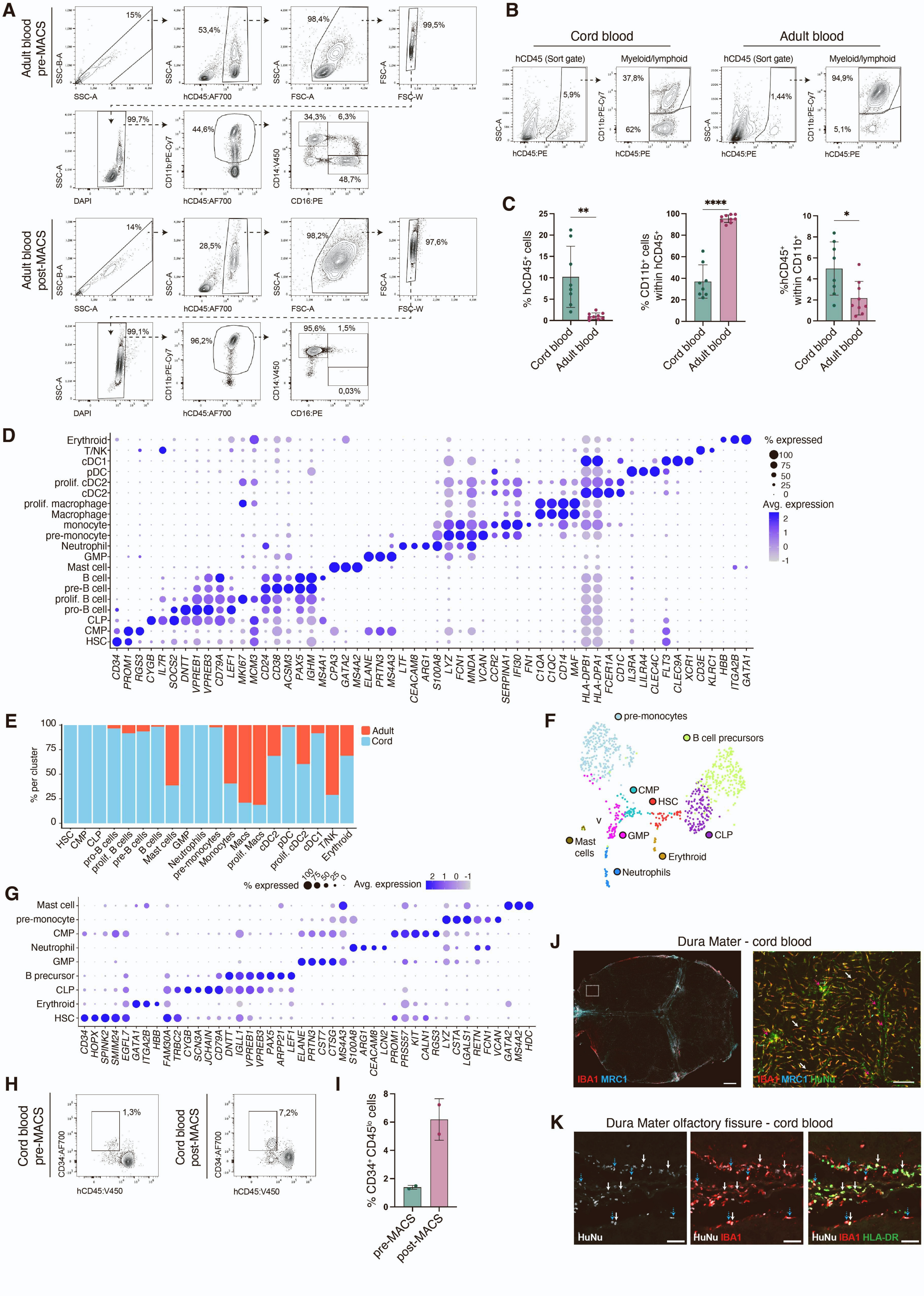
A
Adult blood pre-MACS
15%
53,4%
98,4%
99,5%
SSC-B-A
SSC-A
hCD45:AF700
FSC-A
FSC-W
99,7%
44,6%
34,3%
6,3%
48,7%
DAPI
CD11b:PE-Cy7
CD14:V450
CD16:PE
Adult blood post-MACS
14%
28,5%
98,2%
97,6%
99,1%
96,2%
95,6%
1,5%
0,03%
B
Cord blood
Adult blood
hCD45 (Sort gate)
Myeloid/lymphoid
5,9%
37,8%
62%
1,44%
94,9%
5,1%
hCD45:PE
C
**
****
*
% hCD45+ cells
% CD11b+ cells within hCD45+
%hCD45+ within CD11b+
Cord blood
Adult blood
D
Erythroid
T/NK
cDC1
pDC
prolif. cDC2
cDC2
prolif. macrophage
Macrophage
monocyte
pre-monocyte
Neutrophil
GMP
Mast cell
B cell
pre-B cell
prolif. B cell
pro-B cell
CLP
CMP
HSC
CD34 PROM1 RGS3 CYGB IL7R SOCS2 DNTT VPREB1 VPREB3 CD79A LEF1 MKI67 MCM3 CD24 CD38 ACSM3 PAX5 IGHM MS4A1 CPA3 GATA2 MS4A2 ELANE PRTN3 MS4A3 LTF CEACAM8 ARG1 S100A8 LYZ FCN1 MNDA VCAN CCR2 SERPINA1 IFI30 FN1 C1QA C1QC CD14 MAF HLA-DPB1 HLA-DPA1 FCER1A CD1C IL3RA LILRA4 CLEC4C FLT3 CLEC9A XCR1 CD3E KLRC1 HBB ITGA2B GATA1
% expressed
100
75
50
25
0
Avg. expression
E
% per cluster
Adult
Cord
HSC CMP CLP pro-B cells prolif. B cells pre-B cells B cells Mast cells GMP Neutrophils pre-monocytes Monocytes Macs prolif. Macs cDC2 pDC prolif. cDC2 cDC1 T/NK Erythroid
F
pre-monocytes
B cell precursors
CMP
HSC
GMP
CLP
Mast cells
Erythroid
Neutrophils
G
Mast cell
pre-monocyte
CMP
Neutrophil
GMP
B precursor
CLP
Erythroid
HSC
CD34 HOPX SPINK2 SMIM24 EGFL7 GATA1 ITGA2B HBB FAM30A TRBC2 CYGB SCN3A JCHAIN CD79A DNTT IGLL1 VPREB1 VPREB3 PAX5 ARPP21 LEF1 ELANE PRTN3 CST7 CTSG MS4A3 S100A8 ARG1 CEACAM8 LCN2 PROM1 PRSS57 KIT CALN1 RGS3 LYZ CSTA LGALS1 RETN FCN1 VCAN GATA2 MS4A2 HDC
H
Cord blood pre-MACS
Cord blood post-MACS
1,3%
7,2%
CD34:AF700
hCD45:V450
I
% CD34+CD45lo cells
pre-MACS
post-MACS
J
Dura Mater - cord blood
IBA1 MRC1
IBA1 MRC1 HuNu
K
Dura Mater olfactory fissure - cord blood
HuNu
HuNu IBA1
HuNu IBA1 HLA-DR

Figure S6a

L

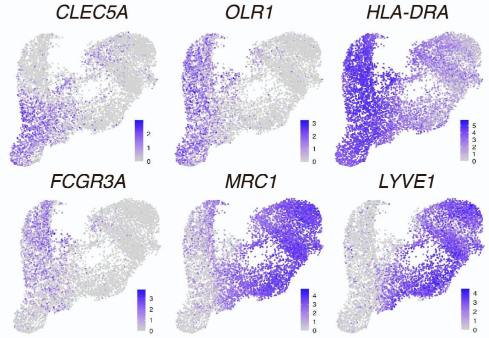

M

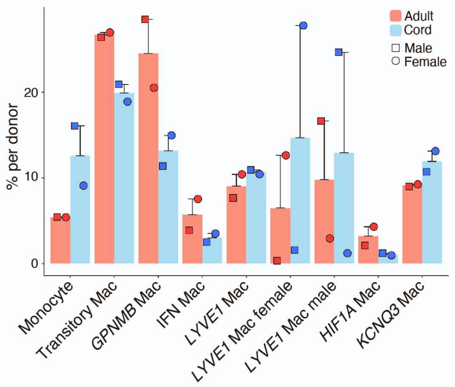

N

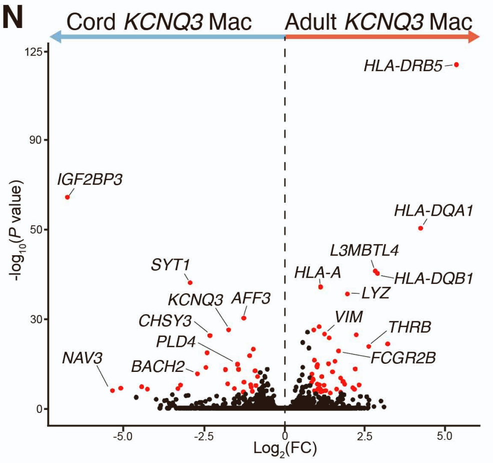

O

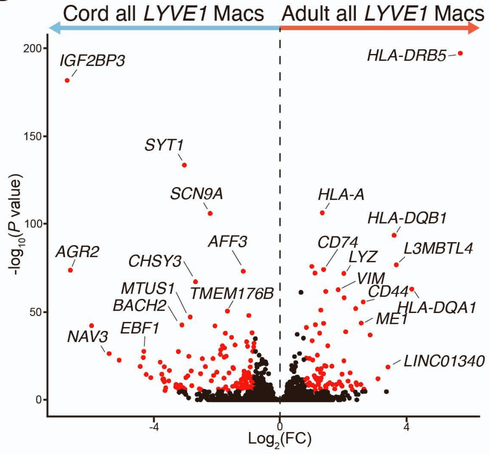

P

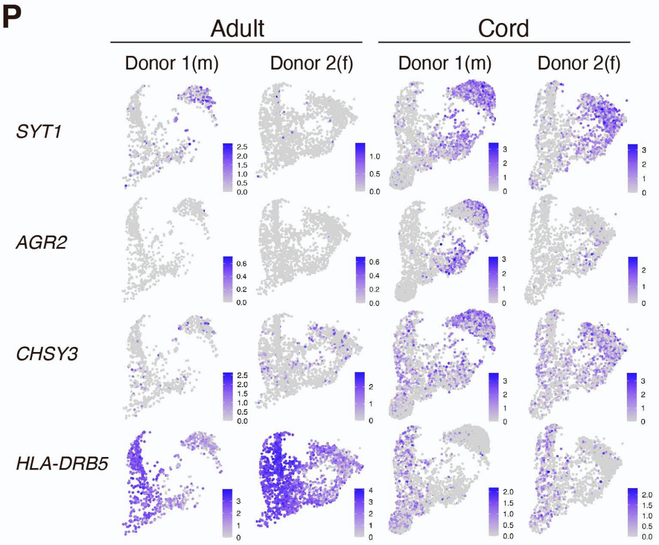

Q

Cord *KCNQ3* Mac:

GO:0050804: modulation of chemical synaptic transmission

SYT1, PDE9A, KCNQ3, CYFIP1

GO:0006897: endocytosis

SYT1, KCNQ3, PLD4, ARHGAP12

GO:0071277: cellular response to calcium ion

MELTF, SYT1, SCN9A, PLEKHA1

Adult *KCNQ3* Mac:

GO:0048002: antigen processing and presentation

HLA-DRB5, CD300E, FCGRA3, CFD

R-HSA-2172127: DAP12 interactions

CD300E, LYZ, CLEC5A, MGAM

GO:0006954: inflammatory response

LYZ, CD44, IER3, TIMP1

R

Cord all *LYVE1* Macs:

GO:0002764: immune response-regulating signaling pathway

IGHM, CD79B, IGLL1, IGLC1, CD24, BACH2

GO:1903034: regulation of response to wounding

AGR2, SPP1, IGLL1, CD24, VPREB1, GNG11

GO:0098900: regulation of action potential

RAMP1, SYT1, KCNQ3, FCMR, MYO1D

Adult all *LYVE1* Macs:

GO:0019882 antigen processing and presentation

HLA-DRB5, CD44, CLEC10A, VIM, PTPN22

R-HSA-6798695 Neutrophil degranulation

RNASE2, LYZ, HK3, CLEC5A, CLEC10A, VIM, ITGAX

GO:0001819 positive regulation of cytokine production

LURAP1, PTGER4, CD151, CLEC4E, CD300LF, CD74

Figure S6b

Figure S6. Human cord and adult blood monocytes engraft as BAMs and microglia-like cells in the mouse brain, related to Figure 7.

A) Flow cytometry plots revealing $CD14^+$ classical monocyte purity before and after MACS purification from adult blood. Classical monocytes are gated as $CD45^+CD11b^+CD14^+CD16^-$. **B)** Representative sorting gates for scRNA-seq experiment described in Figure 7A. Cells were pre-gated as live single cells. **C)** Left bar chart: percentage of $hCD45^+$ engraftment within the hCSF1 mouse brains. Center bar chart: percentage of $CD11b^+$ cells within $hCD45^+$ fraction. Right bar chart: percentage of $hCD45^+$ cells within total $CD11b^+$ fraction. Charts related to scRNA-seq experiment described in Figure 7A. Significances were determined using unpaired two-tailed *t*-tests. **D)** Dotplot depicting gene expression of the annotated clusters in Figure 7B. **E)** Proportion of cells derived from cord or adult blood conditions per cluster in Figure 7B. **F)** UMAP of reclustered progenitor cells from Figure 7B. CMP (common myeloid progenitor), GMP (granulocyte/monocyte progenitor), CLP (common lymphoid progenior) **G)** Dotplot depicting gene expression of the annotated clusters in F. **H)** Flow cytometry plots revealing the percentage of $CD34^+$ cells within live single cells from cord blood before and after MACS purification. Cells were pre-gated as live, single cells. n=2, from 1 experiment. **I)** Percentage of $CD34^+hCD45^{lo}$ cells from cord blood before and after MACS purification in H. n =2, from 1 experiment. Mean ± SD. **J)** Meninges whole mount of hCSF1:$Rag2^{-/-}$:$\gamma c^{-/-}$ mice that received the cord blood intracerebral injection. Scale bar, 1 mm. Right image: inset demonstrating $IBA1^+HuNu^+MRC1^-$ cells (white arrows) and small circular $IBA1^-HuNu^+MRC1^-$ cells (dashed magenta arrows). Scale bar, 50 μm. Representative images of n = 3 mice, from 1 experiment. **K)** Coronal brain sections stained with anti-IBA1 (red), HLA-DR (green), and anti-human nucleus (white). $IBA1^+HuNu^+HLA\text{-}DR^+$ cells (white arrows) and $IBA1^+HuNu^+HLA\text{-}DR^-$ cells (dashed blue arrows). Scale bars, 50 μm. Representative images of n = 3 mice, from one experiment. **L)** UMAP plot showing the expression of selected genes in the dataset shown in Figure 7F. **M)** Percentage of cells derived from cord blood or adult blood conditions for the cluster annotated in Figure 7F. Datapoints are further split up into male and female donors per condition per cluster. n = 2, Mean ± SD **N)** Volcano plot comparing DE genes of cells originating from the adult versus cord blood condition, specifically for the *KCNQ3* Mac cluster found in Figure 7F. (Log_2(FC)>0.8, -Log_{10}(adjusted *P* value)>5). *P* values were adjusted with the Bonferroni correction. **O)** Volcano plot comparing DE genes of the combined *LYVE1* Mac clusters (*LYVE1* Mac, *LYVE1* Mac (female), and *LYVE1* Mac (male)) between the conditions 'cord blood' and 'adult blood' found in Figure 7F. (Log_2(FC)>0.8, -Log_{10}(adjusted *P* value)>5). *P* values were adjusted with the Bonferroni correction. **P)** UMAP plots depicting expression of the indicated genes, split per condition and per donor (m= male, f= female) for the dataset from Figure 7F. **Q)** Chosen terms retrieved from a gene ontology enrichment analysis on the DE genes from N. **R)** Chosen terms retrieved from a gene ontology enrichment analysis on the DE genes from O.

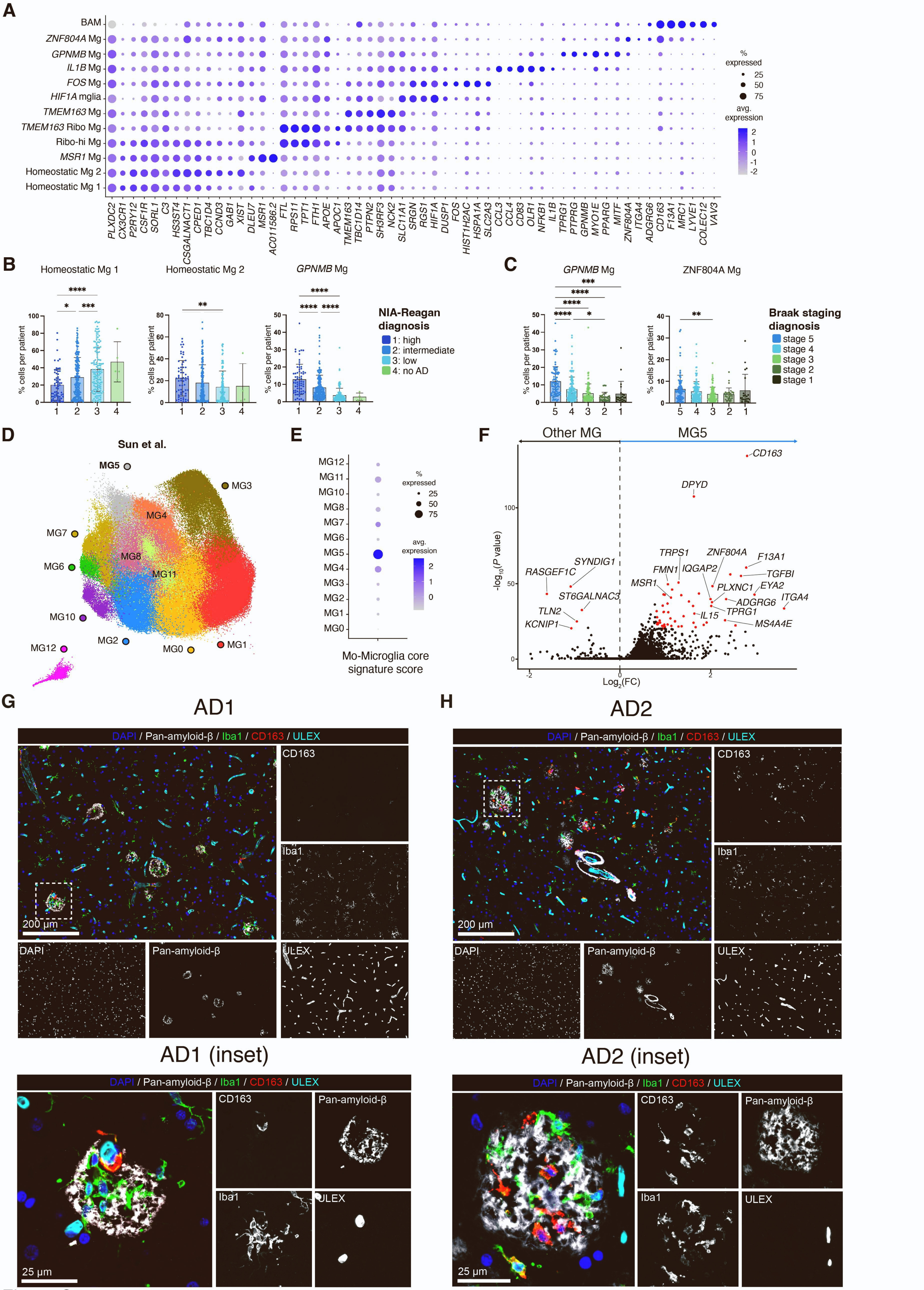
A
BAM
ZNF804A Mg
GPNMB Mg
IL1B Mg
FOS Mg
HIF1A mglia
TMEM163 Mg
TMEM163 Ribo Mg
Ribo-hi Mg
MSR1 Mg
Homeostatic Mg 2
Homeostatic Mg 1
% expressed
25
50
75
avg. expression
2
1
0
-1
-2
PLXDC2 CX3CR1 P2RY12 CSF1R SORL1 C3 HS3ST4 CSGALNACT1 CPED1 TBC1D4 CCND3 GAB1 XIST DLEU7 MSR1 AC011586.2 FTL RPS11 TPT1 FTH1 APOE APOC1 TMEM163 TBC1D14 PTPN2 SH3RF3 NCK2 SLC11A1 SRGN RGS1 HIF1A DUSP1 FOS HIST1H2AC HSPA1A SLC2A3 CCL3 CCL4 CD83 OLR1 NFKB1 IL1B TPRG1 PTPRG GPNMB MYO1E PPARG MITF ZNF804A ITGA4 ADGRG6 CD163 F13A1 MRC1 LYVE1 COLEC12 VAV3
B
Homeostatic Mg 1
Homeostatic Mg 2
GPNMB Mg
% cells per patient
NIA-Reagan diagnosis
1: high
2: intermediate
3: low
4: no AD
C
GPNMB Mg
ZNF804A Mg
Braak staging diagnosis
stage 5
stage 4
stage 3
stage 2
stage 1
D
Sun et al.
MG5 MG3 MG4 MG7 MG6 MG8 MG11 MG10 MG2 MG0 MG1 MG12
E
MG12 MG11 MG10 MG8 MG7 MG6 MG5 MG4 MG3 MG2 MG1 MG0
Mo-Microglia core signature score
F
Other MG
MG5
CD163 DPYD TRPS1 ZNF804A F13A1 SYNDIG1 RASGEF1C FMN1 IQGAP2 TGFBI MSR1 PLXNC1 EYA2 ST6GALNAC3 ADGRG6 ITGA4 TLN2 TPRG1 IL15 KCNIP1 MS4A4E
-log10(P value)
Log2(FC)
G
AD1
DAPI / Pan-amyloid-β / Iba1 / CD163 / ULEX
CD163
Iba1
200 µm
DAPI
Pan-amyloid-β
ULEX
H
AD2
AD1 (inset)
AD2 (inset)
25 µm
Figure S7

Figure S7: Microglial identity and abundance in human Alzheimer's disease brains, related to Figure 7.

A) Dotplot depicting gene expression of the annotated clusters in Figure 7H. **B)** Relative abundance per cluster shown in Figure 7H in donors that were stratified based on the NIA-Reagan diagnosis score. Donors stratified per score: n= 68 'score 1', n = 198 'score 2', n = 153 'score 3', and n = 5 'score 4'. Significances were determined using Tukey's multiple comparisons test where column 4 was omitted due to lack of enough datapoints to perform relevant statistical tests. ns, *p < 0.05, **p < 0.01, ***p < 0.001, ****p < 0.0001 **C)** Relative abundance per cluster shown in Figure 7H in donors that were stratified based on the Braak staging diagnosis score. Staging scores 6 and 0 were omitted due to lack of sufficient data points. Donors stratified per score: n= 3 'score 6', n = 91 'score 5', n = 143 'score 4', and n = 124 'score 3', n = 32 'score 2', n = 24 'score 1', and n = 7 'score 0'. Significances were determined using Tukey's multiple comparisons. ns, *p < 0.05, **p < 0.01, ***p < 0.001, ****p < 0.0001 **D)** UMAP plot of 152 459 microglia nuclei of postmortem brain samples from 217 AD and 226 control individuals from the ROSMAP cohort of Sun et al. based on the original analysis of the authors. **E)** Dotplot of a 'Mo-Microglia core signature score' given to all the cells in D. The score shows the enrichment for genes, that are upregulated in *ZNF804A* Mg compared to the remaining cells in the ROSMAP snRNAseq dataset shown in Figure 7H (p_val_adj<0.05 and log2FC>0.8) **F)** Volcano plot comparing DE genes of the MG5 cluster against all other Mg found in D. (Log_2(FC)>0.8, -Log_{10}(adjusted *P* value)>20). *P* values were adjusted with the Bonferroni correction. **G, H)** Representative images of human frontal cortex of Alzheimer's disease patients (AD1 and AD2, respectively) stained with Iba1 (green), CD163 (red), pan-amyloid (white), ULEX (cyan), DAPI (blue). Images vessel associated Iba1$^+$CD163$^+$ cell in patient AD1; and reveal many non-vessel associated Iba1$^+$CD163$^+$ cells in patient AD2.

Table S1: antibodies for flow cytometry and FACS, related to STAR methods.

Antigen	Fluorophore	Reactivity	Clone	RRID	ID	Manufacturer	Dilution
c-kit	PE	mouse	2B8	AB_313217	105808	BioLegend	1/1000
CD11b	PE-Cy7	mouse/ human	M1/70	AB_312799	101216	BioLegend	1/1000
CD11b	BV421	mouse/ human	M1/70	AB_2562904	101251	BioLegend	1/1000
CD11c	BV605	mouse	N418	AB_2562415	117334	BioLegend	1/1000
CD14	V450	human	M5E2	AB_10611582	561390	BD Biosciences	1/1000
CD14	BV421	human	M5E2	AB_2739154	565283	BD Biosciences	1/1000
CD16	PE	human	3G8	AB_314208	302008	BioLegend	1/1000
CD24	AF700	mouse	M1/69	AB_2566730	101836	BioLegend	1/1000
CD34	AF700	human	561	AB_2632722	343621	BioLegend	1/1000
CD45	PE	human	HI30	AB_314396	304008	BioLegend	1/1000
CD45	AF700	human	HI30	AB_493761	304024	Biolegend	1/1000
CD45	BV421	mouse	30-F11	AB_2562559	103134	BioLegend	1/1000
CD45	APC-Cy7	mouse	30-F11	AB_312981	103116	BioLegend	1/1000
CD45	V450	human	HI30	AB_1645573	560367	BD Biosciences	1/1000
CD115	BV605	mouse	AFS98	AB_2562760	135517	BioLegend	1/1000
CD177	AF647	Mouse	Y127		566599	BD Biosciences	1/1000
CD319	PE	Mouse	4G2	AB_2632676	152005	BioLegend	1/1000
CLEC12A	PE	mouse	5D3/CL EC12A	AB_11126747	143403	BioLegend	1/1000
CX3CR1	BV605	mouse	SA011 F11	AB_2565937	149027	BioLegend	1/500
F4/80	PE-CF594	mouse	T45-2342	AB_2734770	565613	BD Biosciences	1/500
FOLR2	APC	mouse	10/FR2	AB_2721313	153306	BioLegend	1/1000
I-A/I-E	PerCP-Cy5.5	mouse	M5/114 .15.2	AB_2191071	107626	BioLegend	1/1000
Ly6C	APC	mouse	HK1.4	AB_1732076	128016	BioLegend	1/1000
Ly6C	BV510	mouse	HK1.4	AB_2562351	128033	BioLegend	1/1000
Ly6C	BV785	Mouse	HK1.4	AB_2565852	128041	BioLegend	1/1000
Ly6G	APC	mouse	1A8	AB_2227348	127613	BioLegend	1/1000
Ly6G	PE-Cy7	Mouse	1A8	AB_1877262	127617	BioLegend	1/1000
MMR	APC	mouse	C068C 2	AB_10896057	141707	BioLegend	1/1000
MMR	AF700	mouse	C068C 2	AB_2629637	141734	BioLegend	1/1000
SiglecF	AF700	mouse	1RNM4 4N	AB_2637126	56-1702-82	Invitrogen	1/1000

Table S2: antibodies for IHC, related to STAR methods.

Antigen	ID	Poly/mono	RRID	Manufacturer	Dilution
Chicken anti-GFP	Ab13970	Polyclonal	AB_300798	Abcam	1/500
Rat anti-CLEC12A	143402	5D3/CLEC12A	AB_11125370	BioLegend	1/100
Rabbit anti-IBA1	019-19741	Polyclonal	AB_839504	Wako	1/250
Goat anti-IBA1	Ab48004	Polyclonal	AB_870576	Abcam	1/250
Goat anti-Iba1	Ab5076	Polyclonal	AB_2224402	Abcam	1/250 (1/100

					Figure S12)
Rabbit anti-RFP	Ab62341	Polyclonal	AB_945213	Abcam	1/250
Rat anti-Lyve1	14-0443-82	ALY7	AB_1633414	eBioscience	1/100
Goat anti-MMR	AF2535	Polyclonal	AB_2063012	R&D systems	1/100
Mouse anti-human nucleus	MAB1281	235-1	AB_94090	Chemicon	1/200
Rabbit anti-P2RY12	AS-55043A	Polyclonal	AB_2298886	Anaspec	1/500
Rabbit anti-B-amyloid	8243S	D52D2	AB_2797642	Cell Signaling Technology	1/1000 (1/100 Figure S12)
Rat anti-CD31	550274	MEC 13.3	AB_394815	BD Biosciences	1/100
Rabbit anti-CD163	Ab182422	EPR19518	AB_2753196	Abcam	1/200
Rat anti-F4/80	MCA497GA	A3-1	AB_323806	Bio-Rad	1/250
Goat anti-Lyve1	AF2125	polyclonal	AB_2297188	R&D Systems	1/200
Human anti-human HLA-DR	130-122-299	REA805	AB_2801880	Miltenyi	1/50
Streptavidin-Alexa Fluor 750	S21384	/	/	Invitrogen	1/400
Ulex-biotin	B-1065-2	/	/	Vector Laboratories	1/200
Donkey anti-rabbit Alexa Fluor 647	A31573	Polyclonal	AB_2536183	Invitrogen	1/500 (1/400 Figure S12)
Donkey anti-goat Alexa Fluor 647	A21447	Polyclonal	AB_141844	Invitrogen	1/500
Donkey anti-goat Alexa Fluor 647	A32849	Polyclonal	AB_2762840	Invitrogen	1/500
Donkey anti-goat Alexa Fluor 568	A11057	Polyclonal	AB_142581	Invitrogen	1/500
Goat anti-rat Alexa Fluor 555	A21434	Polyclonal	AB_141733	Invitrogen	1/500
Donkey anti-rabbit Alexa Fluor 546	A10040	Polyclonal	AB_2534016	Invitrogen	1/500
Donkey anti-Rabbit 594	A21207	Polyclonal	AB_141637	Invitrogen	1/500
Donkey anti-rat Alexa Fluor 555 plus	A48270	Polyclonal	AB_2896336	Invitrogen	1/1000
Donkey anti-mouse Alexa Fluor 488	A21202	Polyclonal	AB_141607	Invitrogen	1/500
Donkey anti-chicken CF488A	SAB4600031	Polyclonal	AB_2721061	Merck	1/500
Goat anti-chicken Alexa Fluor 488	A11039	Polyclonal	AB_142924	Invitrogen	1/500
Donkey anti-Rabbit Alexa Fluor 488	A21206	Polyclonal	AB_2535792	Invitrogen	1/500
Donkey anti-goat Alexa Fluor 488	A11055	Polyclonal	AB_2534102	Invitrogen	1/500
Donkey anti-human DyLight 488	SA5-10126	Polyclonal	AB_2556706	Invitrogen	1/500
Donkey anti-goat Alexa Fluor 594	A11058	Polyclonal	AB_2534105	Invitrogen	1/400
Rabbit anti-B-amyloid-Alexa Fluor 488	51374S	D52D2	AB_2799392	Cell Signaling Technology	1/100

Table S4: mice used, related to STAR methods.

Figure	Strain	Group	age	n	Sex (M/F)
1A, S1A	*Flt3*^{Cre}:*Yfp*	Control	14w	6	M
	Flt3^{Cre}:*Yfp*	PLX	14w	7	M
1B	*Flt3*^{Cre}:*Yfp*	Control	5mo	4	M
	Flt3^{Cre}:*Yfp*	PLX	5mo	6	M
1C	*Flt3*^{Cre}:*Yfp*	PLX	10W	3	M
S1C,D	C57BL/6	PLX + Mo transfer	13W	4	M
1D,E,H, S1E,G	*Flt3*^{Cre}:*Yfp*	PLX	10-16W	5	M
	Flt3^{Cre}:*Yfp*	PLX+200RAD	10-16W	6	M
	Flt3^{Cre}:*Yfp*	PLX+400RAD	10-16W	7	M
	Flt3^{Cre}:*Yfp*	PLX+600RAD	10-16W	6	M
	Flt3^{Cre}:*Yfp*	PLX+800RAD	10-16W	6	M
	Flt3^{Cre}:*Yfp*	Naïve blood	4-6W	4	M
1F,G, S1F	*Flt3*^{Cre}:*Yfp*	PLX	10W	5	M
	Flt3^{Cre}:*Yfp*	PLX+600RAD	10W	5	M
1I,J	C57BL/6	Control	10W	3	1M/2F
	C57BL/6	1W PLX	10W	6	3M/3F
	C57BL/6	2W PLX	11W	6	3M/3F
1K,L	C57BL/6	Control	10W	3	1M/2F
	C57BL/6	1W PLX	10W	6	2M/4F
	C57BL/6	2W PLX	11W	6	2M/4F
2A, S2A	*Cx3cr1*^{CreER}:*Csf1r*$^{fl/fl}$	No tam	PD3	3	unknown
	Cx3cr1^{CreER}:*Csf1r*$^{fl/fl}$	Tam	PD3	2	unknown
	Cx3cr1^{CreER}:*Csf1r*$^{fl/fl}$	Mo transfer	PD3	2	unknown
	Cx3cr1^{CreER}:*Csf1r*$^{fl/fl}$	No tam	PD7	3	1M/2F
	Cx3cr1^{CreER}:*Csf1r*$^{fl/fl}$	Tam	PD7	3	1M/2F
	Cx3cr1^{CreER}:*Csf1r*$^{fl/fl}$	Mo transfer	PD7	3	unknown
	Cx3cr1^{CreER}:*Csf1r*$^{fl/fl}$	No Tam	PD16	4	1M/3F
	Cx3cr1^{CreER}:*Csf1r*$^{fl/fl}$	Tam	PD16	3	2F/1 unknown
	Cx3cr1^{CreER}:*Csf1r*$^{fl/fl}$	Mo transfer	PD16	8	3M/3F (2 unknown)
2B,C	*Cx3cr1*^{CreER}:*Csf1r*$^{fl/fl}$	Mglia transfer	PD16	9	4M/5F
	Cx3cr1^{CreER}:*Csf1r*$^{fl/fl}$	Mo transfer	PD16	6	3M/3F
	Cx3cr1^{CreER}:*Csf1r*$^{fl/fl}$	Mglia transfer	PD21	14	3M/11F
	Cx3cr1^{CreER}:*Csf1r*$^{fl/fl}$	Mo transfer	PD21	14	5M/5F 4 unknown
	Cx3cr1^{CreER}:*Csf1r*$^{fl/fl}$	Mglia transfer	3mo	12	5M/6F 1 unknown
	Cx3cr1^{CreER}:*Csf1r*$^{fl/fl}$	Mo transfer	3mo	9	4M/5F
	Cx3cr1^{CreER}:*Csf1r*$^{fl/fl}$	Mglia transfer	10mo	9	5M/4F
	Cx3cr1^{CreER}:*Csf1r*$^{fl/fl}$	Mo transfer	10mo	9	6M/3F
	Cx3cr1^{CreER}:*Csf1r*$^{fl/fl}$	Mglia transfer	12mo	5	1M/4F
	Cx3cr1^{CreER}:*Csf1r*$^{fl/fl}$	Mo transfer	12mo	10	3M/7F
	Cx3cr1^{CreER}:*Csf1r*$^{fl/fl}$	Mglia transfer	20mo	3	2M/1F
	Cx3cr1^{CreER}:*Csf1r*$^{fl/fl}$	Mo transfer	20mo	3	2M/1F
2D	*Cx3cr1*^{CreER}:*Csf1r*$^{fl/fl}$	No tam	PD16	4	1M/3F
	Cx3cr1^{CreER}:*Csf1r*$^{fl/fl}$	Mglia transfer	PD16	8	6M/2F
	Cx3cr1^{CreER}:*Csf1r*$^{fl/fl}$	Mo transfer	PD16	6	4M/2F
	Cx3cr1^{CreER}:*Csf1r*$^{fl/fl}$	Tam+sham	3mo	3	3M
	Cx3cr1^{CreER}:*Csf1r*$^{fl/fl}$	No tam	3mo	4	3M/1F
	Cx3cr1^{CreER}:*Csf1r*$^{fl/fl}$	Mglia transfer	3mo	6	3M/3F
	Cx3cr1^{CreER}:*Csf1r*$^{fl/fl}$	Mo transfer	3mo	4	2M/2F
	Cx3cr1^{CreER}:*Csf1r*$^{fl/fl}$	No tam	12mo	3	2M/1F
	Cx3cr1^{CreER}:*Csf1r*$^{fl/fl}$	Mglia transfer	12mo	4	1M/3F
	Cx3cr1^{CreER}:*Csf1r*$^{fl/fl}$	Mo transfer	12mo	5	2M/3F
2E,F	*Cx3cr1*^{CreER}:*Csf1r*$^{fl/fl}$	Naive	4W	10	7M/3F

	Cx3cr1^{CreER}:*Csf1r*$^{fl/fl}$	Tam	4W	6	5M/1F
	Cx3cr1^{CreER}:*Csf1r*$^{fl/fl}$	Mo transfer	4W	7	3M/4F
2G	*Cx3cr1*^{CreER}:*Csf1r*$^{fl/fl}$	No tam	PD16	2	1M/1F
	Cx3cr1^{CreER}:*Csf1r*$^{fl/fl}$	Tam	PD16	2	1M/1F
	Cx3cr1^{CreER}:*Csf1r*$^{fl/fl}$	Tam	3mo	3	1M/2F
S2B	*Cx3cr1*^{CreER}:*Csf1r*$^{fl/fl}$	No tam	3mo	2	1M/1F
	Cx3cr1^{CreER}:*Csf1r*$^{fl/fl}$	Mglia transfer	3mo	3	2M/1F
	Cx3cr1^{CreER}:*Csf1r*$^{fl/fl}$	Mo transfer	3mo	3	2M/1F
S2C,D	*Cx3cr1*^{CreER}:*Csf1r*$^{fl/fl}$:5xFAD$^{+/-}$	Mglia transfer	5mo	4	1M/3F
	Cx3cr1^{CreER}:*Csf1r*$^{fl/fl}$:5xFAD$^{+/-}$	Mo transfer	5mo	4	4F
S2E	*Cx3cr1*^{CreER}:*Csf1r*$^{fl/fl}$	Mo transfer	4W	2	1M/1F
3B, S2F,G,H	*Cx3cr1*^{CreER}:*Csf1r*$^{fl/fl}$	Control	10W	7	4M/3F
	Cx3cr1^{CreER}:*Csf1r*$^{fl/fl}$	Tam C	10W	7	3M/4F
	Cx3cr1^{CreER}:*Csf1r*$^{fl/fl}$	Tam CI	10W	6	4M/2F
	Cx3cr1^{CreER}:*Csf1r*$^{fl/fl}$	1W PLX+Tam CI	10W	5	3M/2F
	Cx3cr1^{CreER}:*Csf1r*$^{fl/fl}$	2W PLX+Tam CI	11W	7	5M/2F
S2I	C57BL/6	Control	10W	5	1M/4F
	Cx3cr1^{CreER}:*Csf1r*$^{fl/fl}$	PLX+Tam CI	10-11W	5	3M/2F
3C,D	*Cx3cr1*^{CreER}:*Csf1r*$^{fl/fl}$	Control	10W	4	2M/2F
	Cx3cr1^{CreER}:*Csf1r*$^{fl/fl}$	Tam C	10W	4	3M/1F
	Cx3cr1^{CreER}:*Csf1r*$^{fl/fl}$	Tam CI	10W	7	4M/3F
	Cx3cr1^{CreER}:*Csf1r*$^{fl/fl}$	1W PLX+Tam CI	10W	6	5M/1F
	Cx3cr1^{CreER}:*Csf1r*$^{fl/fl}$	2W PLX+Tam CI	11W	7	4M/3F
3E	*Cx3cr1*^{CreER}:*Csf1r*$^{fl/fl}$	Naive	7W	4	4F
	Cx3cr1^{CreER}:*Csf1r*$^{fl/fl}$	PLX	7W	4	2M/2F
	Cx3cr1^{CreER}:*Csf1r*$^{fl/fl}$	PLX + Tam CI	7W	4	4M
3F-I, S2J	*Cx3cr1*^{CreER}:*Csf1r*$^{fl/fl}$	1W PLX+Tam CI	10W	4	4M
4A-H,S4A-C, S3A-C,F-K	*Flt3*^{Cre}:*Yfp*	Control	14W	8	M
	Flt3^{Cre}:*Yfp*	2W PLX	14W	7	M
S3D,E	C57BL/6	Control	10W	4	M
	Flt3^{Cre}:*Yfp*	PLX	10W	4	M
4I-O, S4A-K	*Cx3cr1*^{CreER}:*Csf1r*$^{fl/fl}$	adult	14W	4	M
	Cx3cr1^{CreER}:*Csf1r*$^{fl/fl}$	neonate	9W	3	F
5A-G,S4L-P	*Cx3cr1*^{CreER}:*Csf1r*$^{fl/fl}$	MDP-Mo-Mglia	6W	3	2M/1F
	Cx3cr1^{CreER}:*Csf1r*$^{fl/fl}$	GMP-Mo-Mglia	6W	3	2M/1F
5H-N, S4Q-V	*Cx3cr1*^{CreER}:*Csf1r*$^{fl/fl}$	FL neonates	9W	5	M
6, S5	C57BL/6	Fetal liver	E14	27	M/F
	C57BL/6	Bone marrow	10W	3	2M/1F
7A-G, S6B-I, L-R	*hCSF1*^{KI} x *Rag2*$^{-/-}$ x $\gamma c^{/-}$	Adult blood	4W	9	5M/4F
	hCSF1^{KI} x *Rag2*$^{-/-}$ x $\gamma c^{-/-}$	Cord blood	4W	8	3M/5F
7D,E	*hCSF1*^{KI} x *Rag2*$^{-/-}$ x $\gamma c^{-/-}$	Adult blood	4W	3	1M/2F
S6J,K	*hCSF1*^{KI} x *Rag2*$^{-/-}$ x $\gamma c^{-/-}$	Cord blood	4W	3	2M/1F
